# Supplementary material for: On the relative motions of long-lived Pacific mantle plumes
Source: Nat Commun. 2018 Feb 27;9:854. doi: 10.1038/s41467-018-03277-x (PMC5829163; doi:10.1038/s41467-018-03277-x)
Supplement: Supplementary file 1 — Supplementary Information [file 41467_2018_3277_MOESM1_ESM.pdf]

Supplementary Information

On the Relative Motions of Long-lived Pacific Mantle Plumes

Kevin Konrad[1*], Anthony A.P. Koppers[1], Bernhard Steinberger[2,3], Valerie Finlayson[4], Jasper Konter[4], Matthew G. Jackson[5]

[1]College of Earth, Ocean, and Atmospheric Sciences, Oregon State University, Corvallis, OR, USA.

[2]GFZ German Research Centre for Geosciences, D-14473 Potsdam, Germany.

[3]Centre for Earth Evolution and Dynamics (CEED), University of Oslo, 0315 Oslo, Norway.

[4]Department of Geology and Geophysics, School of Ocean and Earth Science and Technology, University of Hawaii, Manoa, Honolulu, HI, USA.

[5]Department of Earth Science, UC Santa Barbara, Santa Barbara, CA, USA.

*Corresponding author: Konradke@oregonstate.edu

This PDF file includes:

Supplementary Note 1

Supplementary Discussion

Supplementary Figures 1 – 5

Supplementary Tables 1 – 3

Supplementary Note 2

Supplementary Note 1

Supplementary $^{40}Ar/^{39}Ar$ Age Determinations Notes

In total 31 age dates for separates are reported here, producing 21 reliable plateau dates representing the eruption ages with $^{40}Ar/^{39}Ar$ isochron intercepts within error or very close to atmosphere (295.5), 4 samples containing clear and correctable excess Ar patterns still resulting in acceptable eruption ages, and 6 separates with ages deemed undeterminable. Below is a discussion of the age determinations for lava flows from each seamount with all uncertainties reported at the 2σ confidence level. See the accompanying age results supplement for the incremental heating age and K/Ca spectra diagrams along with inverse isochrons, full sample information and plateau justifications.

The first seamount analyzed is Manu Lele Vai (D02), which contained altered aphanitic basalts and ankaramites with calcite vesicle infilling. Sample D02-04 (74.57 ± 0.28 Ma) produced a relatively short plateau (30% ^{39}Ar) during the low-mid temperature effects wherein the apparent age becomes increasingly younger, likely due to high temperature recoil. The sample is highly radiogenic and thus the isochron points are clustered resulting in a poor intercept value of 445 ± 145. Sample D02-05 was deemed unreliable due to the high scatter (MSWD > 5). Sample D02-17 produced an interesting spectrum with two potentially short plateaus developed at ~67 and ~57 Ma. Neither plateau produced an atmospheric isochron, and due to the potential for two different age interpretations this sample is declared unreliable.

One aphanitic lava flow from the seamount Taring Nui (D03) was analyzed twice with two different leaching procedures. The first split was leached with the standard one hour acid baths described above and the analysis was deemed unreliable having high atmospheric argon, strong recoil effects and generating two potential plateaus with non-atmospheric intercepts. The second split was leached with two hour HCl steps followed by the standard HNO_3 and DI H_2O steps. The sample was then picked with much greater rigidity producing only 3 mg of material. The resulting plateau is flat and covers 40% of the released ^{39}Ar, contains an atmospheric intercept, and a MSWD of 0.75. Thus, this flow provides a single age constraint for this seamount at 61.57 ± 0.5 Ma. One sample from Logotau (D04) contained plagioclase phenocrysts and provided an age of 63.66 ± 0.34 Ma along with an atmospheric intercept and MSWD of 1.3.

Multiple splits from two basalts were analyzed for Tefolaha (D07). Sample D07-09 had a plagioclase analysis with an age of 51.01 ± 0.36 Ma that contained an atmospheric intercept (293.5 ± 9.5) and wide plateau (73% ^{39}Ar) until higher temperature steps wherein distinct increases in apparent age occurred likely due to the excess ^{40}Ar released from deep seated inclusions. This sample contained very fresh clinopyroxene so a 25 mg separate was analyzed, providing an age of 49.25 ± 2.48 Ma that meets all the criteria for a reliable age, but is less precise due to its low potassium concentration. The resulting combined plateau age for D07-09 is 51.00 ± 0.31 Ma. A pyroxene and plagioclase phyric clast from the hyaloclastite sample D07-22 was analyzed three times with two splits of groundmass and a plagioclase separate, all from the same irradiation. The first groundmass separate was accidentally pre-cleaned with a higher power CO_2 beam resulting in a large loss of gas from the low temperature heating spectrum. The sample still produced a fairly long (51%) plateau containing an age of 54.65 ± 0.12 Ma with an intercept of 419 ± 184. A second split of the groundmass was analyzed with a reliable but more scattered plateau age of 54.95 ± 0.14 Ma. The highly radiogenic nature of the sample caused all the plateau points to cluster tightly and thus the ^{40}Ar/^{36}Ar intercept was not obtainable. The plagioclase separate from this sample produced a long (94% ^{39}Ar) plateau with an atmospheric intercept and younger age of 53.64 ± 0.18 Ma. Since the groundmass did not provide a reasonable intercept for both experiments (492 ± 197 and -221 ± 444). These non-atmospheric intercepts thus provide a misleadingly old age for the groundmass samples and the plagioclase values are used in the models and discussions herein.

A groundmass split from sample D10-04, a small and glassy basalt from Nui (D10), was attempted. The experiment produced an apparently young and humped heating spectrum with no reliable age determinable. Trace amounts of hornblende were separated from the basalt D11-10 (Laupapa Seamount) and two small splits (~1 mg each) were analyzed. Both splits produced long (>98%) plateaus with relatively high uncertainties and neither sample degassed until the higher temperature steps. The stacked plateau results in an age of 52.86 ± 0.77 Ma; MSWD of 0.73; intercept of 286 ± 32. For the seamount Tayasa (D13), a plagioclase and groundmass separate from the sample D13-01 was attempted. The groundmass spectrum produced a continuous recoil pattern with the age starting apparently old and becoming increasing younger until the higher temperature steps wherein small amounts of excess ^{40}Ar increase the age. The plagioclase separate produced a long and reliable plateau with a corresponding age of 50.52 ± 0.20 Ma.

Two samples were analyzed from the seamount Nukufetau (D14). A groundmass separate was analyzed for sample D14-01, which produced a short (33% ^{39}Ar) plateau with an atmospheric intercept and MSWD of 0.58 resulting in an age of 43.64 ± 0.57 Ma. A plagioclase and groundmass separate from sample D14-08 were analyzed with a resulting age of 49.82 ± 0.18 Ma and 48.53 ± 0.17 Ma, respectively. The plagioclase heating spectrum from D14-08 contained two clear spikes in apparent age, a common sign of releasing excess Ar from melt/fluid inclusions (see $^{40}Ar/^{39}Ar$ Age Results), however the resulting plateau intercept was within error of atmosphere (300 ± 11). The plateau age for the groundmass split was recalculated with an $^{40}Ar/^{36}Ar$ intercept value of 342 ± 4 (2σ; n=24). The justifications and methods employed in this correction are discussed in the method section. The recalibrated groundmass age and plagioclase age (calculated assuming the standard 295.5 $^{40}Ar/^{36}Ar$ trapped argon ratio) are offset by ~1 Ma. These results are of an obvious concern when attempting to understand the age at which a lava flow erupted upon the seamount. This discrepancy may be due to some minor excess Ar in the plagioclase phase, which causes an apparent age that is too old.

Two samples from dredge 15 (Vaitupu Seamount) were analyzed. A groundmass separate from the basalt D15-02 produced a long (65% ^{39}Ar) plateau with a slightly above atmospheric intercept of 359 ± 39 and a resulting age of 49.03 ± 0.19 Ma. Sample D15-12 was analyzed twice due to the same pre-heating mistake that afflicted D07-22b. The first analyses did not produce a useable age determination while the second attempt contained a correctable low temperature excess Ar intercept (360.7 ± 5.8; n=19) and was recalculated to provide an age of 49.58 ± 0.18 Ma.

One plagioclase separate from a Telematua Seamount basalt (D16-35) was analyzed providing an age constraint of 46.63 ± 0.49 Ma (MSWD of 0.26; $^{40}Ar/^{36}Ar_{int}$ of 232.5 ± 164). Two hornblende separates were attempted from Funafuti (D18) producing concordant long age spectrums, low MSWD's and atmospheric intercepts with plateau ages of 48.88 ± 0.12 Ma (D18-07) and 48.93 ± 0.12 Ma (D18-23). Due to the similar lithologic character and age determinations it is possible that these samples represent two chunks broken off of the same lava flow. One groundmass separate was analyzed from the sample D22-29 (Silaga Seamount) producing a short (33% ^{39}Ar) but useable age constraint of 46.09 ± 0.28 Ma for this seamount.

Dredges 24 sampled Kosciusko Seamount and recovered numerous small (<25 cm) basaltic clasts within Mn nodules. Two small glassy and fairly fresh looking (no thin section was made) aphyric basalt clasts from D24 were analyzed. Groundmass splits from D24-04 (47.37 ± 0.11 Ma) were calculated using a low-temperature non-atmospheric intercept value of 499 ± 6 (n=24). The groundmass separate from D24-11 also contained a consistent low temperature excess argon pattern and thus the plateau age was recalculated using an intercept of 415 ± 51 producing an age of 48.16 ± 0.19 Ma. Multiple samples from D27 were analyzed with results from two samples reported herein. The majority of samples from this dredge are 11-15 Ma in age and are attributed to Samoan hotspot volcanism. Those results along with more samples not related to the Rurutu hotspot from the RR1310 expedition will be presented in an upcoming study. The two samples presented herein are a hornblende separate from D27-35, which produced a long plateau with a very reliable age of 42.24 ± 0.82 Ma. Both a hornblende and plagioclase separate were attempted from D27-64 with the hornblende producing a long, excellent heating spectrum and a resulting age of 45.15 ± 0.12 Ma. The plagioclase separate consistently increases in apparent age until the highest temperature steps wherein a very short (17.48 ^{39}Ar%) plateau is produced. Thus, an age of 45.73 ± 0.14 Ma for the separate is tentatively presented but is less reliable and the hornblende age is accepted as the more accurate eruption age constraint.

Supplementary Discussion

The Source of the Tuvalu Seamounts

The mantle source and origin associated with generating the Tuvalu Seamounts hitherto were poorly constrained with arguments either in favor of a Rurutu Hotspot origin[1] or an extinct hotspot[2]. In an effort to better understand the origin of these seamounts we invoke an ‘isotopic finger printing’ technique[1,3-6] and a ‘backtracking’ technique[1,5-7]. Of the reported Tuvalu Seamounts, all but two contain distinct HIMU isotopic signatures (high time integrated U/Pb; defined herein as ^{206}Pb/^{204}Pb >20; **Supplementary Table 2**) that are similar to the chemical signatures found in more modern Rurutu hotspot fed seamounts in French Polynesia[8]. The exceptions are Manu Lele Vai (D02; 75 Ma) and Silaga (D22; 46 Ma) that plot nearer to a ‘FOZO’ (focal zone)[9] composition. It is possible that the HIMU Rurutu hotspot could have provided the melts to these seamounts, as ocean island volcanoes (OIV) typically tend to produce an isotopic

mixing trend between one of several possible enriched endmember compositions and FOZO[9,10]. However, due to the uncertainty of Manu Lele Vai and Silaga being fed by an HIMU mantle plume, these two seamounts conservatively are not used in this study's hotspot track reconstruction models.

To investigate the potential deep mantle sources that fed the Tuvalu volcanoes we first compared the along-track age-distance relationship between the Rurutu-aged HIMU seamounts in the Cook-Australs[11-18], Tuvalu (this study) and Gilbert Ridge[1,6] and a variety of absolute plate motion (APM) models (**Supplementary Figure 2**). The APM models shown here cover fixed hotspots from Duncan and Clague [19], the preferred model in Koppers, et al. [20] and WK08-G from Wessel and Kroenke [2]. In addition is the mobile hotspot model PAC-MHS of Steinberger and Gaina [21] and the global moving hotspot reference model of Doubrovine, et al. [22]. Finally, included is the plate circuit through Antarctica model of Raymond, et al. [23] for the Pacific (**Supplementary Figures 2, 3**). With the exception of the plate circuit based APM model[23], both fixed and mobile hotspot models generally path through the Tuvalu region. It is important to note that no one APM model should exactly fit the Rurutu hotspot as the models are based off assumptions on hotspot fixity or modeled Hawaiian/Louisville plume motion.

To further investigate the mantle sources sourcing the Tuvalu volcanoes we employed a technique known as backtracking, wherein a seamount of a given age is traced back to the latitude and longitude the seamount would have been located at time of formation. Previous studies utilized this technique on the HIMU seamounts found within the Gilbert Ridge (immediately north of the Tuvalu Islands) and argued that independent of the model used (K01[20]; K04[24]; S00[25]; R00[23]; or Wessel, et al. [7]) that these seamounts generally cluster around the isotopically similar modern day Rurutu hotspot[1]. **Supplementary Figure 4** shows the same method used on the new age data from the Tuvalu seamounts employing the WK08 plate motion model[2] (red line in **Supplementary Figures 2, 3**) while other models provide very similar results (not shown). In agreement with the Gilbert Ridge results, the Tuvalu seamounts trace back to locations near the Rurutu hotspot, currently assumed to be underlying Arago Seamount (23.44^{o}S, 150.7^{o} W)[11]. The samples plot clearly too far west (~1100 km) of the MacDonald hotspot to be reasonably associated with that mantle anomaly. Important outliers that are not included in the model or **Figure 1**, include Manu Lele Vai sample RR1310-D02-04 (75 Ma) and RR1310-D27-35 (42 Ma) from Nuilakita, both of

which do not reasonably correlate with the location of the Rurutu hotspot. Manu Lele Vai appears backtracked similarly to the isotopically EMI-like (enriched mantle I) seamounts Ava (78 Ma) and Sakau (75 Ma) found within the Gilbert Ridge that do not correlate to any known modern hotspots[1,6]. Nuilakita is a complex seamount with evidence for both older French Polynesian volcanism (at 42 Ma) and younger Samoan hotspot volcanism at ca. 14 Ma. Therefore, it is uncertain whether the 42 Ma age represents an episode of volcanism from the Rurutu hotspot or volcanism sourced from a different hotspot and is not considered in the presented models. The combination of plate motion based regressions and isotopic ‘finger printing’ provide strong evidence for the Rurutu Hotspot being the mantle anomaly which sourced the majority of the HIMU-type Tuvalu seamounts.

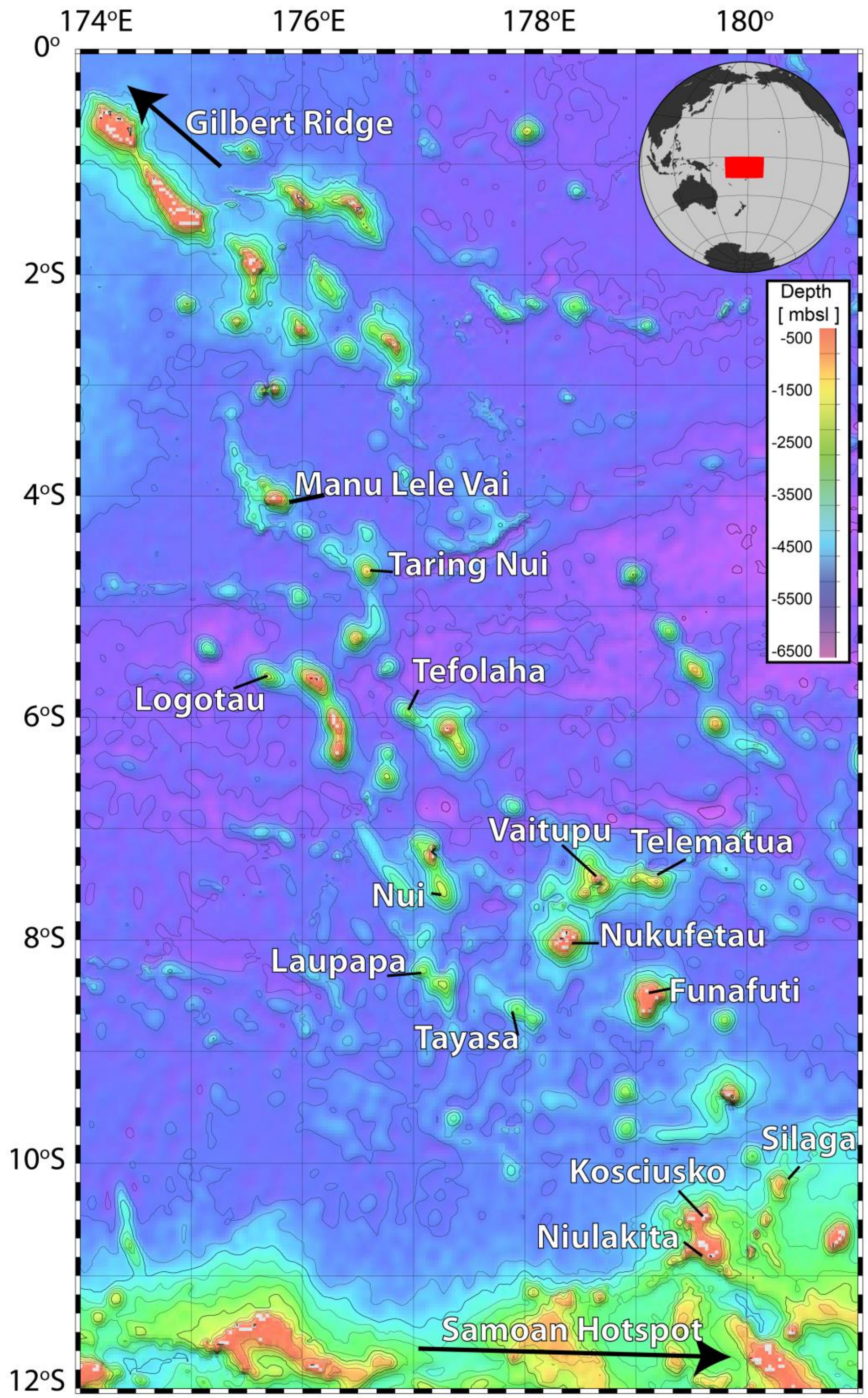

Supplementary Figure 1: A bathymetric map of the Tuvalu Seamount region with seamounts analyzed in this study labeled. Bathymetric map was generated using the seamount catalog program[26], with data collected during the RR1310 expedition (see methods) merged with predicted seafloor bathymetry from Smith and Sandwell [27].

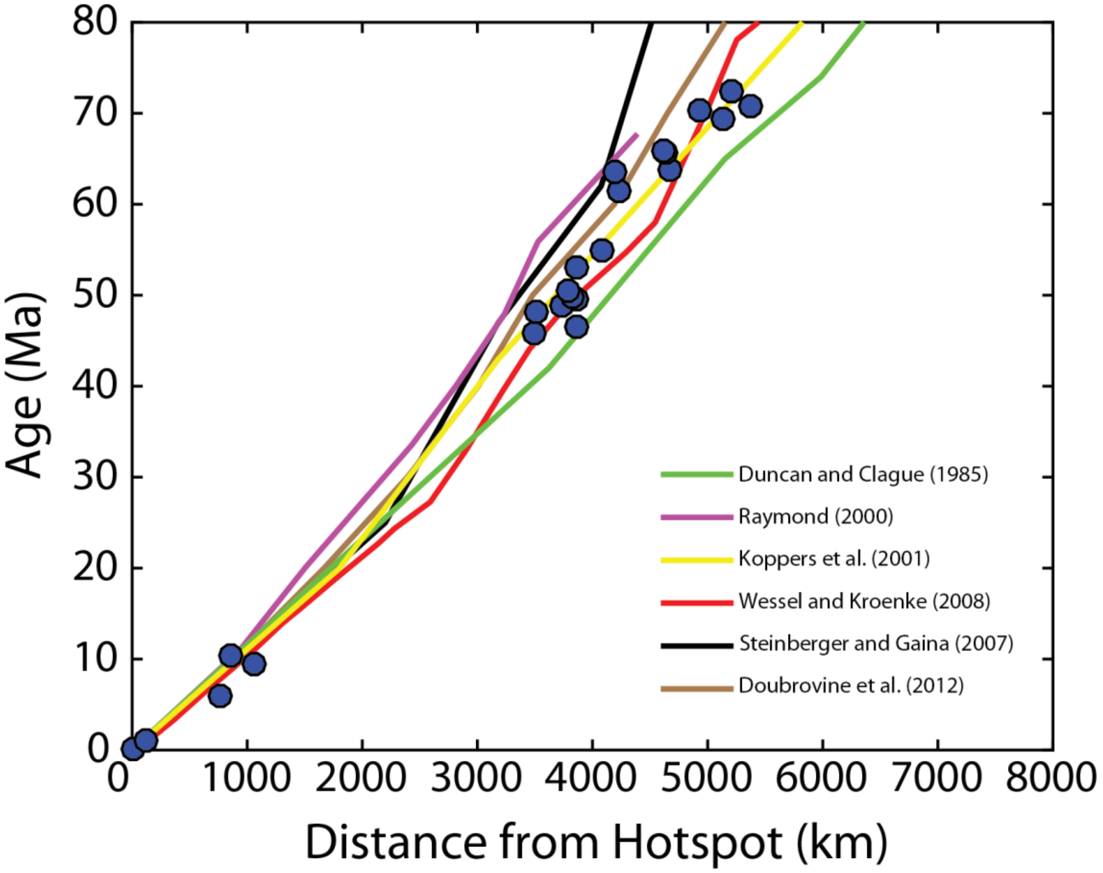

Supplementary Figure 2: Along track distance from the Rurutu hotspot (Arago Seamount) as a function of age. Shown in blue are the HIMU seamounts that are interpreted to have been source from the Rurutu hotspot (see text for sources). The individual lines represent different APM models for the Pacific including the fixed hotspot models of Duncan and Clague [19], Koppers, et al. [20], and Wessel and Kroenke [2], the moving hotspot models of Steinberger and Gaina [21] and Doubrovine, et al. [22], along with the plate circuit model of Raymond, et al. [23]. For the moving hotspot models, the misfit with the older age dates indicates a plume motion with a southeast component, consistent with the modelled motion shown in **Figure 2**.

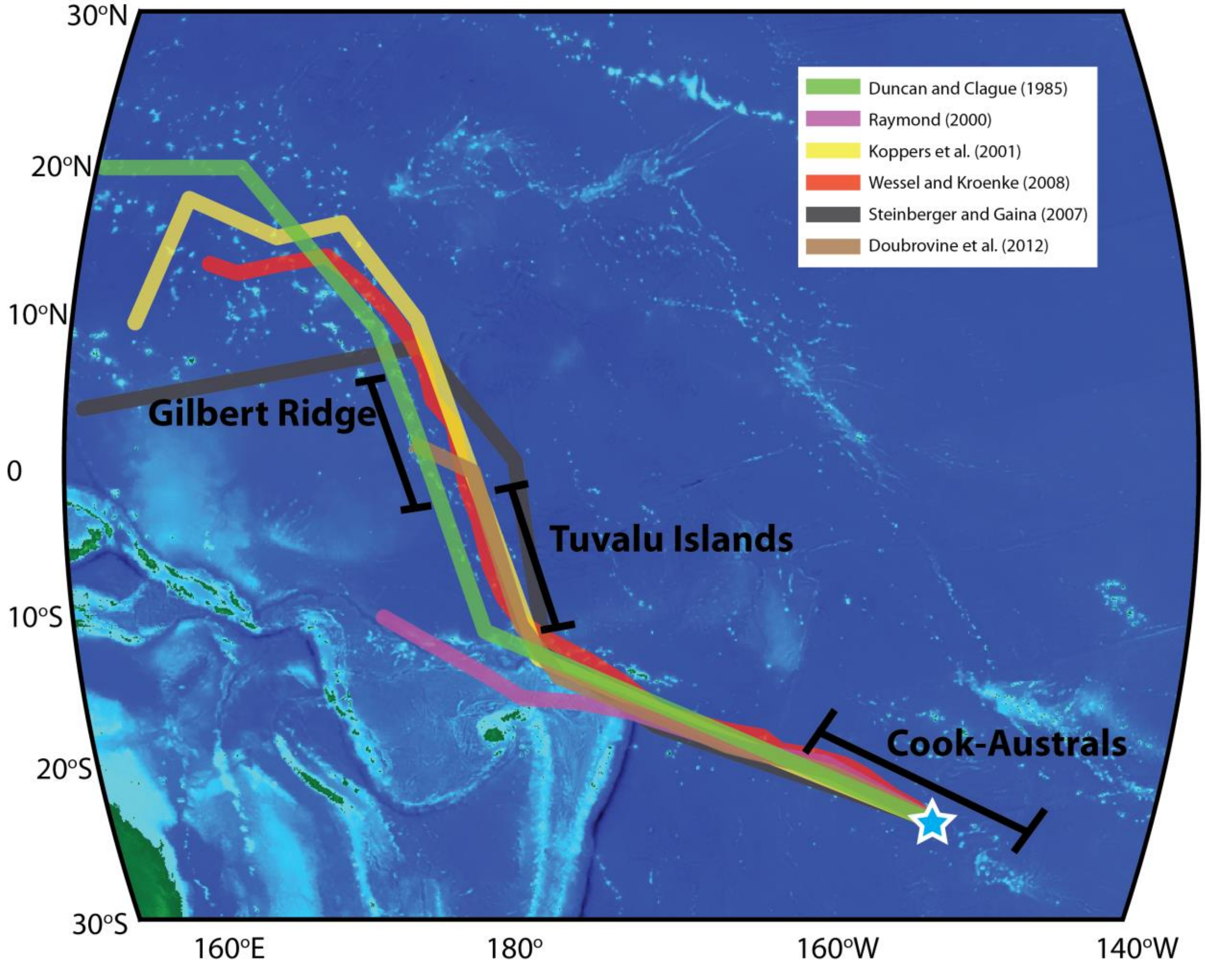

Supplementary Figure 3: The geometry of absolute plate motion model tracks initiating from the inferred Rurutu hotspot (Arago Seamount). The APM models are the same as in **Supplementary Figure 2**. Tracks are rotated backwards to 150 Ma for Duncan and Clague [19], 67.74 Ma for Raymond, et al. [23], 140 Ma for Koppers, et al. [20], 106.8 Ma for Wessel and Kroenke [2], 150 Ma for Steinberger and Gaina [21] and 80 Ma for Doubrovine, et al. [22]. The bathymetric map was generated using the ETOPO1[28] dataset.

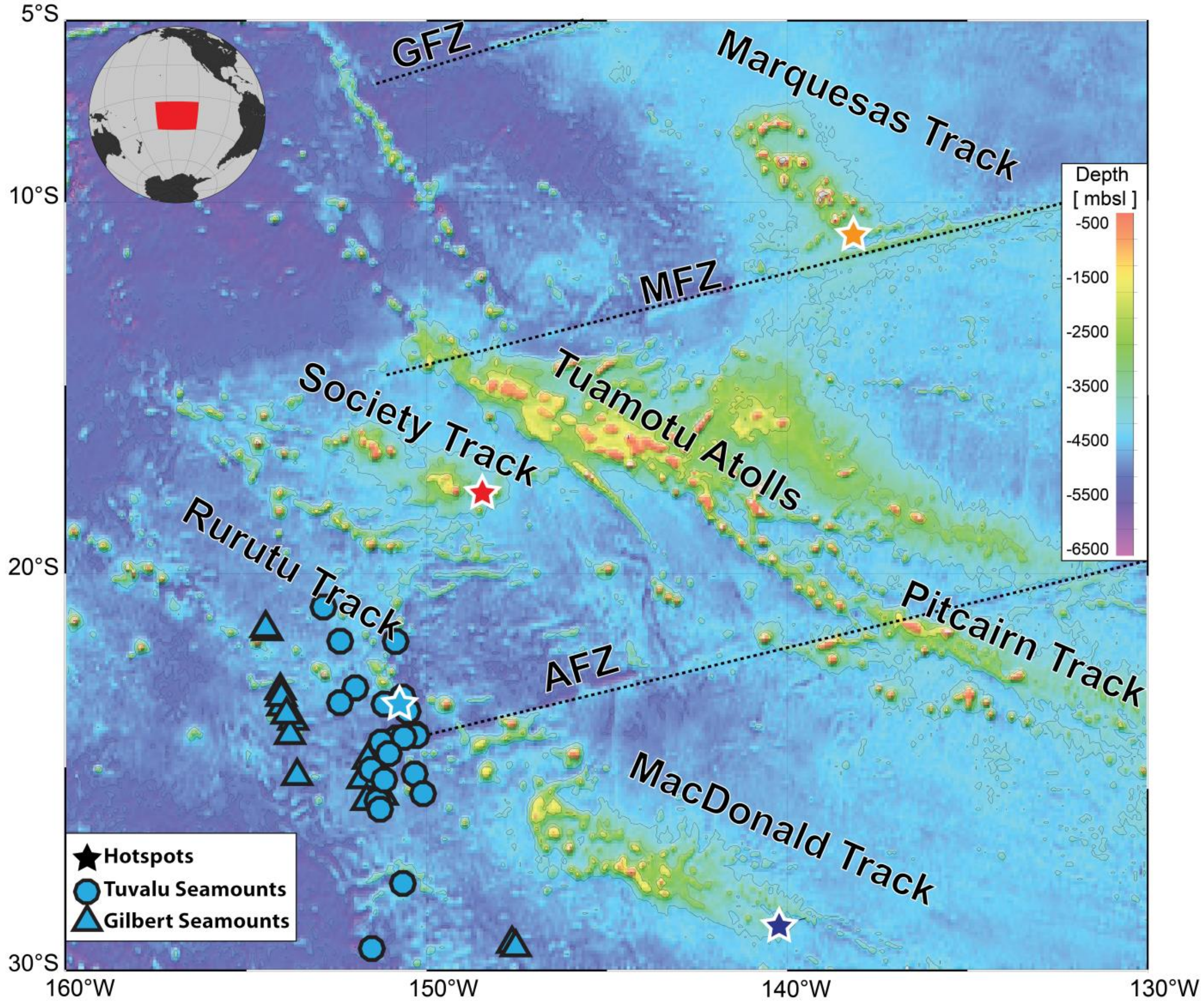

Supplementary Figure 4: The results of backtracking Rurutu hotspot seamounts. The Tuvalu Seamounts (circles) and Gilbert Ridge (triangles) were backtracked with the APM model WK-08G[2]. The results are superimposed on a bathymetric map (generated with seamount catalog[26]) of the South Pacific Isotopic and Thermal Anomaly (SOPITA) region with hotspot tracks labeled and the corresponding hotspot location marked with a star. Major fracture zones are indicated with dashed lines and GFZ, MFZ and AFZ stand for the Galapagos, Marquesas and Austral fracture zones, respectively.

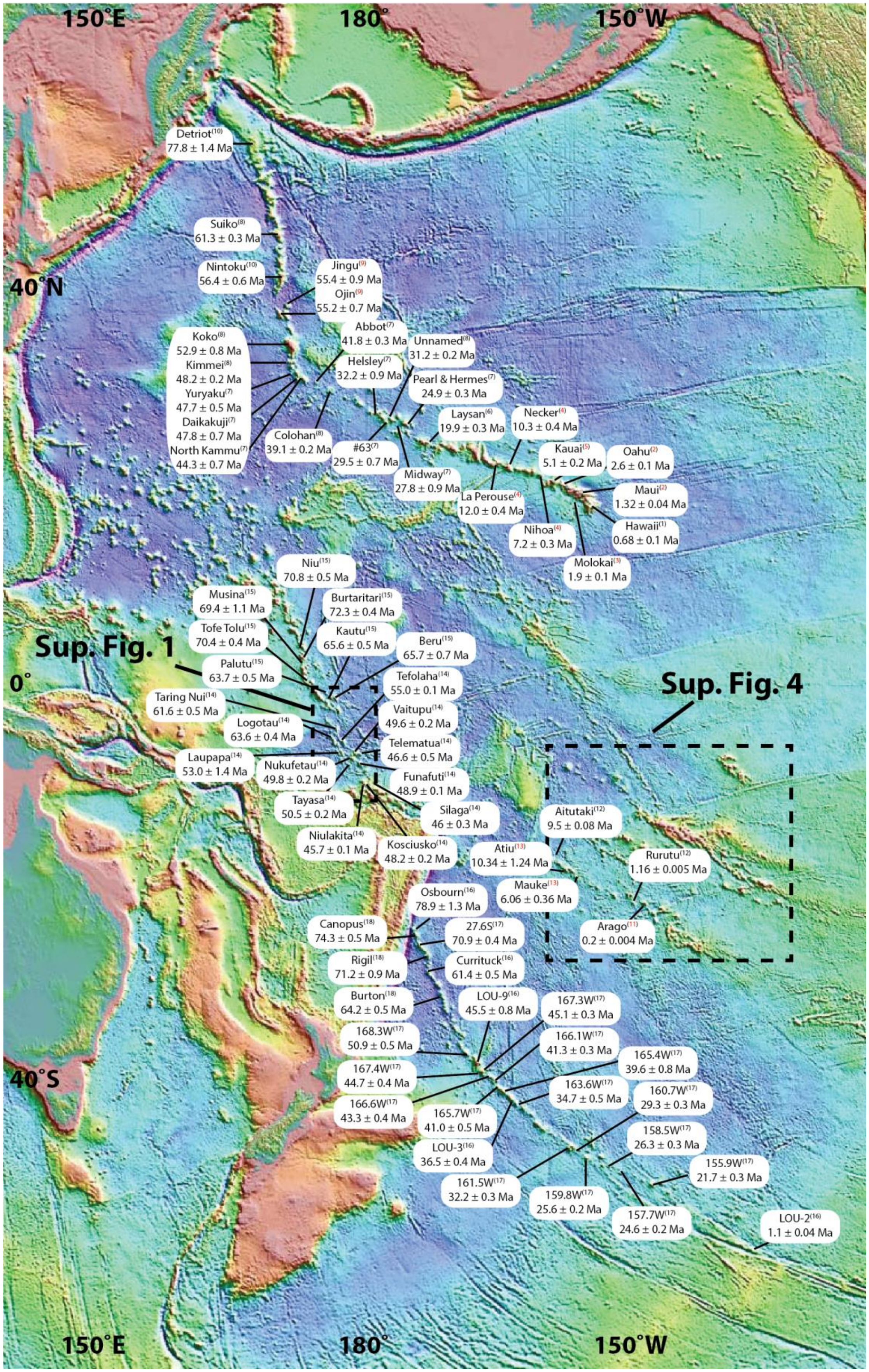
150°E
180°
150°W
40°N
0°
40°S
Detriot[10]
77.8 ± 1.4 Ma
Suiko[8]
61.3 ± 0.3 Ma
Nintoku[10]
56.4 ± 0.6 Ma
Jingu[9]
55.4 ± 0.9 Ma
Ojin[9]
55.2 ± 0.7 Ma
Abbot[7]
41.8 ± 0.3 Ma
Unnamed[8]
31.2 ± 0.2 Ma
Koko[8]
52.9 ± 0.8 Ma
Kimmei[8]
48.2 ± 0.2 Ma
Yuryaku[7]
47.7 ± 0.5 Ma
Daikakuji[7]
47.8 ± 0.7 Ma
North Kammu[7]
44.3 ± 0.7 Ma
Helsley[7]
32.2 ± 0.9 Ma
Pearl & Hermes[7]
24.9 ± 0.3 Ma
Colohan[8]
39.1 ± 0.2 Ma
#63[7]
29.5 ± 0.7 Ma
Laysan[6]
19.9 ± 0.3 Ma
Necker[4]
10.3 ± 0.4 Ma
Midway[7]
27.8 ± 0.9 Ma
Kauai[5]
5.1 ± 0.2 Ma
Oahu[2]
2.6 ± 0.1 Ma
La Perouse[4]
12.0 ± 0.4 Ma
Maui[2]
1.32 ± 0.04 Ma
Hawaii[1]
0.68 ± 0.1 Ma
Nihoa[4]
7.2 ± 0.3 Ma
Molokai[3]
1.9 ± 0.1 Ma
Niu[15]
70.8 ± 0.5 Ma
Musina[15]
69.4 ± 1.1 Ma
Burtaritari[15]
72.3 ± 0.4 Ma
Tofe Tolu[15]
70.4 ± 0.4 Ma
Kautu[15]
65.6 ± 0.5 Ma
Beru[15]
65.7 ± 0.7 Ma
Sup. Fig. 1
Palutu[15]
63.7 ± 0.5 Ma
Tefolaha[14]
55.0 ± 0.1 Ma
Sup. Fig. 4
Taring Nui[14]
61.6 ± 0.5 Ma
Vaitupu[14]
49.6 ± 0.2 Ma
Logotau[14]
63.6 ± 0.4 Ma
Telematua[14]
46.6 ± 0.5 Ma
Laupapa[14]
53.0 ± 1.4 Ma
Nukufetau[14]
49.8 ± 0.2 Ma
Funafuti[14]
48.9 ± 0.1 Ma
Tayasa[14]
50.5 ± 0.2 Ma
Silaga[14]
46 ± 0.3 Ma
Aitutaki[12]
9.5 ± 0.08 Ma
Niulakita[14]
45.7 ± 0.1 Ma
Kosciusko[14]
48.2 ± 0.2 Ma
Atiu[13]
10.34 ± 1.24 Ma
Rurutu[12]
1.16 ± 0.005 Ma
Mauke[13]
6.06 ± 0.36 Ma
Osbourn[16]
78.9 ± 1.3 Ma
Canopus[18]
74.3 ± 0.5 Ma
27.6S[17]
70.9 ± 0.4 Ma
Arago[11]
0.2 ± 0.004 Ma
Rigil[18]
71.2 ± 0.9 Ma
Currituck[16]
61.4 ± 0.5 Ma
Burton[18]
64.2 ± 0.5 Ma
LOU-9[16]
45.5 ± 0.8 Ma
167.3W[17]
45.1 ± 0.3 Ma
168.3W[17]
50.9 ± 0.5 Ma
166.1W[17]
41.3 ± 0.3 Ma
165.4W[17]
39.6 ± 0.8 Ma
167.4W[17]
44.7 ± 0.4 Ma
163.6W[17]
34.7 ± 0.5 Ma
160.7W[17]
29.3 ± 0.3 Ma
166.6W[17]
43.3 ± 0.4 Ma
165.7W[17]
41.0 ± 0.5 Ma
158.5W[17]
26.3 ± 0.3 Ma
LOU-3[16]
36.5 ± 0.4 Ma
155.9W[17]
21.7 ± 0.3 Ma
161.5W[17]
32.2 ± 0.3 Ma
159.8W[17]
25.6 ± 0.2 Ma
157.7W[17]
24.6 ± 0.2 Ma
LOU-2[16]
1.1 ± 0.04 Ma
150°E
180°
150°W

Supplementary Figure 5: A satellite bathymetric map of the Pacific Basin showing the location and ages of the seamounts used in this study. Bathymetric map generated using seamount catalog[26] with bathymetric data from Smith and Sandwell [27]. Superscripted numbers represent the source of the age used; red numbers are K-Ar ages while black numbers are $^{40}Ar/^{39}Ar$ ages. (1) Sharp and Renne [29], (2) McDougall [30], (3) Naughton, et al. [31], (4) Dalrymple, et al. [32], (5) McDougall [33], (6) Dalrymple, et al. [34], (7) O'Connor, et al. [35], (8) Sharp and Clague [36], (9) Dalrymple and Garcia [37], (10) Duncan and Keller [38], (11) Bonneville, et al. [11], (12) Rose [17], (13) Turner and Jarrard [18], (14) This study, (15) Koppers, et al. [6], (16) Koppers, et al. [24], (17) Koppers, et al. [39], and (18) Koppers, et al. [40]. The geographic locations of **Supplementary Figures 1** (Tuvalu seamounts) and **3** (SOPITA region) are shown outlined with dashed lines.

Supplementary Table 1: $^{40}Ar/^{39}Ar$ age determinations for Tuvalu lava flows

Sample Information					Plateau									Inverse Isochron					
**Sample Name**	Material	Seamount	Lat ºS	Long ºE	**Age Ma**	**±2σ(i) Ma**	±2σ(f) Ma	^{39}Ar %	K/Ca	± 2σ	MSWD	n	N	Age Ma	±2σ(i) Ma	±2σ(f) Ma	$^{40}Ar/^{36}Ar$ intercept	± 2σ	MSWD
**RR1310-D02-04**	GM	Manu Lele Vai	3.906	175.630	**74.57**	**0.28**	1.69	30	0.406	0.052	3.18	10	43	74.2	0.48	1.73	445.12	144.89	2.45
**RR1310-D02-05**	GM	Manu Lele Vai	3.906	175.630									36						
**RR1310-D02-17**	GM	Manu Lele Vai	3.906	175.630									43						
**RR1310-D03-23**	GM	Taring Nui	4.669	176.567									37						
**RR1310-D03-23**	GM	Taring Nui	4.669	176.567	**61.57**	**0.5**	1.47	40	0.057	0.006	0.75	16	37	61.86	1	1.71	256.78	100.82	0.77
**RR1310-D04-1d**	PLG	Logotau	5.610	175.692	**63.66**	**0.34**	1.46	52	0.013	0	1.28	10	26	63.97	0.84	1.66	268.88	65.82	1.35
**RR1310-D07-09**	CPX	Tefolaha	5.968	176.960	**49.25**	**2.48**	2.72	71	0.07	0.008	0.9	7	14	48.66	3.39	3.56	297.6	5.3	0.96
**RR1310-D07-09**	PLG	Tefolaha	5.968	176.960	**51.01**	**0.36**	1.2	73	0.005	0	3.68	18	32	51.16	0.75	1.37	293.53	9.53	3.92
**RR1310-D07-09**	**ALL**	Tefolaha	5.968	176.960	**51**	**0.31**	1.18	72	0.005	0.0002	2.91	25	46	51.09	0.5	1.25	294.5	5.54	3.03
**RR1310-D07-22b**	GM	Tefolaha	5.968	176.960	**54.65**	**0.12**	1.23	51	0.575	0.033	1.42	8	33	54.5	0.27	1.25	419.29	184.14	1.27
**RR1310-D07-22b**	GM	Tefolaha	5.968	176.960	**54.95**	**0.14**	1.24	40	0.516	0.05	3.68	10	36	55.77	0.4	1.31	-813.47	557.04	0.61
**RR1310-D07-22b**	PLG	Tefolaha	5.968	176.960	**53.64**	**0.18**	1.22	95	0.021	0.001	1.65	19	26	53.74	0.23	1.23	284.27	15.97	1.56
**RR1310-D10-04**	GM	Nui											36						
**RR1310-D11-10**	AMP	Laupapa	8.270	177.063	**52.80**	**0.92**	1.5	100	0.007	0.002	0.92	10	12	53.13	1.54	1.95	287.15	38.23	0.98
**RR1310-D11-10**	AMP	Laupapa	8.270	177.063	**52.98**	**1.36**	1.8	98	0.039	0.008	0.59	8	9	54.41	2.03	2.37	27.44	39.03	0.32
**RR1310-D11-10**	**ALL**	Laupapa	8.270	177.063	**52.86**	**0.77**	1.41	99	0.0067	0.0012	0.73	18	21	53.15	1.08	1.61	286.24	32.01	0.72
**RR1310-D13-01**	GM	Tayasa	8.622	177.911									35						
**RR1310-D13-01**	PLG	Tayasa	8.622	177.911	**50.52**	**0.2**	1.15	92	0.019	0.001	2.63	18	24	50.35	0.37	1.19	321.42	44.81	2.58
**RR1310-D14-01**	GM	Nukufetau	8.008	178.370	**43.64**	**0.57**	1.13	34	0.05	0.005	0.58	20	36	43.54	0.96	1.37	296.88	7.8	0.62
RR1310-D14-08	*GM*	*Nukufetau*	8.008	178.370	***48.53***	***0.17***	*1.1*	*51*	*0.119*	*0.011*	*0.95*	*24*	*36*	*48.54*	*0.27*	*1.12*	***342.34***	***3.99***	*1.13*
**RR1310-D14-08**	PLG	Nukufetau	8.008	178.370	**49.82**	**0.18**	1.13	71	0.018	0.001	1.85	15	24	49.74	0.26	1.14	300.54	11.12	1.87

Sample Information					Plateau									Inverse Isochron					
**Sample Name**	Material	Seamount	Lat °S	Long °E	**Age Ma**	**±2σ(i) Ma**	±2σ(f) Ma	^{39}Ar %	K/Ca	± 2σ	MSWD	n	N	Age Ma	±2σ(i) Ma	±2σ(f) Ma	$^{40}Ar/^{36}Ar$ intercept	± 2σ	MSWD
**RR1310-D15-02**	GM	Vaitupu	7.475	178.690	**49.03**	**0.19**	1.12	65	0.07	0.006	1.42	22	36	48.8	0.23	1.12	358.85	38.5	0.94
**RR1310-D15-12**	GM	Vaitupu	7.475	178.690									36						
RR1310-D15-12	*GM*	*Vaitupu*	7.475	178.690	***49.58***	***0.18***	*1.13*	*41*	*0.115*	*0.007*	*0.7*	*19*	*34*	*49.58*	*0.2*	*1.13*	***360.69***	***11.38***	*0.76*
**RR1310-D16-35**	PLG	Telematua	7.473	179.186	**46.63**	**0.49**	1.16	65	0.025	0.001	0.26	11	24	46.98	1.16	1.57	232.51	163.86	0.24
**RR1310-D18-07**	AMP	Funafuti	8.514	179.115	**48.88**	**0.12**	1.1	95	0.066	0.001	1.08	14	16	48.81	0.26	1.12	308.32	37.18	1.1
**RR1310-D18-23**	AMP	Funafuti	8.514	179.115	**48.93**	**0.12**	1.1	86	0.066	0.001	1.44	8	13	49.02	0.23	1.12	248.01	95.56	1.49
**RR1310-D22-29**	GM	Silaga	10.186	179.706	**46.09**	**0.28**	1.07	34	0.083	0.007	4.27	12	31	46.06	0.4	1.11	304.46	52.53	4.67
RR1310-D24-04	*GM*	*Kosciusko*	10.481	179.556	***47.37***	***0.11***	*1.07*	*62*	*0.322*	*0.009*	*2.18*	*24*	*36*	*47.38*	*0.12*	*1.07*	***499.36***	***13.45***	*2.35*
RR1310-D24-11	*GM*	*Kosciusko*	10.481	179.556	***48.16***	***0.19***	*1.1*	*66*	*0.389*	*0.042*	*0.71*	*27*	*36*	*48.46*	*0.29*	*1.12*	***386.16***	***14.07***	*13.89*
**RR1310-D27-35**	AMP	East Niulakita	10.788	179.472	**42.24**	**0.82**	1.26	97	0.003	0.001	0.78	14	17	42.07	1.41	1.7	319.52	106.01	0.79
**RR1310-D27-64**	AMP	East Niulakita	10.788	179.472	**45.15**	**0.12**	1.02	96	0.099	0	0.48	11	19	45.14	0.14	1.02	301.07	45.1	0.53
**RR1310-D27-64**	PLG	East Niulakita	10.788	179.472	**45.73**	**0.14**	1.04	17	0.068	0.001	1.33	9	21	45.73	0.33	1.08	293.06	86.1	1.5

All age are normalized to the FCT-2 sanidine age of 28.201 Ma[41].
Italic samples indicate the plateau values are recalculated using the inverse isochron intercept for $^{40}Ar/^{36}Ar$.
Latitude and Longitude values provided are for the inferred seamount center and not dredge location.
GM = groundmass; PLG = plagioclase; AMP = amphibole; CPX = clinopyroxene; ALL = stacked plateau.
MSWD = Mean square of weighted deviates.
n = number of heating steps used in age calculation; N = total number of heating steps in the experiement.

Supplementary Table 2: Seamount locations and Pb isotopic ranges

Sample Information						
**Sample Name**	Seamount	Latitude °S	Longitude °E	Minimum $^{206}Pb/^{204}Pb$	Maximum $^{206}Pb/^{204}Pb$	N
**RR1310-D02**	Manu Lele Vai	3.906	175.630	19.8	19.8	1
**RR1310-D03**	Taring Nui	4.669	176.567	20.6	20.6	1
**RR1310-D04**	Logotau	5.610	175.692	20.4	20.4	1
**RR1310-D07**	Tefolaha	5.968	176.960	20.9	20.9	1
**RR1310-D10**	Nui	7.697	177.263	20.1	21.1	2
**RR1310-D11**	Laupapa	8.270	177.063	21.1	21.1	1
**RR1310-D13**	Tayasa	8.622	177.911	21.1	21.1	1
**RR1310-D14**	Nukufetau	8.008	178.370	20.8	20.8	1
**RR1310-D15**	Vaitupu	7.475	178.690	20.1	20.2	2
**RR1310-D16**	Telematua	7.473	179.186	20.2	20.2	1
**RR1310-D18**	Funafuti	8.514	179.115	20.9	21.0	2
**RR1310-D22**	Silaga	10.186	179.706	19.3	19.3	1
**RR1310-D24**	Kosciusko	10.481	179.556	21.1	21.3	2
**RR1310-D27**	East Niulakita	10.788	179.472	18.8	21.7	3

Locations represent the inferred center of the seamount.
Lead isotopic analyses were collected on whole rock separates using a MC-ICP-MS following methods outlined in Konter and Storm [42].
N = number of Pb isotopic analyses per seamount.

Supplementary Table 3: Age determinations for seamounts used in hotspot track reconstructions

Louisville							
**Sample**	**Latitude**	**Longitude**	**Age (Ma)**	**2 σ**	**Seamount**	**Source**	**Method**
MTHN-7D1	-50.44	-139.15	1.113	0.042	LOU-2	Koppers, et al. [24]	$^{40}Ar/^{39}Ar$
MTHN-6D1	-48.20	-148.80	13.2	2	LOU-1	Koppers, et al. [24]	$^{40}Ar/^{39}Ar$
AMAT 33D-1	-46.22	-155.93	21.7	0.3	155.9°W (Rumyantsev)	Koppers, et al. [39]	$^{40}Ar/^{39}Ar$
AMAT 31D-5	-45.47	-157.74	24.6	0.3	157.7°W	Koppers, et al. [39]	$^{40}Ar/^{39}Ar$
AMAT 28D-1	-44.28	-159.82	25.6	0.2	159.8°W	Koppers, et al. [39]	$^{40}Ar/^{39}Ar$
AMAT 30D-8	-44.84	-158.47	26.3	0.3	158.5°W	Koppers, et al. [39]	$^{40}Ar/^{39}Ar$
AMAT 27D-1	-44.00	-160.66	29.3	0.3	160.7°W	Koppers, et al. [39]	$^{40}Ar/^{39}Ar$
AMAT 26D-9	-43.55	-161.41	32.2	0.3	161.5°W	Koppers, et al. [39]	$^{40}Ar/^{39}Ar$
AMAT 24D-6	-41.88	-163.70	34.7	0.5	163.6°W	Koppers, et al. [39]	$^{40}Ar/^{39}Ar$
VG-3a/MSN110-1	-41.43	-164.26	36.5	0.4	LOU-3	Koppers, et al. [24]	$^{40}Ar/^{39}Ar$
AMAT 22D-3	-40.74	-165.40	39.6	0.8	165.4°W	Koppers, et al. [39]	$^{40}Ar/^{39}Ar$
AMAT 20D-15B	-40.47	-165.70	41	0.5	165.7°W	Koppers, et al. [39]	$^{40}Ar/^{39}Ar$
AMAT 17D-1	-39.89	-166.10	41.3	0.3	166.1°W	Koppers, et al. [39]	$^{40}Ar/^{39}Ar$
AMAT 16D-1	-39.62	-166.69	43.3	0.4	166.6°W	Koppers, et al. [39]	$^{40}Ar/^{39}Ar$
AMAT 14D-9	-39.15	-167.43	44.7	0.4	167.4°W	Koppers, et al. [39]	$^{40}Ar/^{39}Ar$
AMAT 15D-1a	-39.48	-167.25	45.1	0.3	167.3°W	Koppers, et al. [39]	$^{40}Ar/^{39}Ar$
VM36-03	-38.44	-167.93	45.5	0.8	LOU-9	Koppers, et al. [24]	$^{40}Ar/^{39}Ar$
AMAT 10D-4	-38.16	-168.64	50.2	0.5	168.6°W	Koppers, et al. [39]	$^{40}Ar/^{39}Ar$
AMAT 7D-1	-37.97	-168.27	50.9	0.5	168.3°W	Koppers, et al. [39]	$^{40}Ar/^{39}Ar$
330-U1376A-23R-3, 33-37	-32.24	-171.89	64.2	0.5	Burton Guyot	Koppers, et al. [40]	$^{40}Ar/^{39}Ar$
SOTW-9-52-1	-27.47	-174.41	69	0.6	LOU-7	Koppers, et al. [24]	$^{40}Ar/^{39}Ar$
AMAT 1D-3	-27.60	-174.22	70.9	0.4	27.6°S (Volcano 33)	Koppers, et al. [39]	$^{40}Ar/^{39}Ar$
330-U1374A-63R-3, 99-106	-28.58	-173.29	71.2	0.9	Rigil Guyot	Koppers, et al. [40]	$^{40}Ar/^{39}Ar$
330-U1372A-38R-3, 103-106	-26.67	-174.60	74.3	0.5	Canopus Guyot	Koppers, et al. [40]	$^{40}Ar/^{39}Ar$
SOTW-9-58-7	-25.97	-175.02	78.9	1.3	LOU-4	Koppers, et al. [24]	$^{40}Ar/^{39}Ar$

Hawaii-Emperor							
**Sample**	**Latitude**	**Longitude**	**Age (Ma)**	**2 σ**	**Seamount**	**Source**	**Method**
	19.55	-155.52	0.68	0.08	Hawai'i	Sharp and Renne [29]	$^{40}Ar/^{39}Ar$
	20.553	-156.60	1.02	0.18	Kahoolawe	Naughton, et al. [31]	K/Ar
	20.82	-156.91	1.28	0.04	Lanai	Bonhommet, et al. [43]	K/Ar
	20.77	-156.28	1.32	0.04	West Maui	McDougall [30]	K/Ar
	21.14	-157.01	1.9	0.06	Molokai	Naughton, et al. [31]	K/Ar
	21.47	-157.98	2.6	0.1	Oahu	McDougall [30]	K/Ar
	22.078	-159.52	5.10	0.2	Kauai	McDougall [33]	K/Ar
	21.91	-160.15	7.2	0.3	Nihoa	Dalrymple, et al. [32]	K/Ar
	23.50	-164.50	10.3	0.4	Necker	Dalrymple, et al. [32]	K/Ar
	23.75	-166.21	12	0.4	La Pérouse Pinnacle	Dalrymple, et al. [32]	K/Ar
	25.79	-171.74	19.9	0.3	Laysan	Dalrymple, et al. [34]	$^{40}Ar/^{39}Ar$
04M0247	27.87	-175.85	24.9	0.3	Pearl & Hermes	O'Connor, et al. [35]	$^{40}Ar/^{39}Ar$
06MY290	28.23	-177.37	27.8	0.9	Midway	O'Connor, et al. [35]	$^{40}Ar/^{39}Ar$
06MY076	28.91	-178.61	29.5	0.7	#63 (Clague 1996)	O'Connor, et al. [35]	$^{40}Ar/^{39}Ar$
	28.81	-178.89	31.2	0.2	Unnamed (postshield)	Sharp and Clague [36]	$^{40}Ar/^{39}Ar$
06MY289	28.90	-179.56	32.2	0.9	Helsley	O'Connor, et al. [35]	$^{40}Ar/^{39}Ar$
	31.02	175.90	39.1	0.2	Colohan	Sharp and Clague [36]	$^{40}Ar/^{39}Ar$
04M0216	31.81	174.30	41.7	0.7	Abbot	O'Connor, et al. [35]	$^{40}Ar/^{39}Ar$
	31.80	174.30	41.8	0.3	Abbott (shield)	Sharp and Clague [36]	$^{40}Ar/^{39}Ar$
04M0134	32.28	172.85	44.3	0.7	North Kammu	O'Connor, et al. [35]	$^{40}Ar/^{39}Ar$
04M0165	32.61	172.30	47.7	0.5	Yuryaku	O'Connor, et al. [35]	$^{40}Ar/^{39}Ar$
04M0215	32.08	172.29	47.8	0.7	Daikakuji	O'Connor, et al. [35]	$^{40}Ar/^{39}Ar$
	33.63	171.34	48.2	0.2	Kimmei (postshield)	Sharp and Clague [36]	$^{40}Ar/^{39}Ar$
	35.28	171.77	52.9	0.8	Koko N. (shield)	Sharp and Clague [36]	$^{40}Ar/^{39}Ar$
	37.98	170.41	55.2	0.7	Ojin	Dalrymple and Garcia [37]	K/Ar
	38.071	170.88	55.40	0.9	Jingu	Dalrymple and Garcia [37]	K/Ar
	41.23	170.56	56.41	0.6	Nintoku	Duncan and Keller [38]	$^{40}Ar/^{39}Ar$
	44.73	170.13	61.3	0.3	Suiko (shield)	Sharp and Clague [36]	$^{40}Ar/^{39}Ar$
	51.14	167.36	77.79	1.4	Detroit	Duncan and Keller [38]	$^{40}Ar/^{39}Ar$

Rurutu							
**Sample**	**Latitude**	**Longitude**	**Age (Ma)**	**2 σ**	**Seamount**	**Source**	**Method**
DR07	-23.440	-150.730	0.23	0.004	Arago Seamount	Bonneville, et al. [11]	K/Ar (Cassignol technique)
DT79-RUR-91	-22.478	-151.341	1.16	0.005	Rurutu	Rose [17]	$^{40}Ar/^{39}Ar$
	-20.160	-157.341	6.06	0.36	Mauke	Turner and Jarrard [18]	K/Ar
DT77-AIT-36	-18.858	-159.785	9.53	0.08	Aitutaki	Rose [17]	$^{40}Ar/^{39}Ar$
	-19.994	-158.119	10.34	1.24	Atiu	Turner and Jarrard [18]	K/Ar
RR1310-D27-64	-10.788	179.472	45.73	0.14	East Niulakita	This Study	$^{40}Ar/^{39}Ar$
RR1310-D24-11	-10.481	179.556	48.16	0.19	Kosciusko	This Study	$^{40}Ar/^{39}Ar$
RR1310-D18-23	-8.514	179.115	48.93	0.12	Funafuti	This Study	$^{40}Ar/^{39}Ar$
RR1310-D16-35	-7.473	179.186	46.63	0.49	Telematua	This Study	$^{40}Ar/^{39}Ar$
RR1310-D15-12	-7.475	178.690	49.58	0.18	Vaitupu	This Study	$^{40}Ar/^{39}Ar$
RR1310-D14-08	-8.008	178.370	49.82	0.18	Nukufetau	This Study	$^{40}Ar/^{39}Ar$
RR1310-D13-01	-8.622	177.911	50.52	0.2	Tayasa	This Study	$^{40}Ar/^{39}Ar$
RR1310-D11-10	-8.270	177.063	52.98	1.36	Laupapa	This Study	$^{40}Ar/^{39}Ar$
RR1310-D07-22b	-5.968	176.960	54.95	0.14	Tefolaha	This Study	$^{40}Ar/^{39}Ar$
RR1310-D03-23	-4.669	176.567	61.57	0.5	Taring Nui	This Study	$^{40}Ar/^{39}Ar$
RR1310-D04-1d	-5.610	175.692	63.66	0.34	Logotau	This Study	$^{40}Ar/^{39}Ar$
AVON2-14-7	-0.869	175.514	63.7	0.5	Palutu	Koppers, et al. [6]	$^{40}Ar/^{39}Ar$
AVON2-17-28	-1.436	174.890	65.6	0.5	Kautu	Koppers, et al. [6]	$^{40}Ar/^{39}Ar$
AVON2-16-22	-1.247	175.895	65.8	0.7	Beru	Koppers, et al. [6]	$^{40}Ar/^{39}Ar$
AVON2-5-5	2.503	172.907	69.5	1.1	Musina	Koppers, et al. [6]	$^{40}Ar/^{39}Ar$
AVON2-7-2	0.733	173.251	70.4	0.4	Tofe Tolu	Koppers, et al. [6]	$^{40}Ar/^{39}Ar$
AVON2-1-7	4.721	172.471	70.9	0.5	Niu	Koppers, et al. [6]	$^{40}Ar/^{39}Ar$
AVON2-4-6	3.157	172.814	72.4	0.5	Burtaritari	Koppers, et al. [6]	$^{40}Ar/^{39}Ar$

All $^{40}Ar/^{39}Ar$ ages are normalized to a FCTs of 28.201 Ma[41] and the decay constant of Min, et al. [44].
Latitude and Longitude represents the inferred seamount center.

Supplementary Note 2

$^{40}Ar/^{39}Ar$ Age Results

RR1310-D02-04 > Groundmass > KONRAD (13-INT-08)
TUVALU > RURUTU HOTSPOT
15-OSU-04 (4A22-15) > Incremental Heating > Kevin Konrad

Information on Analysis and Constants Used in Calculations

Project = **KONRAD (13-INT-08)**
Sample = **RR1310-D02-04**
Material = **Groundmass**
Location = **Rurutu Hotspot**
Region = **Tuvalu**
Analyst = **Kevin Konrad**
Irradiation = **15-OSU-04 (4A22-15)**
Position = **X: 0 | Y: 0 | Z/H: 30.74 mm**
FCT-NM Age = **28.201 ± 0.023 Ma**
FCT-NM Reference = **Kuiper et al (2008)**
FCT-NM 40Ar/39Ar Ratio = **9.00786 ± 0.01468**
FCT-NM J-value = **0.00174485 ± 0.00000284**
Air Shot 40Ar/36Ar = **303.8360 ± 0.7869**
Air Shot MDF = **0.99313217 ± 0.00085470 (LIN)**
Experiment Type = **Incremental Heating**
Extraction Method = **Bulk Laser Heating**
Heating = **77 sec**
Isolation = **3.00 min**
Instrument = **ARGUS-VI-D**
Preferred Age = **Plateau Age**
Age Classification = **Unknown**
IGSN = Undefined
Rock Class = Undefined
Lithology = **Basalt**
Lat-Lon = Undefined - Undefined
Age Equations = **Min et al. (2000)**
Negative Intensities = **Allowed**
Collector Calibrations = **36Ar**
Decay 40K = **5.530 ± 0.048 E-10 1/a**
Decay 39Ar = **2.940 ± 0.016 E-07 1/h**
Decay 37Ar = **8.230 ± 0.012 E-04 1/h**
Decay 36Cl = **2.257 ± 0.015 E-06 1/a**
Decay 40K(EC,β^+) = **0.580 ± 0.009 E-10 1/a**
Decay 40K(β^-) = **4.950 ± 0.043 E-10 1/a**
Atmospheric 40/36(a) = **295.50**
Atmospheric 38/36(a) = **0.1869**
Production 39/37(ca) = **0.0006756 ± 0.0000089**
Production 38/37(ca) = **0.0000718 ± 0.0000092**
Production 36/37(ca) = **0.0002663 ± 0.0000004**
Production 40/39(k) = **0.003823 ± 0.000102**
Production 38/39(k) = **0.012031 ± 0.000019**
Production 36/38(cl) = **262.80 ± 1.71**
Scaling Ratio K/Ca = **0.430**
Abundance Ratio 40K/K = **1.1700 ± 0.0100 E-04**
Atomic Weight K = **39.0983 ± 0.0001 g**

Results

Results	40(a)/36(a) ± 2σ		40(r)/39(k) ± 2σ		Age ± 2σ (Ma)		MSWD	39Ar(k) (%,n)	K/Ca ± 2σ
**Age Plateau**			24.12892	± 0.04641	**74.57**	**± 0.28**	3.18	29.78	0.406 ± 0.052
Error Mean				± 0.19%		**± 0.37%**	0%	10	
				Full External Error		± 1.69	1.94	2σ Confidence Limit	
				Analytical Error		± 0.14	1.7831	Error Magnification	
**Total Fusion Age**			22.91108	± 0.01505	**70.88**	**± 0.23**		43	0.098 ± 0.000
				± 0.07%		**± 0.33%**			
				Full External Error		± 1.60			
				Analytical Error		± 0.05			
**Normal Isochron**	**416.88**	**± 163.98**	24.02966	± 0.14117	**74.27**	**± 0.49**	2.60	29.78	
Error Chron		**± 39.33%**		± 0.59%		**± 0.66%**	1%	10	
				Full External Error		± 1.73	2.00	2σ Confidence Limit	
				Analytical Error		± 0.43	1.6132	Error Magnification	
**Inverse Isochron**	**445.12**	**± 144.89**	24.00666	± 0.13741	**74.20**	**± 0.48**	2.45	29.78	
Error Chron		**± 32.55%**		± 0.57%		**± 0.65%**	1%	10	
				Full External Error		± 1.73	2.00	2σ Confidence Limit	
				Analytical Error		± 0.42	1.5653	Error Magnification	
							2%	Spreading Factor	

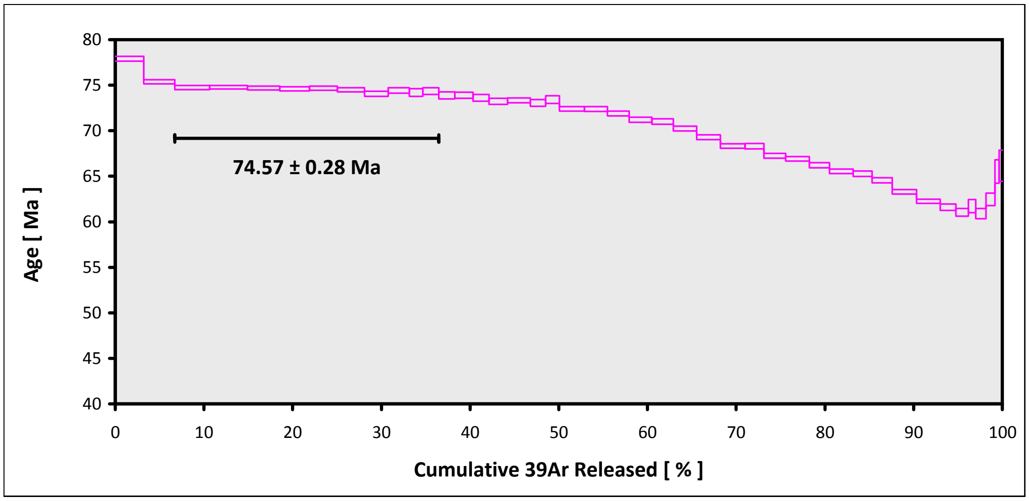

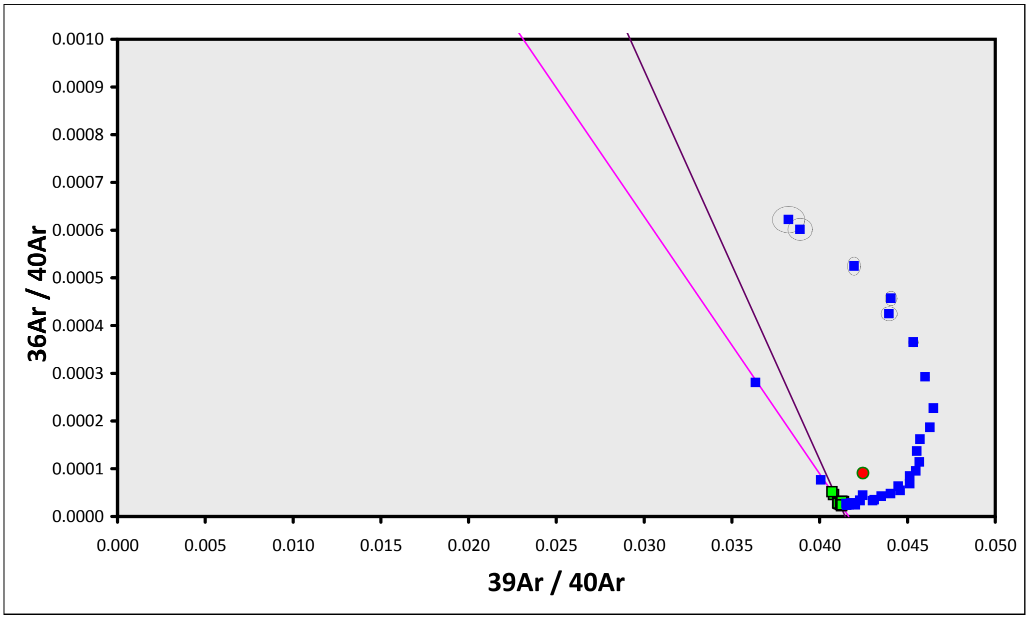

RR1310-D02-05 > Groundmass > RURUTU (13-INT-08) TUVALU > RURUTU HOTSPOT 14-OSU-02 (2F9-14) > Incremental Heating > Kevin Konrad

Information on Analysis and Constants Used in Calculations

Project = **RURUTU (13-INT-08)**
Sample = **RR1310-D02-05**
Material = **Groundmass**
Location = **Rurutu Hotspot**
Region = **Tuvalu**
Analyst = **Kevin Konrad**
Irradiation = **14-OSU-02 (2F9-14)**
Position = **X: 0 | Y: 0 | Z/H: 24.1 mm**
FCT-NM Age = **28.201 ± 0.023 Ma**
FCT-NM Reference = **Kuiper et al. (2008)**
FCT-NM 40Ar/39Ar Ratio = **8.79064 ± 0.01002**
FCT-NM J-value = **0.00178797 ± 0.00000204**
Air Shot 40Ar/36Ar = **303.8990 ± 0.4163**
Air Shot MDF = **0.99308170 ± 0.00066632 (LIN)**
Experiment Type = **Incremental Heating**
Extraction Method = **Bulk Laser Heating**
Heating = **77 sec**
Isolation = **10.00 min**
Instrument = **ARGUS-VI-D**
Preferred Age = Undefined
Age Classification = Undefined
IGSN = Undefined
Rock Class = Undefined
Lithology = **Basalt**
Lat-Lon = Undefined - Undefined
Age Equations = **Min et al. (2000)**
Negative Intensities = **Allowed**
Collector Calibrations = **40Ar 36Ar**
Decay 40K = **5.530 ± 0.048 E-10 1/a**
Decay 39Ar = **2.940 ± 0.016 E-07 1/h**
Decay 37Ar = **8.230 ± 0.012 E-04 1/h**
Decay 36Cl = **2.257 ± 0.015 E-06 1/a**
Decay 40K(EC,β^+) = **0.580 ± 0.009 E-10 1/a**
Decay 40K(β^-) = **4.950 ± 0.043 E-10 1/a**
Atmospheric 40/36(a) = **295.50**
Atmospheric 38/36(a) = **0.1869**
Production 39/37(ca) = **0.0006756 ± 0.0000089**
Production 38/37(ca) = **0.0000718 ± 0.0000092**
Production 36/37(ca) = **0.0002663 ± 0.0000004**
Production 40/39(k) = **0.003823 ± 0.000102**
Production 38/39(k) = **0.012031 ± 0.000019**
Production 36/38(cl) = **262.80 ± 1.71**
Scaling Ratio K/Ca = **0.430**
Abundance Ratio 40K/K = **1.1700 ± 0.0100 E-04**
Atomic Weight K = **39.0983 ± 0.0001 g**

Results

Results	40(a)/36(a) ± 2σ	40(r)/39(k) ± 2σ	Age ± 2σ (Ma)	MSWD	39Ar(k) (%,n)	K/Ca ± 2σ
**Age Plateau**<br>Cannot Calculate						
**Total Fusion Age**		21.41028 ± 0.03140<br>± 0.15%	**67.93 ± 0.18**<br>**± 0.27%**<br>Full External Error ± 1.53<br>Analytical Error ± 0.10		36	0.186 ± 0.007
**Normal Isochron**<br>Cannot Calculate						
**Inverse Isochron**<br>Cannot Calculate						

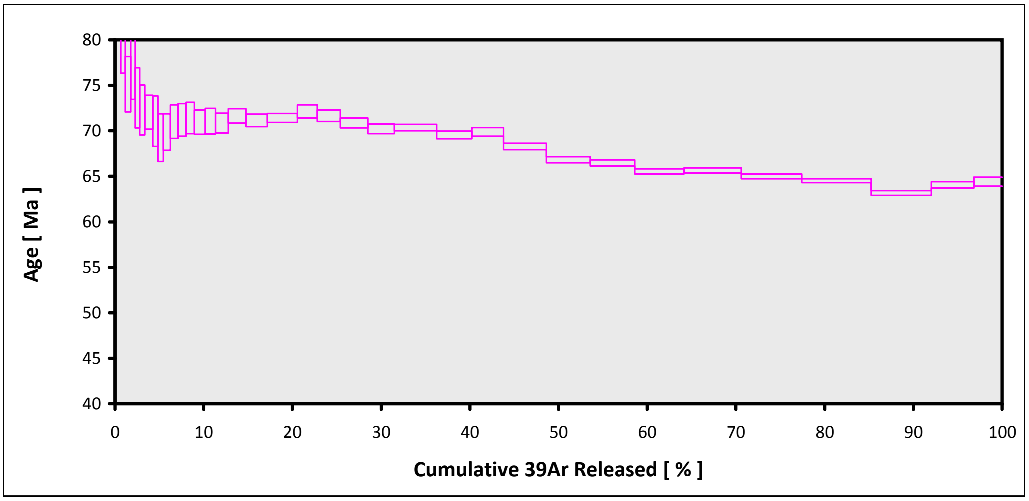

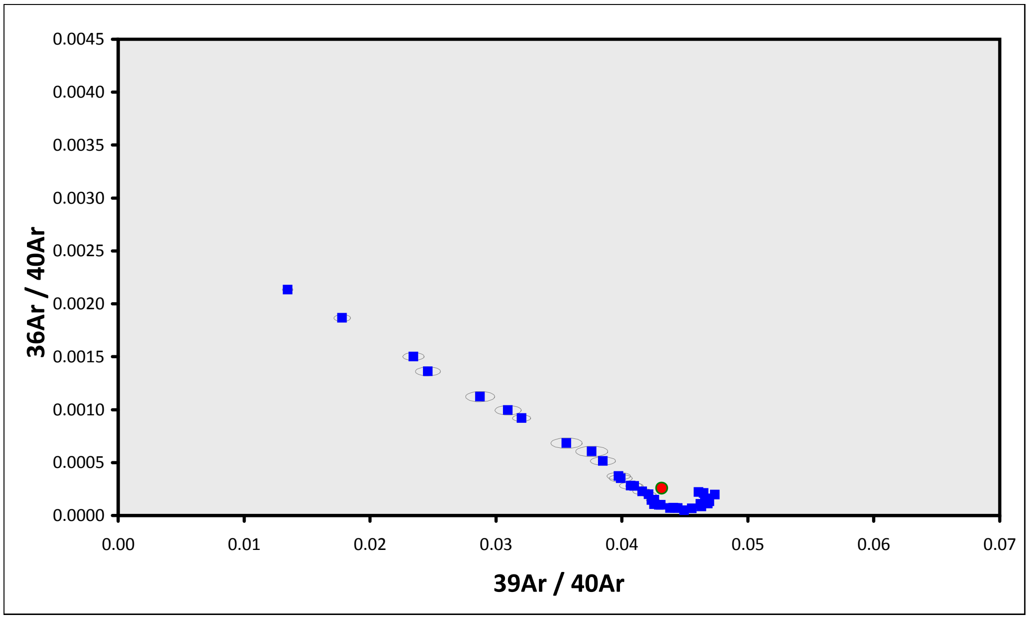

RR1310-D02-17 > Groundmass > KONRAD (13-INT-08)
TUVALU > RURUTU HOTSPOT
15-OSU-04 (4A23-15) > Incremental Heating > Kevin Konrad

Information on Analysis and Constants Used in Calculations

Project = **KONRAD (13-INT-08)**
Sample = **RR1310-D02-17**
Material = **Groundmass**
Location = **Rurutu Hotspot**
Region = **Tuvalu**
Analyst = **Kevin Konrad**
Irradiation = **15-OSU-04 (4A23-15)**
Position = **X: 0 | Y: 0 | Z/H: 32.65 mm**
FCT-NM Age = **28.201 ± 0.023 Ma**
FCT-NM Reference = **Kuiper et al (2008)**
FCT-NM 40Ar/39Ar Ratio = **9.04632 ± 0.01466**
FCT-NM J-value = **0.00173743 ± 0.00000281**
Air Shot 40Ar/36Ar = **303.8380 ± 0.7930**
Air Shot MDF = **0.99313057 ± 0.00085829 (LIN)**
Experiment Type = **Incremental Heating**
Extraction Method = **Bulk Laser Heating**
Heating = **77 sec**
Isolation = **3.00 min**
Instrument = **ARGUS-VI-D**
Preferred Age = Undefined
Age Classification = Undefined
IGSN = Undefined
Rock Class = Undefined
Lithology = **Basalt**
Lat-Lon = Undefined - Undefined
Age Equations = **Min et al. (2000)**
Negative Intensities = **Allowed**
Collector Calibrations = **36Ar**
Decay 40K = **5.530 ± 0.048 E-10 1/a**
Decay 39Ar = **2.940 ± 0.016 E-07 1/h**
Decay 37Ar = **8.230 ± 0.012 E-04 1/h**
Decay 36Cl = **2.257 ± 0.015 E-06 1/a**
Decay 40K(EC,β^+) = **0.580 ± 0.009 E-10 1/a**
Decay 40K(β^-) = **4.950 ± 0.043 E-10 1/a**
Atmospheric 40/36(a) = **295.50**
Atmospheric 38/36(a) = **0.1869**
Production 39/37(ca) = **0.0006756 ± 0.0000089**
Production 38/37(ca) = **0.0000718 ± 0.0000092**
Production 36/37(ca) = **0.0002663 ± 0.0000004**
Production 40/39(k) = **0.003823 ± 0.000102**
Production 38/39(k) = **0.012031 ± 0.000019**
Production 36/38(cl) = **262.80 ± 1.71**
Scaling Ratio K/Ca = **0.430**
Abundance Ratio 40K/K = **1.1700 ± 0.0100 E-04**
Atomic Weight K = **39.0983 ± 0.0001 g**

Results

Results	40(a)/36(a) ± 2σ	40(r)/39(k) ± 2σ	Age ± 2σ (Ma)	MSWD	39Ar(k) (%,n)	K/Ca ± 2σ
**Age Plateau**<br>Cannot Calculate						
**Total Fusion Age**		19.98583 ± 0.01379<br>± 0.07%	**61.73 ± 0.20**<br>**± 0.33%**<br>Full External Error ± 1.40<br>Analytical Error ± 0.04		43	0.117 ± 0.000
**Normal Isochron**<br>Cannot Calculate						
**Inverse Isochron**<br>Cannot Calculate						

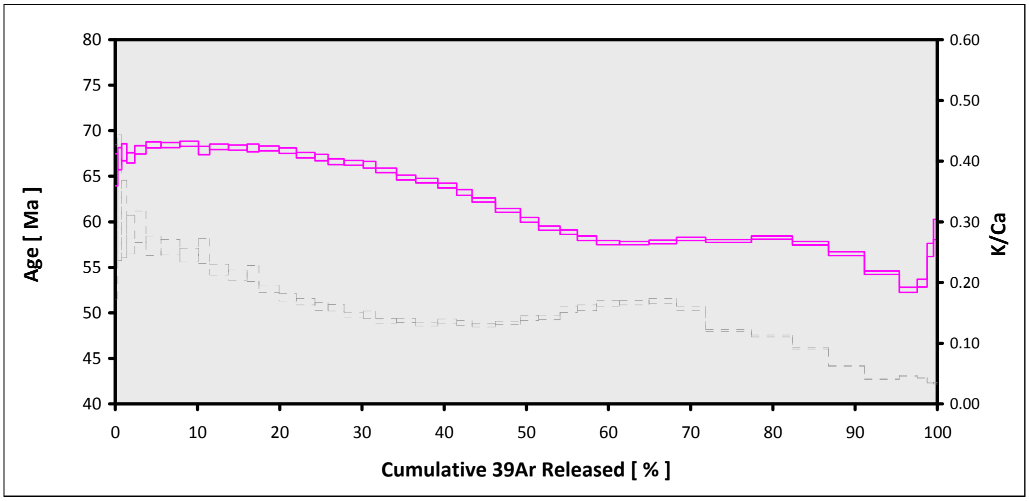

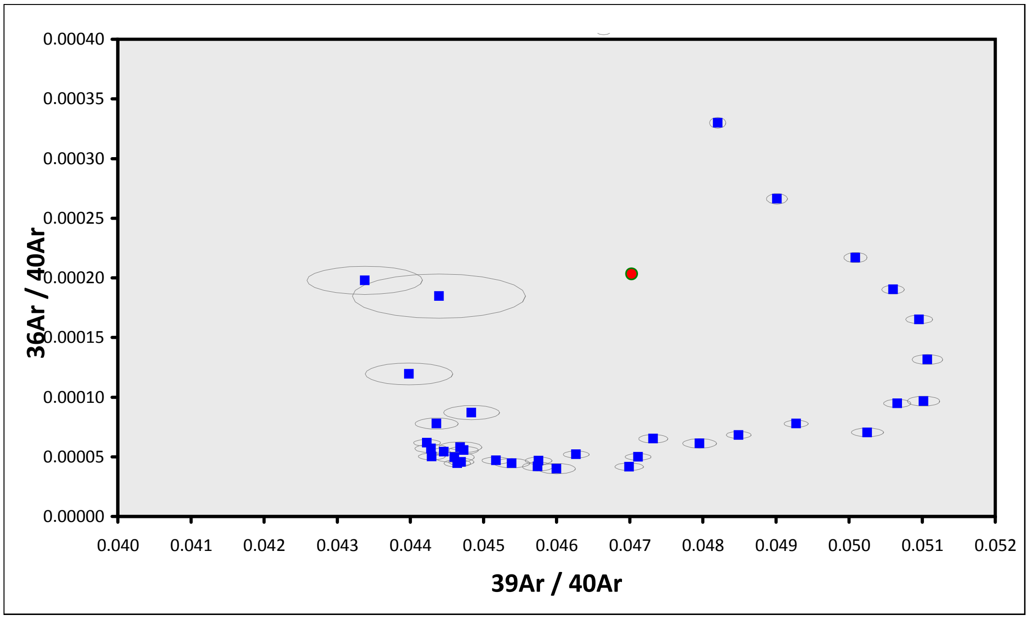

RR1310-D03-23 > Groundmass > RURUTU (13-INT-08)
TUVALU > RURUTU HOTSPOT
14-OSU-02 (2A35-14) > Incremental Heating > Kevin Konrad

Information on Analysis and Constants Used in Calculations

Project = **RURUTU (13-INT-08)**
Sample = **RR1310-D03-23**
Material = **Groundmass**
Location = **Rurutu Hotspot**
Region = **Tuvalu**
Analyst = **Kevin Konrad**
Irradiation = **14-OSU-02 (2A35-14)**
Position = **X: 0 | Y: 0 | Z/H: 41 mm**
FCT-NM Age = **28.201 ± 0.023 Ma**
FCT-NM Reference = **Kuiper et al. (2008)**
FCT-NM 40Ar/39Ar Ratio = **8.87612 ± 0.00843**
FCT-NM J-value = **0.00177075 ± 0.00000168**
Air Shot 40Ar/36Ar = **295.5000 ± 0.2955**
Air Shot MDF = **1.00000000 ± 0.00064391 (LIN)**
Experiment Type = **Incremental Heating**
Extraction Method = **Bulk Laser Heating**
Heating = **77 sec**
Isolation = **10.00 min**
Instrument = **ARGUS-VI-D**
Preferred Age = Undefined
Age Classification = Undefined
IGSN = Undefined
Rock Class = Undefined
Lithology = **Basalt**
Lat-Lon = Undefined - Undefined
Age Equations = **Min et al. (2000)**
Negative Intensities = **Allowed**
Collector Calibrations = **40Ar 36Ar**
Decay 40K = **5.530 ± 0.048 E-10 1/a**
Decay 39Ar = **2.940 ± 0.016 E-07 1/h**
Decay 37Ar = **8.230 ± 0.012 E-04 1/h**
Decay 36Cl = **2.257 ± 0.015 E-06 1/a**
Decay 40K(EC,β^+) = **0.580 ± 0.009 E-10 1/a**
Decay 40K(β^-) = **4.950 ± 0.043 E-10 1/a**
Atmospheric 40/36(a) = **295.50**
Atmospheric 38/36(a) = **0.1869**
Production 39/37(ca) = **0.0006756 ± 0.0000089**
Production 38/37(ca) = **0.0000718 ± 0.0000092**
Production 36/37(ca) = **0.0002663 ± 0.0000004**
Production 40/39(k) = **0.003823 ± 0.000102**
Production 38/39(k) = **0.012031 ± 0.000019**
Production 36/38(cl) = **262.80 ± 1.71**
Scaling Ratio K/Ca = **0.430**
Abundance Ratio 40K/K = **1.1700 ± 0.0100 E-04**
Atomic Weight K = **39.0983 ± 0.0001 g**

Results

Results	40(a)/36(a) ± 2σ	40(r)/39(k) ± 2σ	Age ± 2σ (Ma)	MSWD	39Ar(k) (%,n)	K/Ca ± 2σ
**Age Plateau**						
Cannot Calculate						
**Total Fusion Age**		30.54538 ± 0.12925 ± 0.42%	**95.26 ± 0.43 ± 0.45%**		37	0.0417 ± 0.0012
			Full External Error ± 2.17			
			Analytical Error ± 0.39			
**Normal Isochron**						
Cannot Calculate						
**Inverse Isochron**						
Cannot Calculate						

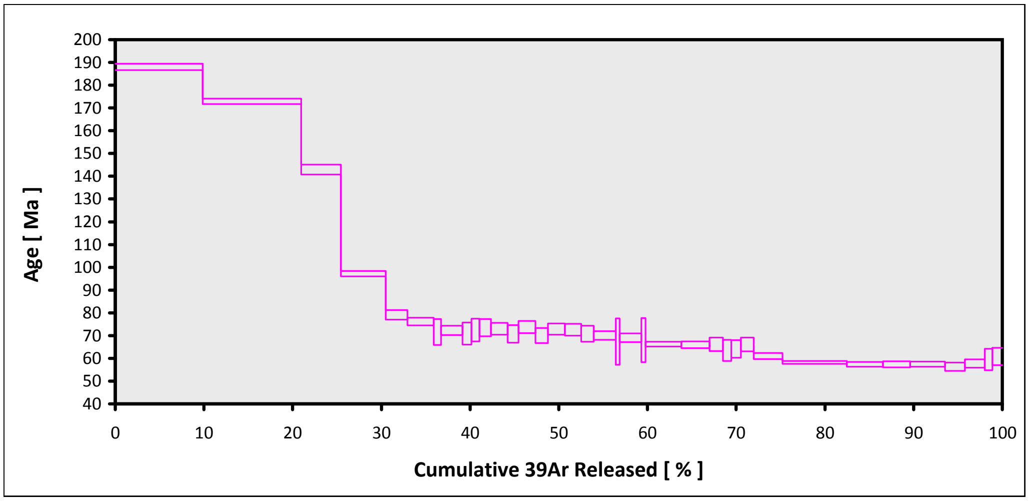

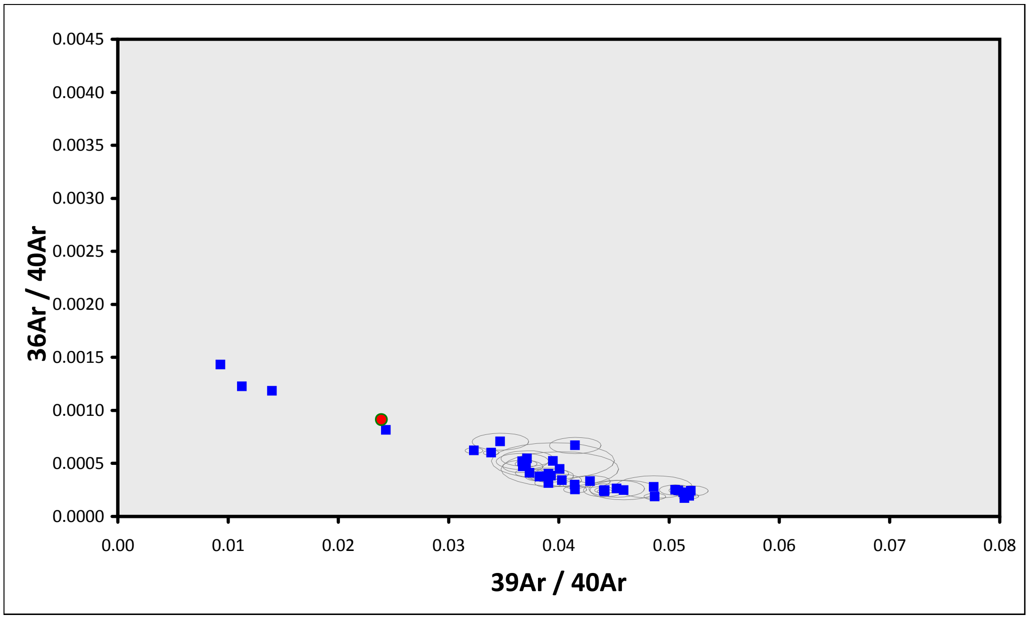

RR1310-D03-23 > Groundmass > KONRAD (13-INT-08)
TUVALU > RURUTU HOTSPOT
15-OSU-04 (4A26-15) > Incremental Heating > Kevin Konrad

Information on Analysis and Constants Used in Calculations

Project = **KONRAD (13-INT-08)**
Sample = **RR1310-D03-23**
Material = **Groundmass**
Location = **Rurutu Hotspot**
Region = **Tuvalu**
Analyst = **Kevin Konrad**
Irradiation = **15-OSU-04 (4A26-15)**
Position = **X: 0 | Y: 0 | Z/H: 35.82 mm**
FCT-NM Age = **28.201 ± 0.023 Ma**
FCT-NM Reference = **Kuiper et al (2008)**
FCT-NM 40Ar/39Ar Ratio = **9.11395 ± 0.01467**
FCT-NM J-value = **0.00172454 ± 0.00000278**
Air Shot 40Ar/36Ar = **303.3750 ± 0.5036**
Air Shot MDF = **0.99350212 ± 0.00070551 (LIN)**
Experiment Type = **Incremental Heating**
Extraction Method = **Bulk Laser Heating**
Heating = **77 sec**
Isolation = **3.00 min**
Instrument = **ARGUS-VI-D**
Preferred Age = **Plateau Age**
Age Classification = **Eruption Age**
IGSN = Undefined
Rock Class = Undefined
Lithology = **Basalt**
Lat-Lon = Undefined - Undefined
Age Equations = **Min et al. (2000)**
Negative Intensities = **Allowed**
Collector Calibrations = **36Ar**
Decay 40K = **5.530 ± 0.048 E-10 1/a**
Decay 39Ar = **2.940 ± 0.016 E-07 1/h**
Decay 37Ar = **8.230 ± 0.012 E-04 1/h**
Decay 36Cl = **2.257 ± 0.015 E-06 1/a**
Decay 40K(EC,β^+) = **0.580 ± 0.009 E-10 1/a**
Decay 40K(β^-) = **4.950 ± 0.043 E-10 1/a**
Atmospheric 40/36(a) = **295.50**
Atmospheric 38/36(a) = **0.1869**
Production 39/37(ca) = **0.0006756 ± 0.0000089**
Production 38/37(ca) = **0.0000718 ± 0.0000092**
Production 36/37(ca) = **0.0002663 ± 0.0000004**
Production 40/39(k) = **0.003823 ± 0.000102**
Production 38/39(k) = **0.012031 ± 0.000019**
Production 36/38(cl) = **262.80 ± 1.71**
Scaling Ratio K/Ca = **0.430**
Abundance Ratio 40K/K = **1.1700 ± 0.0100 E-04**
Atomic Weight K = **39.0983 ± 0.0001 g**

Results

Results	40(a)/36(a) ± 2σ		40(r)/39(k) ± 2σ		Age ± 2σ (Ma)		MSWD	39Ar(k) (%,n)	K/Ca ± 2σ
**Age Plateau**			20.08333	± 0.15406<br>± 0.77%	**61.57**	**± 0.50**<br>**± 0.82%**	0.75<br>73%	40.27<br>16	0.0570 ± 0.0060
				Full External Error		± 1.47	1.73	2σ Confidence Limit	
				Analytical Error		± 0.46	1.0000	Error Magnification	
**Total Fusion Age**			19.60116	± 0.10119<br>± 0.52%	**60.12**	**± 0.36**<br>**± 0.60%**		37	0.0185 ± 0.0001
				Full External Error		± 1.39			
				Analytical Error		± 0.31			
**Normal Isochron**	**246.67**	**± 129.56**<br>**± 52.52%**	20.19070	± 0.32931<br>± 1.63%	**61.89**	**± 1.01**<br>**± 1.63%**	0.76<br>71%	40.27<br>16	
				Full External Error		± 1.72	1.76	2σ Confidence Limit	
				Analytical Error		± 0.99	1.0000	Error Magnification	
**Inverse Isochron**	**256.78**	**± 100.82**<br>**± 39.26%**	20.17881	± 0.32526<br>± 1.61%	**61.86**	**± 1.00**<br>**± 1.62%**	0.77<br>70%	40.27<br>16	
				Full External Error		± 1.71	1.76	2σ Confidence Limit	
				Analytical Error		± 0.98	1.0000	Error Magnification	
							7%	Spreading Factor	

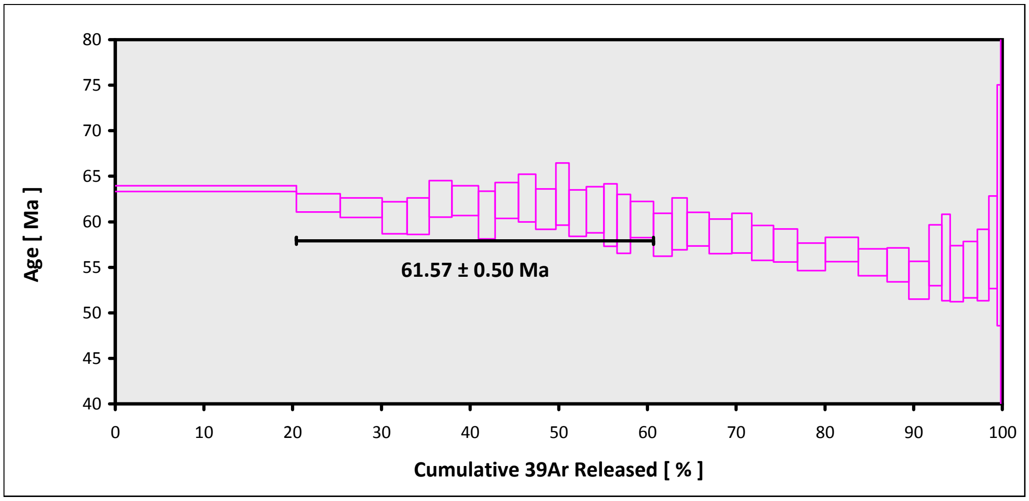

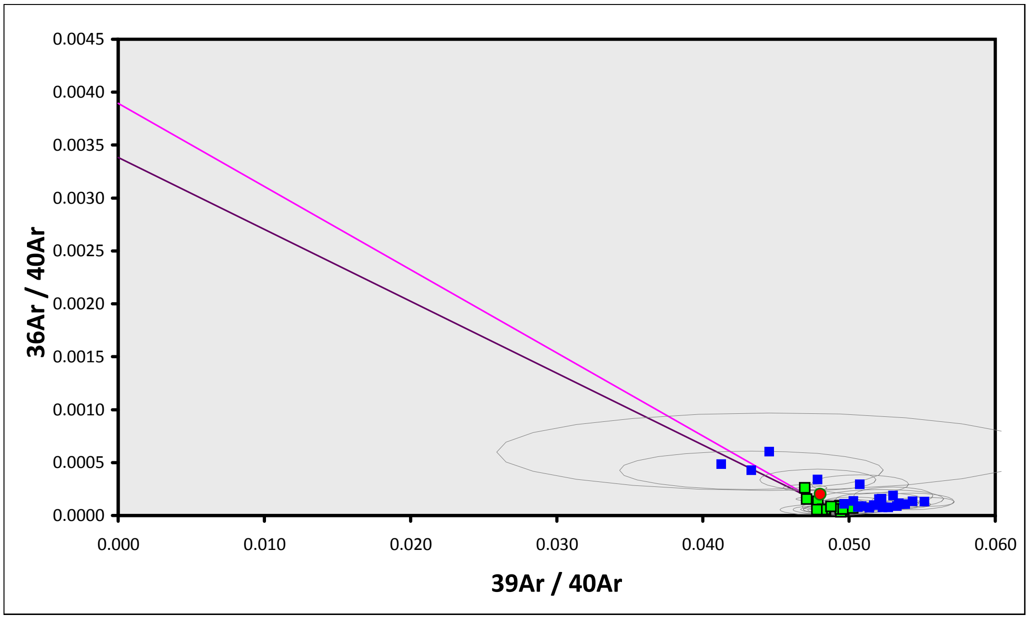

RR1310-D04-1D > Plagioclase > RURUTU (13-INT-08) TUVALU > RURUTU HOTSPOT 14-OSU-02 (2A2-14) > Incremental Heating > Kevin Konrad

Information on Analysis and Constants Used in Calculations

Project = **RURUTU (13-INT-08)**
Sample = **RR1310-D04-1D**
Material = **Plagioclase**
Location = **Rurutu Hotspot**
Region = **Tuvalu**
Analyst = **Kevin Konrad**
Irradiation = **14-OSU-02 (2A2-14)**
Position = **X: 0 | Y: 0 | Z/H: 3.8 mm**
FCT-NM Age = **28.201 ± 0.023 Ma**
FCT-NM Reference = **Kuiper et al. (2008)**
FCT-NM 40Ar/39Ar Ratio = **8.89235 ± 0.00836**
FCT-NM J-value = **0.00176752 ± 0.00000166**
Air Shot 40Ar/36Ar = **303.8790 ± 0.4315**
Air Shot MDF = **0.99309772 ± 0.00067253 (LIN)**
Experiment Type = **Incremental Heating**
Extraction Method = **Bulk Laser Heating**
Heating = **77 sec**
Isolation = **6.00 min**
Instrument = **ARGUS-VI-D**
Preferred Age = **Plateau Age**
Age Classification = **Eruption Age**
IGSN = Undefined
Rock Class = Undefined
Lithology = **Basalt**
Lat-Lon = Undefined - Undefined
Age Equations = **Min et al. (2000)**
Negative Intensities = **Allowed**
Collector Calibrations = **40Ar 36Ar**
Decay 40K = **5.530 ± 0.048 E-10 1/a**
Decay 39Ar = **2.940 ± 0.016 E-07 1/h**
Decay 37Ar = **8.230 ± 0.012 E-04 1/h**
Decay 36Cl = **2.257 ± 0.015 E-06 1/a**
Decay 40K(EC,β$^+$) = **0.580 ± 0.009 E-10 1/a**
Decay 40K(β$^-$) = **4.950 ± 0.043 E-10 1/a**
Atmospheric 40/36(a) = **295.50**
Atmospheric 38/36(a) = **0.1869**
Production 39/37(ca) = **0.0006756 ± 0.0000089**
Production 38/37(ca) = **0.0000718 ± 0.0000092**
Production 36/37(ca) = **0.0002663 ± 0.0000004**
Production 40/39(k) = **0.003823 ± 0.000102**
Production 38/39(k) = **0.012031 ± 0.000019**
Production 36/38(cl) = **262.80 ± 1.71**
Scaling Ratio K/Ca = **0.430**
Abundance Ratio 40K/K = **1.1700 ± 0.0100 E-04**
Atomic Weight K = **39.0983 ± 0.0001 g**

Results

Results	40(a)/36(a) ± 2σ		40(r)/39(k) ± 2σ		Age ± 2σ (Ma)		MSWD	39Ar(k) (%,n)	K/Ca ± 2σ
**Age Plateau**			20.27335	± 0.10198<br>± 0.50%	**63.66**	**± 0.34<br>± 0.53%**	1.28<br>24%	51.52<br>10	0.0128 ± 0.0004
				Full External Error	± 1.46		1.94	2σ Confidence Limit	
				Analytical Error	± 0.31		1.1306	Error Magnification	
**Total Fusion Age**			20.07903	± 0.07430<br>± 0.37%	**63.06**	**± 0.26<br>± 0.41%**		26	0.0136 ± 0.0001
				Full External Error	± 1.43				
				Analytical Error	± 0.23				
**Normal Isochron**<br>**No Convergence**	**284.46**	**± 67.94<br>± 23.88%**	20.29294	± 0.26851<br>± 1.32%	**63.72**	**± 0.84<br>± 1.31%**	1.33<br>22%	51.52<br>10	
				Full External Error	± 1.65		2.00	2σ Confidence Limit	
				Analytical Error	± 0.83		1.1519	Error Magnification	
**Inverse Isochron**	**268.88**	**± 65.82<br>± 24.48%**	20.37287	± 0.26993<br>± 1.32%	**63.97**	**± 0.84<br>± 1.31%**	1.35<br>21%	51.52<br>10	
				Full External Error	± 1.66		2.00	2σ Confidence Limit	
				Analytical Error	± 0.83		1.1605	Error Magnification	
							6%	Spreading Factor	

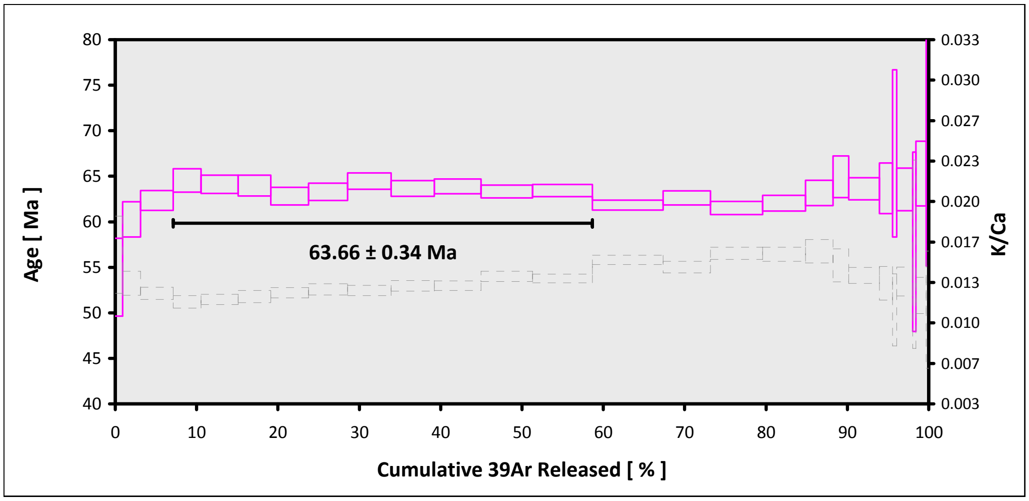

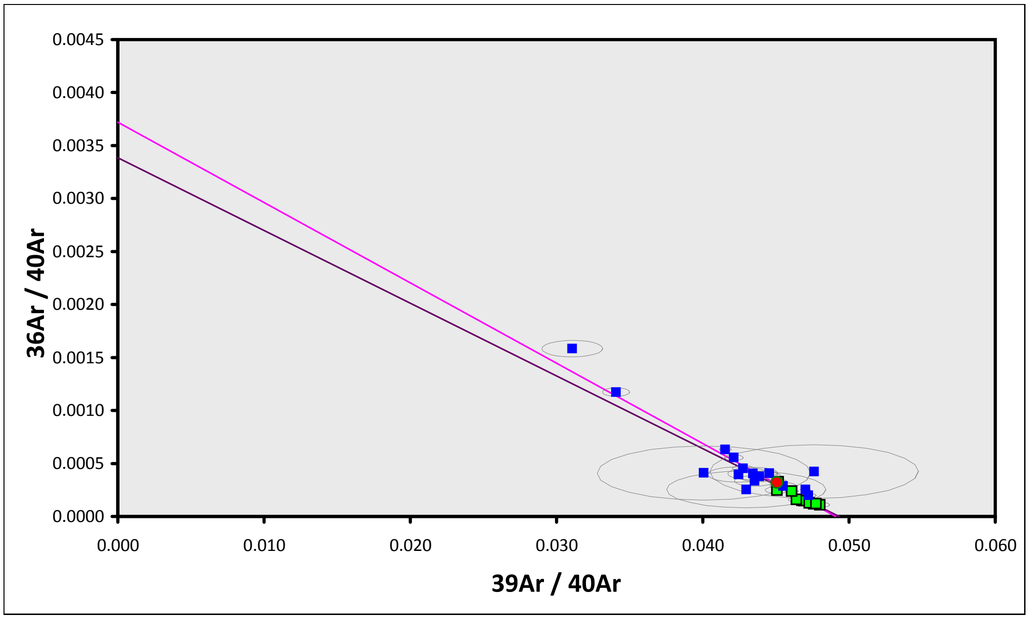

RR1310-D07-09 > Clinopyroxene > RURUTU (13-INT-08) TUVALU > RURUTU HOTSPOT 14-OSU-06 (6A34-14) > Incremental Heating > Kevin Konrad

Information on Analysis and Constants Used in Calculations

Project = **RURUTU (13-INT-08)**
Sample = **RR1310-D07-09**
Material = **Clinopyroxene**
Location = **Rurutu Hotspot**
Region = **Tuvalu**
Analyst = **Kevin Konrad**
Irradiation = **14-OSU-06 (6A34-14)**
Position = **X: 0 | Y: 0 | Z/H: 53.24 mm**
FCT-NM Age = **28.201 ± 0.023 Ma**
FCT-NM Reference = **Kuiper et al (2008)**
FCT-NM 40Ar/39Ar Ratio = **9.14509 ± 0.00969**
FCT-NM J-value = **0.00171867 ± 0.00000182**
Air Shot 40Ar/36Ar = **303.3230 ± 0.5035**
Air Shot MDF = **0.99354392 ± 0.00070564 (LIN)**
Experiment Type = **Incremental Heating**
Extraction Method = **Bulk Laser Heating**
Heating = **77 sec**
Isolation = **6.00 min**
Instrument = **ARGUS-VI-D**
Preferred Age = **Plateau Age**
Age Classification = **Eruption Age**
IGSN = Undefined
Rock Class = Undefined
Lithology = **Basalt**
Lat-Lon = Undefined - Undefined
Age Equations = **Min et al. (2000)**
Negative Intensities = **Allowed**
Collector Calibrations = **40Ar 36Ar**
Decay 40K = **5.530 ± 0.048 E-10 1/a**
Decay 39Ar = **2.940 ± 0.016 E-07 1/h**
Decay 37Ar = **8.230 ± 0.012 E-04 1/h**
Decay 36Cl = **2.257 ± 0.015 E-06 1/a**
Decay 40K(EC,β^+) = **0.580 ± 0.009 E-10 1/a**
Decay 40K(β^-) = **4.950 ± 0.043 E-10 1/a**
Atmospheric 40/36(a) = **295.50**
Atmospheric 38/36(a) = **0.1869**
Production 39/37(ca) = **0.0006756 ± 0.0000089**
Production 38/37(ca) = **0.0000718 ± 0.0000092**
Production 36/37(ca) = **0.0002663 ± 0.0000004**
Production 40/39(k) = **0.003823 ± 0.000102**
Production 38/39(k) = **0.012031 ± 0.000019**
Production 36/38(cl) = **262.80 ± 1.71**
Scaling Ratio K/Ca = **0.430**
Abundance Ratio 40K/K = **1.1700 ± 0.0100 E-04**
Atomic Weight K = **39.0983 ± 0.0001 g**

Results

Results	40(a)/36(a) ± 2σ		40(r)/39(k) ± 2σ		Age ± 2σ (Ma)		MSWD	39Ar(k) (%,n)	K/Ca ± 2σ
Age Plateau			16.06375	± 0.81964<br>± 5.10%	**49.25**	**± 2.48**<br>**± 5.04%**	0.90<br>49%	71.38<br>7	0.070 ± 0.008
				Full External Error	± 2.72		2.15	2σ Confidence Limit	
				Analytical Error	± 2.48		1.0000	Error Magnification	
Total Fusion Age			22.68798	± 1.06820<br>± 4.71%	**69.17**	**± 3.20**<br>**± 4.62%**		14	0.082 ± 0.004
				Full External Error	± 3.55				
				Analytical Error	± 3.20				
Normal Isochron	**297.88**	**± 5.25**<br>**± 1.76%**	15.76395	± 1.10081<br>± 6.98%	**48.34**	**± 3.33**<br>**± 6.89%**	0.95<br>45%	71.38<br>7	
				Full External Error	± 3.50		2.26	2σ Confidence Limit	
				Analytical Error	± 3.33		1.0000	Error Magnification	
Inverse Isochron	**297.60**	**± 5.30**<br>**± 1.78%**	15.86785	± 1.12123<br>± 7.07%	**48.66**	**± 3.39**<br>**± 6.97%**	0.96<br>44%	71.38<br>7	
				Full External Error	± 3.56		2.26	2σ Confidence Limit	
				Analytical Error	± 3.39		1.0000	Error Magnification	
							71%	Spreading Factor	

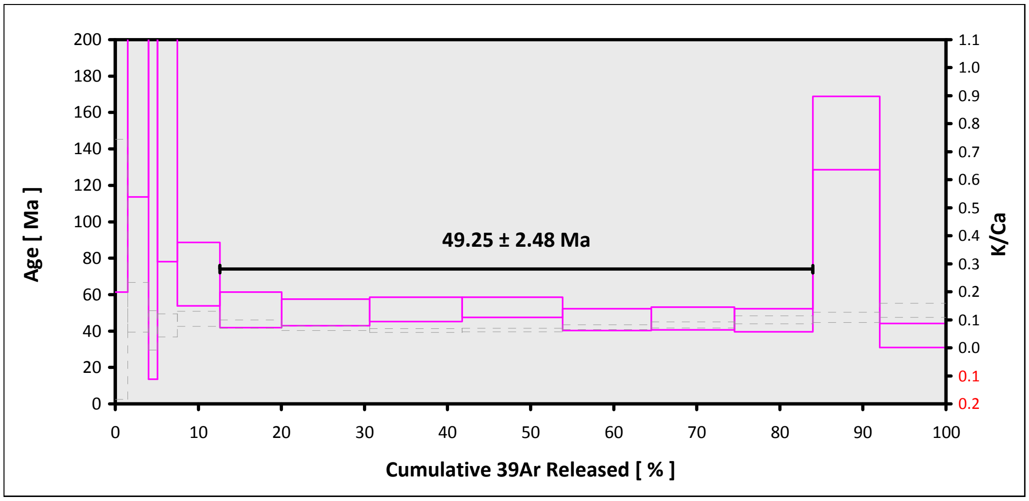

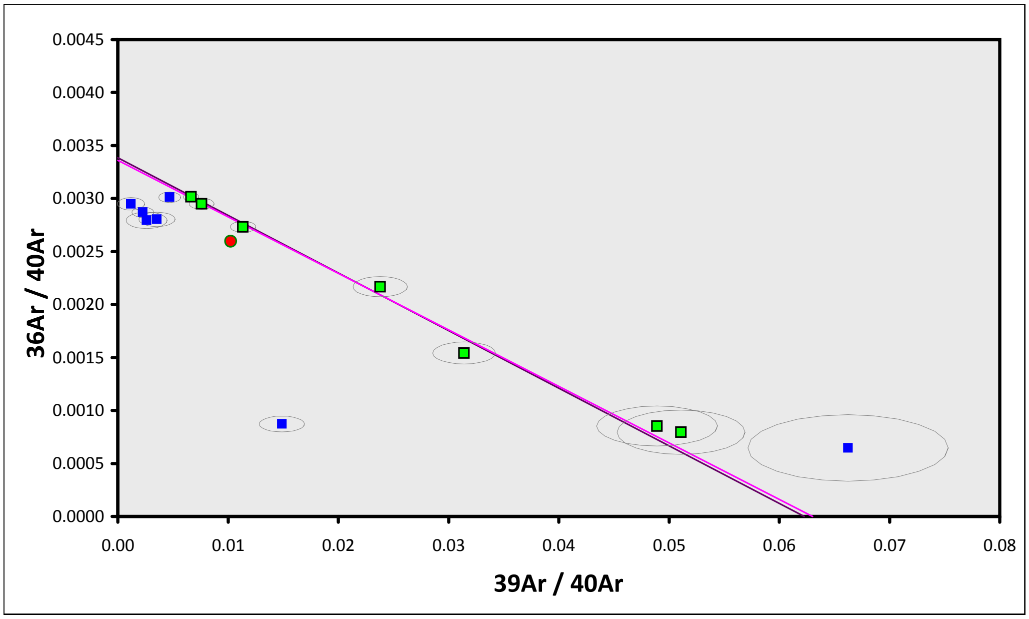

RR1310-D07-09 > Plagioclase > KONRAD (13-INT-08)
TUVALU > RURUTU HOTSPOT
15-OSU-04 (4A16-15) > Incremental Heating > Kevin Konrad

Information on Analysis and Constants Used in Calculations

Project = **KONRAD (13-INT-08)**
Sample = **RR1310-D07-09**
Material = **Plagioclase**
Location = **Rurutu Hotspot**
Region = **Tuvalu**
Analyst = **Kevin Konrad**
Irradiation = **15-OSU-04 (4A16-15)**
Position = **X: 0 | Y: 0 | Z/H: 22.82 mm**
FCT-NM Age = **28.201 ± 0.023 Ma**
FCT-NM Reference = **Kuiper et al (2008)**
FCT-NM 40Ar/39Ar Ratio = **8.86813 ± 0.01454**
FCT-NM J-value = **0.00177235 ± 0.00000291**
Air Shot 40Ar/36Ar = **304.5420 ± 0.5604**
Air Shot MDF = **0.99256779 ± 0.00072877 (LIN)**
Experiment Type = **Incremental Heating**
Extraction Method = **Bulk Laser Heating**
Heating = **77 sec**
Isolation = **1.50 min**
Instrument = **ARGUS-VI-D**
Preferred Age = **Plateau Age**
Age Classification = **Eruption Age**
IGSN = Undefined
Rock Class = Undefined
Lithology = **Basalt**
Lat-Lon = Undefined - Undefined
Age Equations = **Min et al. (2000)**
Negative Intensities = **Allowed**
Collector Calibrations = **36Ar**
Decay 40K = **5.530 ± 0.048 E-10 1/a**
Decay 39Ar = **2.940 ± 0.016 E-07 1/h**
Decay 37Ar = **8.230 ± 0.012 E-04 1/h**
Decay 36Cl = **2.257 ± 0.015 E-06 1/a**
Decay 40K(EC,β^+) = **0.580 ± 0.009 E-10 1/a**
Decay 40K(β^-) = **4.950 ± 0.043 E-10 1/a**
Atmospheric 40/36(a) = **295.50**
Atmospheric 38/36(a) = **0.1869**
Production 39/37(ca) = **0.0006756 ± 0.0000089**
Production 38/37(ca) = **0.0000718 ± 0.0000092**
Production 36/37(ca) = **0.0002663 ± 0.0000004**
Production 40/39(k) = **0.003823 ± 0.000102**
Production 38/39(k) = **0.012031 ± 0.000019**
Production 36/38(cl) = **262.80 ± 1.71**
Scaling Ratio K/Ca = **0.430**
Abundance Ratio 40K/K = **1.1700 ± 0.0100 E-04**
Atomic Weight K = **39.0983 ± 0.0001 g**

Results

Results	40(a)/36(a) ± 2σ		40(r)/39(k) ± 2σ		Age ± 2σ (Ma)			MSWD	39Ar(k) (%,n)	K/Ca ± 2σ
**Age Plateau**			16.14270	± 0.10393	**51.01**	**± 0.36**		3.68	72.52	0.0050 ± 0.0001
Error Mean				± 0.64%		**± 0.71%**		0%	18	
					Full External Error	± 1.20		1.69	2σ Confidence Limit	
					Analytical Error	± 0.32		1.9172	Error Magnification	
**Total Fusion Age**			16.23652	± 0.05083	**51.30**	**± 0.23**			32	0.0051 ± 0.0000
				± 0.31%		**± 0.45%**				
					Full External Error	± 1.17				
					Analytical Error	± 0.16				
**Normal Isochron**	**294.18**	**± 9.59**	16.16191	± 0.23542	**51.07**	**± 0.75**		3.84	72.52	
Error Chron		**± 3.26%**		± 1.46%		**± 1.47%**		0%	18	
					Full External Error	± 1.37		1.71	2σ Confidence Limit	
					Analytical Error	± 0.73		1.9603	Error Magnification	
**Inverse Isochron**	**293.53**	**± 9.53**	16.18964	± 0.23364	**51.16**	**± 0.75**		3.92	72.52	
Error Chron		**± 3.25%**		± 1.44%		**± 1.46%**		0%	18	
					Full External Error	± 1.37		1.71	2σ Confidence Limit	
					Analytical Error	± 0.73		1.9798	Error Magnification	
								40%	Spreading Factor	

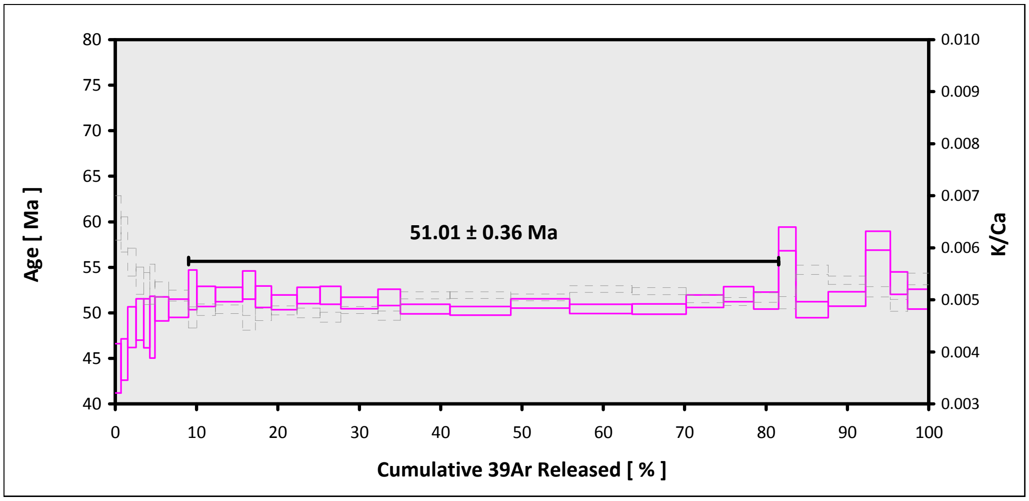

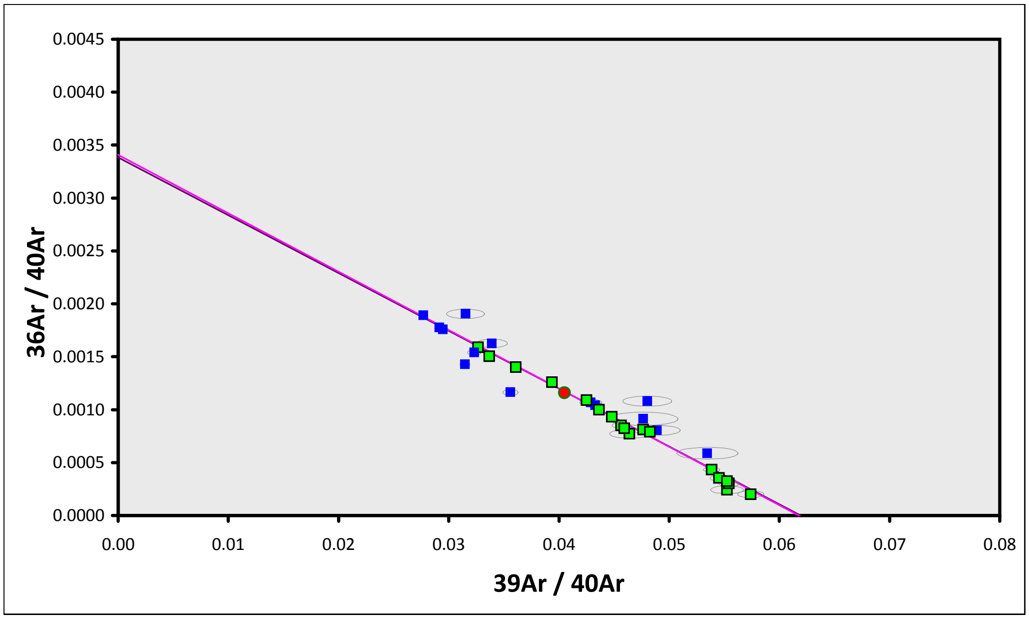

STACK > RR1310-D07-09 > Clinopyroxene > RURUTU (13-INT-08)
TUVALU > RURUTU HOTSPOT
14-OSU-06 (6A34-14) > Incremental Heating > Kevin Konrad

Information on Analysis and Constants Used in Calculations

Project = **RURUTU (13-INT-08)**
Stack = **RR1310-D07-09**
Material = **Clinopyroxene**
Location = **Rurutu Hotspot**
Region = **Tuvalu**
Analyst = **Kevin Konrad**
Irradiation = **14-OSU-06 (6A34-14)**
Position = **X: 0 | Y: 0 | Z/H: 53.24 mm**
FCT-NM Age = **28.201 ± 0.023 Ma**
FCT-NM Reference = **Kuiper et al (2008)**
FCT-NM 40Ar/39Ar Ratio = **9.14509 ± 0.00969**
FCT-NM J-value = **0.00171867 ± 0.00000182**
Air Shot 40Ar/36Ar = **303.3230 ± 0.5035**
Air Shot MDF = **0.99354392 ± 0.00070564 (LIN)**
Experiment Type = **Incremental Heating**
Extraction Method = **Bulk Laser Heating**
Heating = **77 sec**
Isolation = **6.00 min**
Instrument = **ARGUS-VI-D**
Preferred Age = **Plateau Age**
Age Classification = **Eruption Age**
IGSN = Undefined
Rock Class = Undefined
Lithology = **Basalt**
Lat-Lon = Undefined - Undefined
Age Equations = **Min et al. (2000)**
Negative Intensities = **Allowed**
Collector Calibrations = **40Ar 39Ar 38Ar 37Ar 36Ar**
Decay 40K = **5.530 ± 0.048 E-10 1/a**
Decay 39Ar = **2.940 ± 0.016 E-07 1/h**
Decay 37Ar = **8.230 ± 0.012 E-04 1/h**
Decay 36Cl = **2.257 ± 0.015 E-06 1/a**
Decay 40K(EC,β^+) = **0.580 ± 0.009 E-10 1/a**
Decay 40K(β^-) = **4.950 ± 0.043 E-10 1/a**
Atmospheric 40/36(a) = **295.50**
Atmospheric 38/36(a) = **0.1869**
Production 39/37(ca) = **0.0006756 ± 0.0000089**
Production 38/37(ca) = **0.0000718 ± 0.0000092**
Production 36/37(ca) = **0.0002663 ± 0.0000004**
Production 40/39(k) = **0.003823 ± 0.000102**
Production 38/39(k) = **0.012031 ± 0.000019**
Production 36/38(cl) = **262.80 ± 1.71**
Scaling Ratio K/Ca = **0.430**
Abundance Ratio 40K/K = **1.1700 ± 0.0100 E-04**
Atomic Weight K = **39.0983 ± 0.0001 g**

Results

Results	40(a)/36(a) ± 2σ		40(r)/39(k) ± 2σ		Age ± 2σ (Ma)		MSWD	39Ar(k) (%,n)	K/Ca ± 2σ
**Age Plateau**			16.64416	± 0.09519	**51.00**	**± 0.31**	2.91	72.46	0.0050 ± 0.0002
Error Mean				± 0.57%		**± 0.60%**	0%	25	
					Full External Error	± 1.18	1.58	2σ Confidence Limit	
					Analytical Error	± 0.29	1.7067	Error Magnification	
**Total Fusion Age**			17.08265	± 0.07061	**52.33**	**± 0.24**		46	0.0053 ± 0.0000
				± 0.41%		**± 0.46%**			
					Full External Error	± 1.20			
					Analytical Error	± 0.21			
**Normal Isochron**	**294.82**	**± 5.55**	16.65025	± 0.16066	**51.02**	**± 0.50**	2.99	72.46	
Error Chron		**± 1.88%**		± 0.96%		**± 0.97%**	0%	25	
					Full External Error	± 1.25	1.59	2σ Confidence Limit	
					Analytical Error	± 0.49	1.7305	Error Magnification	
**Inverse Isochron**	**294.50**	**± 5.54**	16.67249	± 0.16012	**51.09**	**± 0.50**	3.03	72.46	
Error Chron		**± 1.88%**		± 0.96%		**± 0.97%**	0%	25	
					Full External Error	± 1.25	1.59	2σ Confidence Limit	
					Analytical Error	± 0.48	1.7401	Error Magnification	
							82%	Spreading Factor	

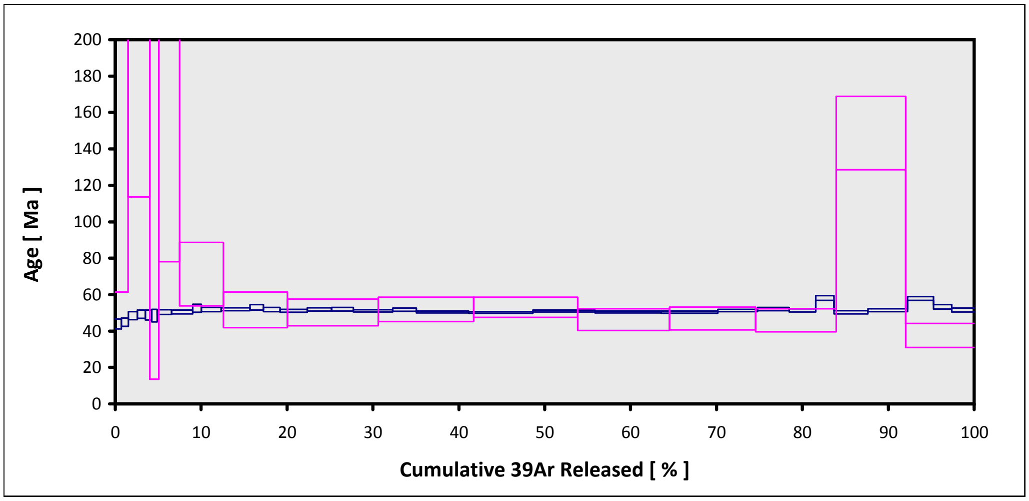

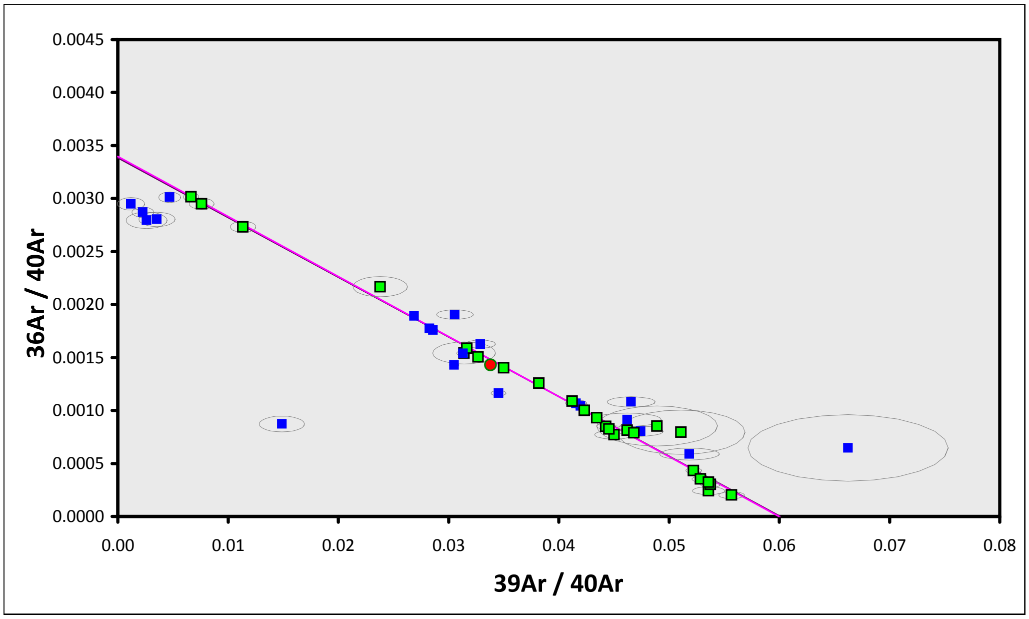

RR1310-D07-22B > Groundmass > RURUTU (13-INT-08)
TUVALU > RURUTU HOTSPOT
14-OSU-02 (2A43-14) > Incremental Heating > Kevin Konrad

Information on Analysis and Constants Used in Calculations

Project = **RURUTU (13-INT-08)**
Sample = **RR1310-D07-22B**
Material = **Groundmass**
Location = **Rurutu Hotspot**
Region = **Tuvalu**
Analyst = **Kevin Konrad**
Irradiation = **14-OSU-02 (2A43-14)**
Position = **X: 0 | Y: 0 | Z/H: 51.8 mm**
FCT-NM Age = **28.201 ± 0.023 Ma**
FCT-NM Reference = **Kuiper et al. (2008)**
FCT-NM 40Ar/39Ar Ratio = **8.97135 ± 0.00843**
FCT-NM J-value = **0.00175195 ± 0.00000165**
Air Shot 40Ar/36Ar = **304.0620 ± 0.4044**
Air Shot MDF = **0.99295122 ± 0.00066114 (LIN)**
Experiment Type = **Incremental Heating**
Extraction Method = **Bulk Laser Heating**
Heating = **77 sec**
Isolation = **10.00 min**
Instrument = **ARGUS-VI-D**
Preferred Age = **Plateau Age**
Age Classification = **Eruption Age**
IGSN = Undefined
Rock Class = Undefined
Lithology = **Basalt**
Lat-Lon = Undefined - Undefined
Age Equations = **Min et al. (2000)**
Negative Intensities = **Allowed**
Collector Calibrations = **40Ar 36Ar**
Decay 40K = **5.530 ± 0.048 E-10 1/a**
Decay 39Ar = **2.940 ± 0.016 E-07 1/h**
Decay 37Ar = **8.230 ± 0.012 E-04 1/h**
Decay 36Cl = **2.257 ± 0.015 E-06 1/a**
Decay 40K(EC,β^+) = **0.580 ± 0.009 E-10 1/a**
Decay 40K(β^-) = **4.950 ± 0.043 E-10 1/a**
Atmospheric 40/36(a) = **295.50**
Atmospheric 38/36(a) = **0.1869**
Production 39/37(ca) = **0.0006756 ± 0.0000089**
Production 38/37(ca) = **0.0000718 ± 0.0000092**
Production 36/37(ca) = **0.0002663 ± 0.0000004**
Production 40/39(k) = **0.003823 ± 0.000102**
Production 38/39(k) = **0.012031 ± 0.000019**
Production 36/38(cl) = **262.80 ± 1.71**
Scaling Ratio K/Ca = **0.430**
Abundance Ratio 40K/K = **1.1700 ± 0.0100 E-04**
Atomic Weight K = **39.0983 ± 0.0001 g**

Results

Results	40(a)/36(a) ± 2σ		40(r)/39(k) ± 2σ		Age ± 2σ (Ma)		MSWD	39Ar(k) (%,n)	K/Ca ± 2σ
**Age Plateau**			17.51506	± 0.01787<br>± 0.10%	**54.65**	**± 0.12**<br>**± 0.21%**	1.42<br>19%	51.12<br>8	0.575 ± 0.033
					Full External Error	± 1.23	2.07	2σ Confidence Limit	
					Analytical Error	± 0.05	1.1920	Error Magnification	
**Total Fusion Age**			17.09669	± 0.01226<br>± 0.07%	**53.37**	**± 0.11**<br>**± 0.20%**		33	0.443 ± 0.012
					Full External Error	± 1.20			
					Analytical Error	± 0.04			
**Normal Isochron**	**492.05**	**± 196.51**<br>**± 39.94%**	17.43163	± 0.08215<br>± 0.47%	**54.40**	**± 0.27**<br>**± 0.50%**	1.22<br>29%	51.12<br>8	
					Full External Error	± 1.25	2.15	2σ Confidence Limit	
					Analytical Error	± 0.25	1.1025	Error Magnification	
**Inverse Isochron**<br>Clustered Points	**419.29**	**± 184.14**<br>**± 43.92%**	17.46577	± 0.08026<br>± 0.46%	**54.50**	**± 0.27**<br>**± 0.49%**	1.27<br>27%	51.12<br>8	
					Full External Error	± 1.25	2.15	2σ Confidence Limit	
					Analytical Error	± 0.25	1.1285	Error Magnification	
							1%	Spreading Factor	

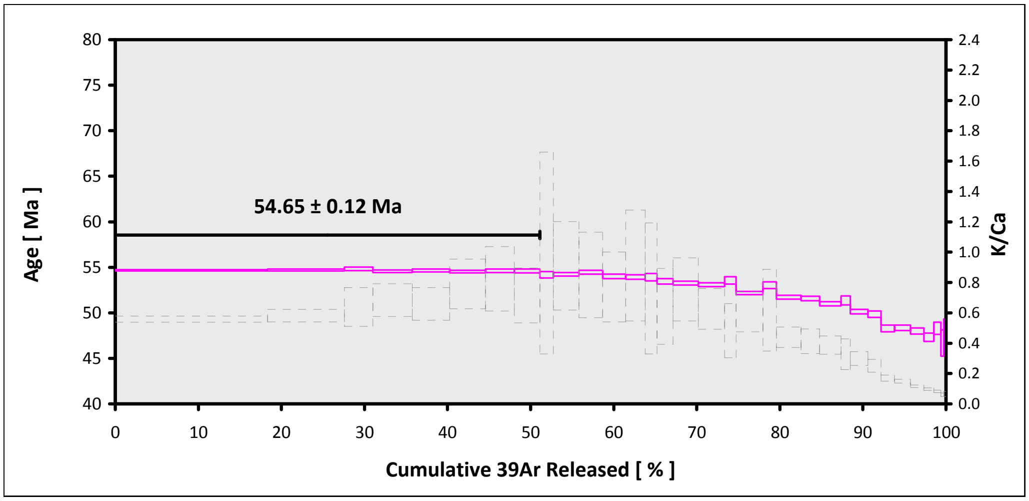

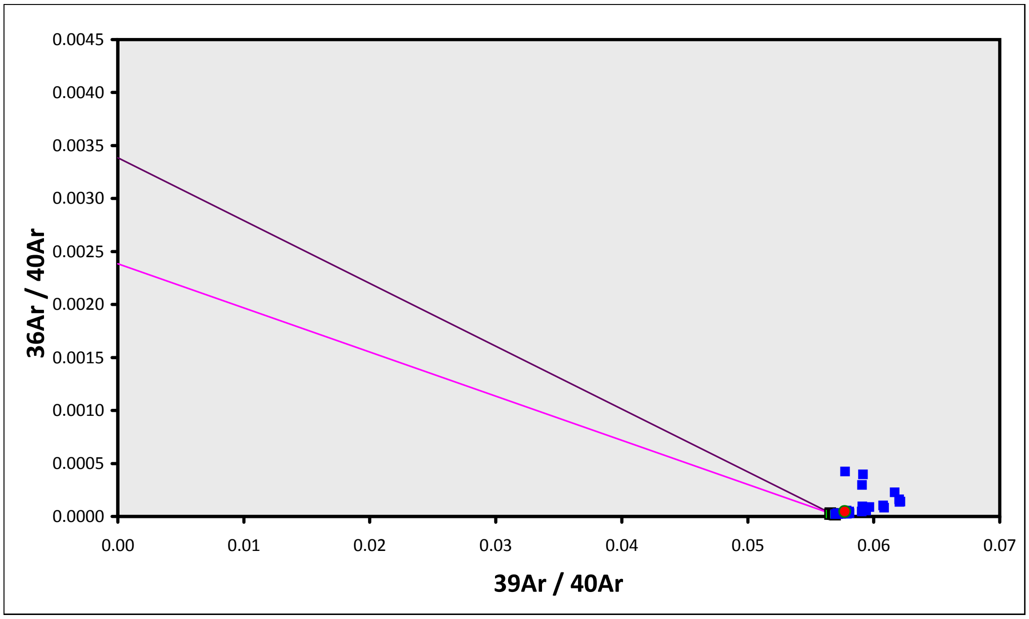

RR1310-D07-22B > Groundmass > RURUTU (13-INT-08)
TUVALU > RURUTU HOTSPOT
14-OSU-02 (2A43-14) > Incremental Heating > Kevin Konrad

Information on Analysis and Constants Used in Calculations

Project = **RURUTU (13-INT-08)**
Sample = **RR1310-D07-22B**
Material = **Groundmass**
Location = **Rurutu Hotspot**
Region = **Tuvalu**
Analyst = **Kevin Konrad**
Irradiation = **14-OSU-02 (2A43-14)**
Position = **X: 0 | Y: 0 | Z/H: 51.8 mm**
FCT-NM Age = **28.201 ± 0.023 Ma**
FCT-NM Reference = **Kuiper et al. (2008)**
FCT-NM 40Ar/39Ar Ratio = **8.97135 ± 0.00843**
FCT-NM J-value = **0.00175195 ± 0.00000165**
Air Shot 40Ar/36Ar = **303.6980 ± 0.4707**
Air Shot MDF = **0.99324280 ± 0.00068974 (LIN)**
Experiment Type = **Incremental Heating**
Extraction Method = **Bulk Laser Heating**
Heating = **77 sec**
Isolation = **10.00 min**
Instrument = **ARGUS-VI-D**
Preferred Age = **Plateau Age**
Age Classification = **Eruption Age**
IGSN = Undefined
Rock Class = Undefined
Lithology = **Basalt**
Lat-Lon = Undefined - Undefined
Age Equations = **Min et al. (2000)**
Negative Intensities = **Allowed**
Collector Calibrations = **40Ar 36Ar**
Decay 40K = **5.530 ± 0.048 E-10 1/a**
Decay 39Ar = **2.940 ± 0.016 E-07 1/h**
Decay 37Ar = **8.230 ± 0.012 E-04 1/h**
Decay 36Cl = **2.257 ± 0.015 E-06 1/a**
Decay 40K(EC,β^+) = **0.580 ± 0.009 E-10 1/a**
Decay 40K(β^-) = **4.950 ± 0.043 E-10 1/a**
Atmospheric 40/36(a) = **295.50**
Atmospheric 38/36(a) = **0.1869**
Production 39/37(ca) = **0.0006756 ± 0.0000089**
Production 38/37(ca) = **0.0000718 ± 0.0000092**
Production 36/37(ca) = **0.0002663 ± 0.0000004**
Production 40/39(k) = **0.003823 ± 0.000102**
Production 38/39(k) = **0.012031 ± 0.000019**
Production 36/38(cl) = **262.80 ± 1.71**
Scaling Ratio K/Ca = **0.430**
Abundance Ratio 40K/K = **1.1700 ± 0.0100 E-04**
Atomic Weight K = **39.0983 ± 0.0001 g**

Results

Results	40(a)/36(a) ± 2σ		40(r)/39(k) ± 2σ		Age ± 2σ (Ma)		MSWD	39Ar(k) (%,n)	K/Ca ± 2σ
**Age Plateau**			17.61223	± 0.03005	**54.95**	**± 0.14**	3.68	39.82	0.516 ± 0.050
Error Mean				± 0.17%		**± 0.25%**	0%	10	
				Full External Error		± 1.24	1.94	2σ Confidence Limit	
				Analytical Error		± 0.09	1.9182	Error Magnification	
**Total Fusion Age**			17.23713	± 0.01098	**53.80**	**± 0.11**		36	0.468 ± 0.024
				± 0.06%		**± 0.20%**			
				Full External Error		± 1.21			
				Analytical Error		± 0.03			
**Normal Isochron**	221.19	± 444.39	17.73517	± 0.10666	**55.33**	**± 0.34**	1.65	39.82	
		########		± 0.60%		**± 0.62%**	11%	10	
				Full External Error		± 1.29	2.00	2σ Confidence Limit	
				Analytical Error		± 0.33	1.2829	Error Magnification	
**Inverse Isochron**	813.47	± 557.04	17.87698	± 0.12546	**55.77**	**± 0.40**	0.61	39.82	
Clustered Points		± 68.48%		± 0.70%		**± 0.72%**	77%	10	
				Full External Error		± 1.31	2.00	2σ Confidence Limit	
				Analytical Error		± 0.39	1.0000	Error Magnification	
							1%	Spreading Factor	

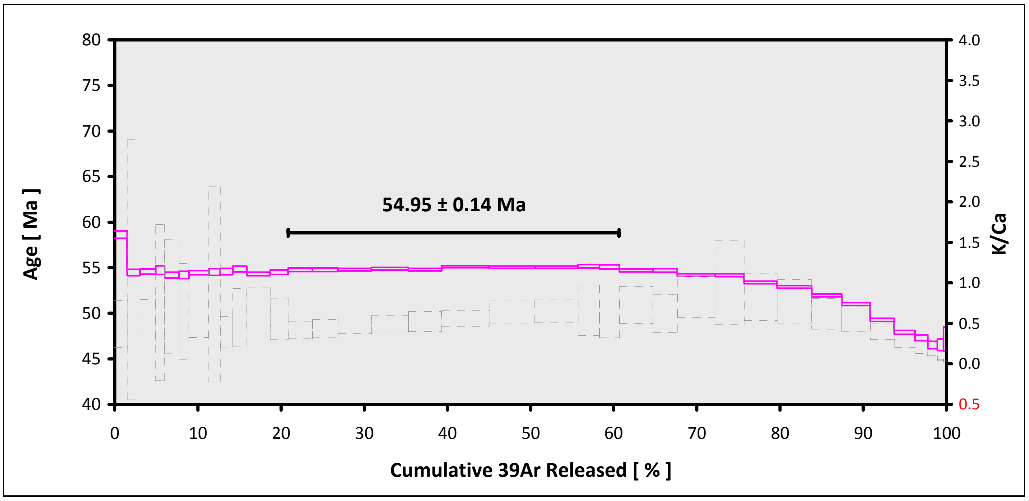

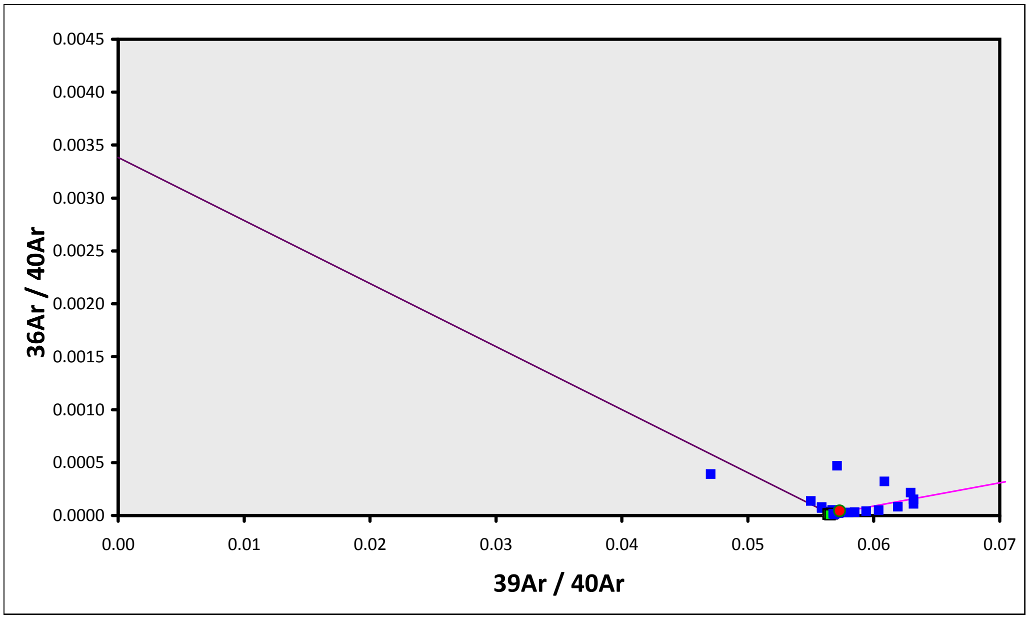

RR1310-D07-22B > Plagioclase > RURUTU (13-INT-08)
TUVALU > RURUTU HOTSPOT
14-OSU-02 (2A23-14) > Incremental Heating > Kevin Konrad

Information on Analysis and Constants Used in Calculations

Project = **RURUTU (13-INT-08)**
Sample = **RR1310-D07-22B**
Material = **Plagioclase**
Location = **Rurutu Hotspot**
Region = **Tuvalu**
Analyst = **Kevin Konrad**
Irradiation = **14-OSU-02 (2A23-14)**
Position = **X: 0 | Y: 0 | Z/H: 27.3 mm**
FCT-NM Age = **28.201 ± 0.023 Ma**
FCT-NM Reference = **Kuiper et al. (2008)**
FCT-NM 40Ar/39Ar Ratio = **8.82003 ± 0.00838**
FCT-NM J-value = **0.00178201 ± 0.00000169**
Air Shot 40Ar/36Ar = **303.8760 ± 0.4315**
Air Shot MDF = **0.99310013 ± 0.00067254 (LIN)**
Experiment Type = **Incremental Heating**
Extraction Method = **Bulk Laser Heating**
Heating = **0 sec**
Isolation = **6.00 min**
Instrument = **ARGUS-VI-D**
Preferred Age = **Plateau Age**
Age Classification = **Eruption Age**
IGSN = Undefined
Rock Class = Undefined
Lithology = **Basalt**
Lat-Lon = Undefined - Undefined
Age Equations = **Min et al. (2000)**
Negative Intensities = **Allowed**
Collector Calibrations = **40Ar 36Ar**
Decay 40K = **5.530 ± 0.048 E-10 1/a**
Decay 39Ar = **2.940 ± 0.016 E-07 1/h**
Decay 37Ar = **8.230 ± 0.012 E-04 1/h**
Decay 36Cl = **2.257 ± 0.015 E-06 1/a**
Decay 40K(EC,β^+) = **0.580 ± 0.009 E-10 1/a**
Decay 40K(β^-) = **4.950 ± 0.043 E-10 1/a**
Atmospheric 40/36(a) = **295.50**
Atmospheric 38/36(a) = **0.1869**
Production 39/37(ca) = **0.0006756 ± 0.0000089**
Production 38/37(ca) = **0.0000718 ± 0.0000092**
Production 36/37(ca) = **0.0002663 ± 0.0000004**
Production 40/39(k) = **0.003823 ± 0.000102**
Production 38/39(k) = **0.012031 ± 0.000019**
Production 36/38(cl) = **262.80 ± 1.71**
Scaling Ratio K/Ca = **0.430**
Abundance Ratio 40K/K = **1.1700 ± 0.0100 E-04**
Atomic Weight K = **39.0983 ± 0.0001 g**

Results

Results	40(a)/36(a) ± 2σ		40(r)/39(k) ± 2σ		Age ± 2σ (Ma)		MSWD	39Ar(k) (%,n)	K/Ca ± 2σ
**Age Plateau**			16.89381	± 0.04949<br>± 0.29%	**53.64**	**± 0.18<br>± 0.34%**	1.65<br>4%	94.90<br>19	0.0213 ± 0.0006
					Full External Error	± 1.22	1.67	2σ Confidence Limit	
					Analytical Error	± 0.15	1.2858	Error Magnification	
**Total Fusion Age**			16.89002	± 0.04695<br>± 0.28%	**53.62**	**± 0.18<br>± 0.33%**		26	0.0211 ± 0.0002
					Full External Error	± 1.21			
					Analytical Error	± 0.15			
**Normal Isochron**<br>Error Chron	**280.27**	**± 17.00<br>± 6.07%**	16.94150	± 0.06939<br>± 0.41%	**53.79**	**± 0.24<br>± 0.44%**	1.77<br>3%	94.90<br>19	
					Full External Error	± 1.23	1.69	2σ Confidence Limit	
					Analytical Error	± 0.22	1.3287	Error Magnification	
**Inverse Isochron**	**284.27**	**± 15.97<br>± 5.62%**	16.92676	± 0.06560<br>± 0.39%	**53.74**	**± 0.23<br>± 0.43%**	1.56<br>6%	94.90<br>19	
					Full External Error	± 1.23	1.69	2σ Confidence Limit	
					Analytical Error	± 0.21	1.2500	Error Magnification	
							19%	Spreading Factor	

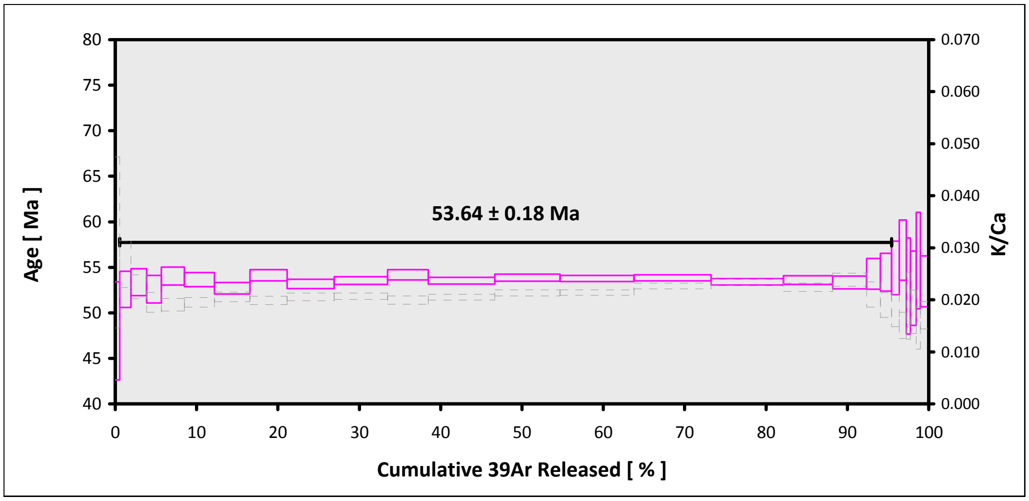

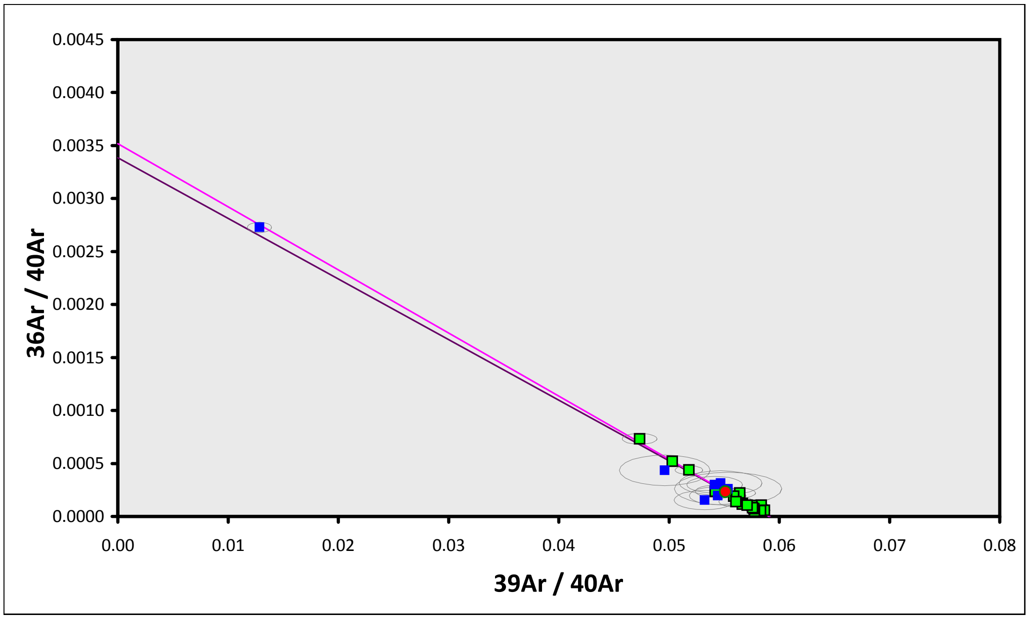

RR1310-D10-04 > Groundmass > RURUTU (13-INT-08)
TUVALU > RURUTU HOTSPOT
14-OSU-02 (2A24-14) > Incremental Heating > Kevin Konrad

Information on Analysis and Constants Used in Calculations

Project = **RURUTU (13-INT-08)**
Sample = **RR1310-D10-04**
Material = **Groundmass**
Location = **Rurutu Hotspot**
Region = **Tuvalu**
Analyst = **Kevin Konrad**
Irradiation = **14-OSU-02 (2A24-14)**
Position = **X: 0 | Y: 0 | Z/H: 28.6 mm**
FCT-NM Age = **28.201 ± 0.023 Ma**
FCT-NM Reference = **Kuiper et al. (2008)**
FCT-NM 40Ar/39Ar Ratio = **8.82225 ± 0.00838**
FCT-NM J-value = **0.00178156 ± 0.00000169**
Air Shot 40Ar/36Ar = **304.0110 ± 0.4074**
Air Shot MDF = **0.99299203 ± 0.00066245 (LIN)**
Experiment Type = **Incremental Heating**
Extraction Method = **Bulk Laser Heating**
Heating = **77 sec**
Isolation = **10.00 min**
Instrument = **ARGUS-VI-D**
Preferred Age = Undefined
Age Classification = Undefined
IGSN = Undefined
Rock Class = Undefined
Lithology = **Basalt**
Lat-Lon = Undefined - Undefined
Age Equations = **Min et al. (2000)**
Negative Intensities = **Allowed**
Collector Calibrations = **40Ar 36Ar**
Decay 40K = **5.530 ± 0.048 E-10 1/a**
Decay 39Ar = **2.940 ± 0.016 E-07 1/h**
Decay 37Ar = **8.230 ± 0.012 E-04 1/h**
Decay 36Cl = **2.257 ± 0.015 E-06 1/a**
Decay 40K(EC,β^+) = **0.580 ± 0.009 E-10 1/a**
Decay 40K(β^-) = **4.950 ± 0.043 E-10 1/a**
Atmospheric 40/36(a) = **295.50**
Atmospheric 38/36(a) = **0.1869**
Production 39/37(ca) = **0.0006756 ± 0.0000089**
Production 38/37(ca) = **0.0000718 ± 0.0000092**
Production 36/37(ca) = **0.0002663 ± 0.0000004**
Production 40/39(k) = **0.003823 ± 0.000102**
Production 38/39(k) = **0.012031 ± 0.000019**
Production 36/38(cl) = **262.80 ± 1.71**
Scaling Ratio K/Ca = **0.430**
Abundance Ratio 40K/K = **1.1700 ± 0.0100 E-04**
Atomic Weight K = **39.0983 ± 0.0001 g**

Results

Results	40(a)/36(a) ± 2σ	40(r)/39(k) ± 2σ	Age ± 2σ (Ma)	MSWD	39Ar(k) (%,n)	K/Ca ± 2σ
**Age Plateau**<br>Cannot Calculate						
**Total Fusion Age**		7.64953 ± 0.04303 ± 0.56%	**24.48 ± 0.14 ± 0.59%**<br>Full External Error ± 0.57<br>Analytical Error ± 0.14		36	0.0265 ± 0.0003
**Normal Isochron**<br>Cannot Calculate						
**Inverse Isochron**<br>Cannot Calculate						

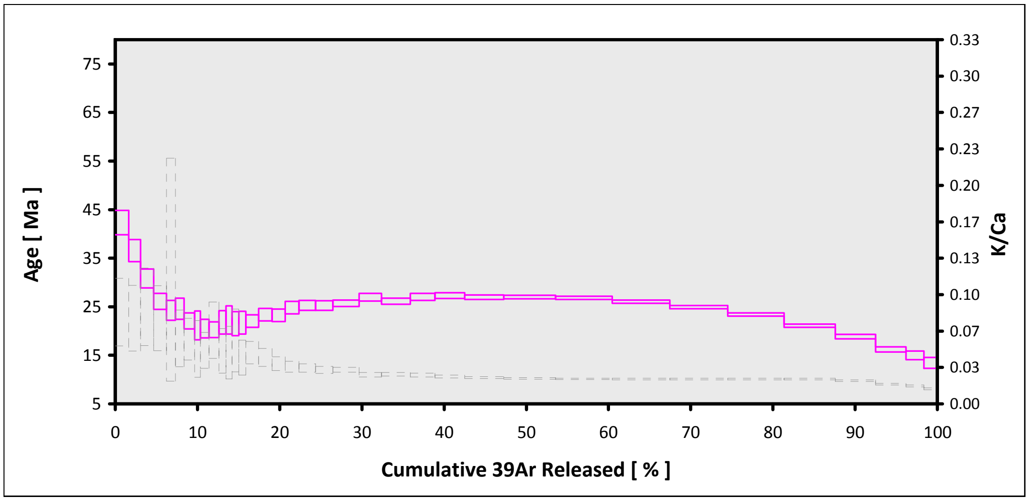

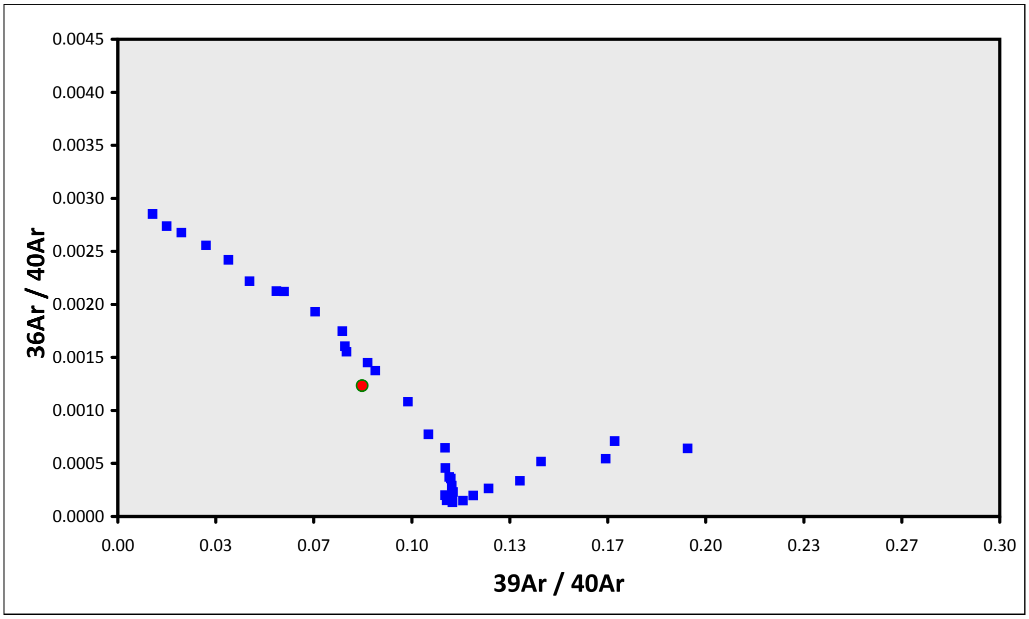

RR1310-D11-10 > Hornblende > RURUTU (13-INT-08)
TUVALU > RURUTU HOTSPOT
14-OSU-02 (2A19-14) > Incremental Heating > Kevin Konrad

Information on Analysis and Constants Used in Calculations

Project = **RURUTU (13-INT-08)**
Sample = **RR1310-D11-10**
Material = **Hornblende**
Location = **Rurutu Hotspot**
Region = **Tuvalu**
Analyst = **Kevin Konrad**
Irradiation = **14-OSU-02 (2A19-14)**
Position = **X: 0 | Y: 0 | Z/H: 23.7 mm**
FCT-NM Age = **28.201 ± 0.023 Ma**
FCT-NM Reference = **Kuiper et al. (2008)**
FCT-NM 40Ar/39Ar Ratio = **8.81730 ± 0.00838**
FCT-NM J-value = **0.00178256 ± 0.00000169**
Air Shot 40Ar/36Ar = **303.9860 ± 0.4165**
Air Shot MDF = **0.99301204 ± 0.00066613 (LIN)**
Experiment Type = **Incremental Heating**
Extraction Method = **Bulk Laser Heating**
Heating = **77 sec**
Isolation = **6.00 min**
Instrument = **ARGUS-VI-D**
Preferred Age = **Plateau Age**
Age Classification = **Eruption Age**
IGSN = Undefined
Rock Class = Undefined
Lithology = **Basalt**
Lat-Lon = Undefined - Undefined
Age Equations = **Min et al. (2000)**
Negative Intensities = **Allowed**
Collector Calibrations = **40Ar 36Ar**
Decay 40K = **5.530 ± 0.048 E-10 1/a**
Decay 39Ar = **2.940 ± 0.016 E-07 1/h**
Decay 37Ar = **8.230 ± 0.012 E-04 1/h**
Decay 36Cl = **2.257 ± 0.015 E-06 1/a**
Decay 40K(EC,β^+) = **0.580 ± 0.009 E-10 1/a**
Decay 40K(β^-) = **4.950 ± 0.043 E-10 1/a**
Atmospheric 40/36(a) = **295.50**
Atmospheric 38/36(a) = **0.1869**
Production 39/37(ca) = **0.0006756 ± 0.0000089**
Production 38/37(ca) = **0.0000718 ± 0.0000092**
Production 36/37(ca) = **0.0002663 ± 0.0000004**
Production 40/39(k) = **0.003823 ± 0.000102**
Production 38/39(k) = **0.012031 ± 0.000019**
Production 36/38(cl) = **262.80 ± 1.71**
Scaling Ratio K/Ca = **0.430**
Abundance Ratio 40K/K = **1.1700 ± 0.0100 E-04**
Atomic Weight K = **39.0983 ± 0.0001 g**

Results

Results	40(a)/36(a) ± 2σ		40(r)/39(k) ± 2σ		Age ± 2σ (Ma)		MSWD	39Ar(k) (%,n)	K/Ca ± 2σ
**Age Plateau**			16.62218	± 0.29291<br>± 1.76%	**52.80**	**± 0.92**<br>**± 1.75%**	0.92<br>51%	99.55<br>10	0.0067 ± 0.0016
					Full External Error	± 1.50	1.94	2σ Confidence Limit	
					Analytical Error	± 0.92	1.0000	Error Magnification	
**Total Fusion Age**			16.56911	± 0.43392<br>± 2.62%	**52.64**	**± 1.36**<br>**± 2.59%**		12	0.0061 ± 0.0002
					Full External Error	± 1.80			
					Analytical Error	± 1.36			
**Normal Isochron**<br>No Convergence	**360.64**	**± 59.29**<br>**± 16.44%**	15.47546	± 0.88579<br>± 5.72%	**49.21**	**± 2.78**<br>**± 5.65%**	1.13<br>34%	99.55<br>10	
					Full External Error	± 2.99	2.00	2σ Confidence Limit	
					Analytical Error	± 2.78	1.0625	Error Magnification	
**Inverse Isochron**	**287.15**	**± 38.23**<br>**± 13.31%**	16.72841	± 0.49085<br>± 2.93%	**53.13**	**± 1.54**<br>**± 2.90%**	0.98<br>45%	99.55<br>10	
					Full External Error	± 1.95	2.00	2σ Confidence Limit	
					Analytical Error	± 1.54	1.0000	Error Magnification	
							55%	Spreading Factor	

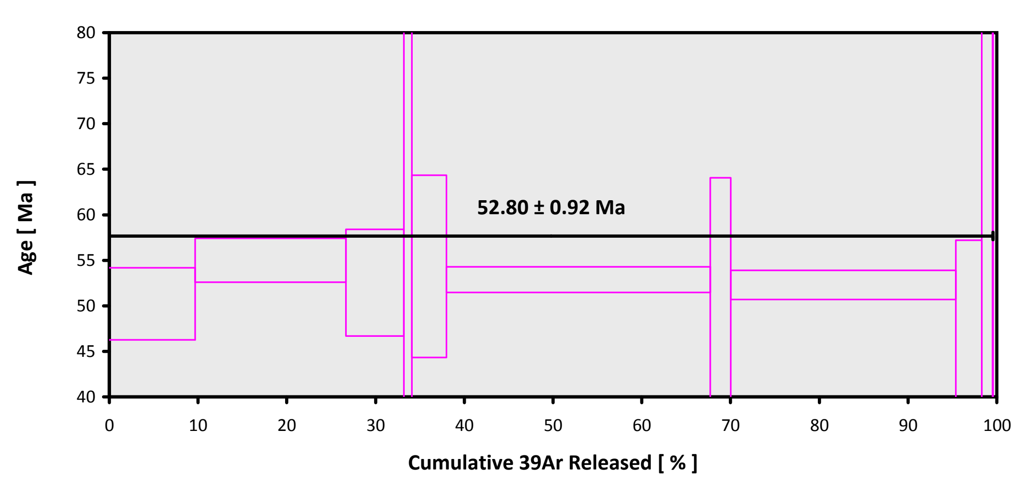

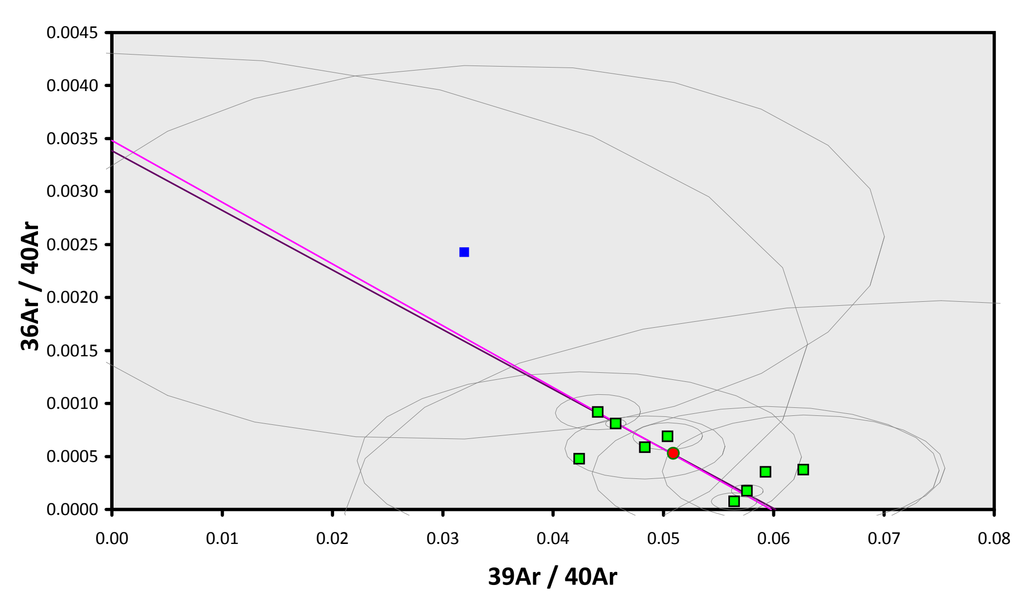

RR1310-D11-10 > Hornblende > RURUTU (13-INT-08) TUVALU > RURUTU HOTSPOT 14-OSU-02 (2A19-14) > Incremental Heating > Kevin Konrad

Information on Analysis and Constants Used in Calculations

Project = **RURUTU (13-INT-08)**
Sample = **RR1310-D11-10**
Material = **Hornblende**
Location = **Rurutu Hotspot**
Region = **Tuvalu**
Analyst = **Kevin Konrad**
Irradiation = **14-OSU-02 (2A19-14)**
Position = **X: 0 | Y: 0 | Z/H: 23.7 mm**
FCT-NM Age = **28.201 ± 0.023 Ma**
FCT-NM Reference = **Kuiper et al. (2008)**
FCT-NM 40Ar/39Ar Ratio = **8.81730 ± 0.00838**
FCT-NM J-value = **0.00178256 ± 0.00000169**
Air Shot 40Ar/36Ar = **303.8220 ± 0.4527**
Air Shot MDF = **0.99314339 ± 0.00068158 (LIN)**
Experiment Type = **Incremental Heating**
Extraction Method = **Bulk Laser Heating**
Heating = **0 sec**
Isolation = **3.00 min**
Instrument = **ARGUS-VI-D**
Preferred Age = **Plateau Age**
Age Classification = **Eruption Age**
IGSN = Undefined
Rock Class = Undefined
Lithology = **Basalt**
Lat-Lon = Undefined - Undefined
Age Equations = **Min et al. (2000)**
Negative Intensities = **Allowed**
Collector Calibrations = **40Ar 36Ar**
Decay 40K = **5.530 ± 0.048 E-10 1/a**
Decay 39Ar = **2.940 ± 0.016 E-07 1/h**
Decay 37Ar = **8.230 ± 0.012 E-04 1/h**
Decay 36Cl = **2.257 ± 0.015 E-06 1/a**
Decay 40K(EC,β^+) = **0.580 ± 0.009 E-10 1/a**
Decay 40K(β^-) = **4.950 ± 0.043 E-10 1/a**
Atmospheric 40/36(a) = **295.50**
Atmospheric 38/36(a) = **0.1869**
Production 39/37(ca) = **0.0006756 ± 0.0000089**
Production 38/37(ca) = **0.0000718 ± 0.0000092**
Production 36/37(ca) = **0.0002663 ± 0.0000004**
Production 40/39(k) = **0.003823 ± 0.000102**
Production 38/39(k) = **0.012031 ± 0.000019**
Production 36/38(cl) = **262.80 ± 1.71**
Scaling Ratio K/Ca = **0.430**
Abundance Ratio 40K/K = **1.1700 ± 0.0100 E-04**
Atomic Weight K = **39.0983 ± 0.0001 g**

Results

Results	40(a)/36(a) ± 2σ		40(r)/39(k) ± 2σ		Age ± 2σ (Ma)		MSWD	39Ar(k) (%,n)	K/Ca ± 2σ
**Age Plateau**			16.68027	± 0.43232<br>± 2.59%	**52.98**	**± 1.36<br>± 2.56%**	0.59<br>77%	98.15<br>8	0.0395 ± 0.0078
				Full External Error	± 1.80		2.07	2σ Confidence Limit	
				Analytical Error	± 1.35		1.0000	Error Magnification	
**Total Fusion Age**			16.31058	± 0.55556<br>± 3.41%	**51.83**	**± 1.74<br>± 3.36%**		9	0.0524 ± 0.0155
				Full External Error	± 2.09				
				Analytical Error	± 1.74				
**Normal Isochron**<br>No Convergence	**579.41**	**± 642.51<br>########**	14.96496	± 2.18422<br>± 14.60%	**47.61**	**± 6.86<br>± 14.41%**	0.68<br>67%	98.15<br>8	
				Full External Error	± 6.94		2.15	2σ Confidence Limit	
				Analytical Error	± 6.86		1.0000	Error Magnification	
**Inverse Isochron**	**27.44**	**± 39.03<br>########**	17.13447	± 0.64890<br>± 3.79%	**54.41**	**± 2.03<br>± 3.74%**	0.32<br>92%	98.15<br>8	
				Full External Error	± 2.37		2.15	2σ Confidence Limit	
				Analytical Error	± 2.03		1.0000	Error Magnification	
							37%	Spreading Factor	

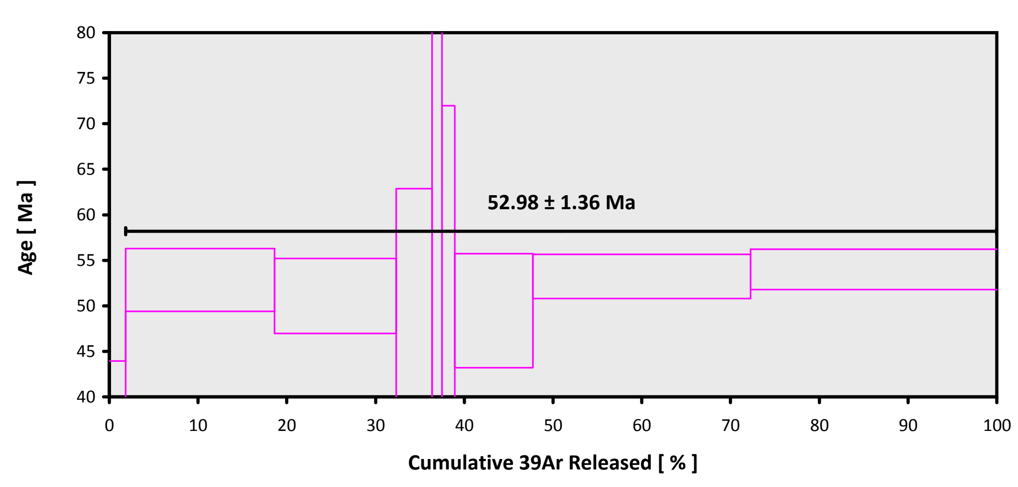

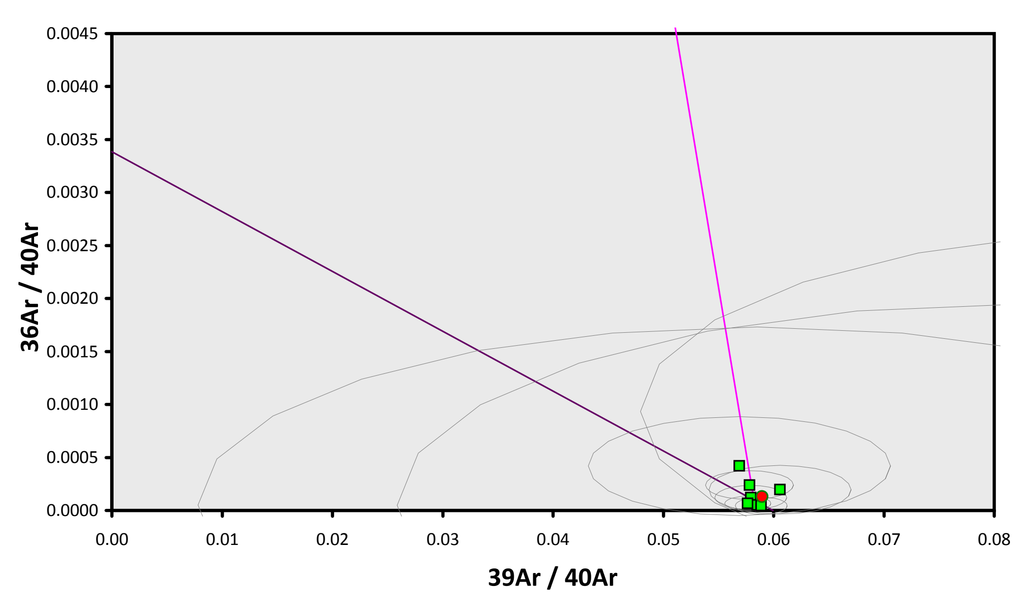

STACK > RR1310-D11-10 > Hornblende > RURUTU (13-INT-08)
TUVALU > RURUTU HOTSPOT
14-OSU-02 (2A19-14) > Incremental Heating > Kevin Konrad

Information on Analysis and Constants Used in Calculations

Project = **RURUTU (13-INT-08)**
Stack = **RR1310-D11-10**
Material = **Hornblende**
Location = **Rurutu Hotspot**
Region = **Tuvalu**
Analyst = **Kevin Konrad**
Irradiation = **14-OSU-02 (2A19-14)**
Position = **X: 0 | Y: 0 | Z/H: 23.7 mm**
FCT-NM Age = **28.201 ± 0.023 Ma**
FCT-NM Reference = **Kuiper et al. (2008)**
FCT-NM 40Ar/39Ar Ratio = **8.81730 ± 0.00838**
FCT-NM J-value = **0.00178256 ± 0.00000169**
Air Shot 40Ar/36Ar = **303.9860 ± 0.4165**
Air Shot MDF = **0.99301204 ± 0.00066613 (LIN)**
Experiment Type = **Incremental Heating**
Extraction Method = **Bulk Laser Heating**
Heating = **77 sec**
Isolation = **6.00 min**
Instrument = **ARGUS-VI-D**
Preferred Age = **Plateau Age**
Age Classification = **Eruption Age**
IGSN = Undefined
Rock Class = Undefined
Lithology = **Basalt**
Lat-Lon = Undefined - Undefined
Age Equations = **Min et al. (2000)**
Negative Intensities = **Allowed**
Collector Calibrations = **40Ar 39Ar 38Ar 37Ar 36Ar**
Decay 40K = **5.530 ± 0.048 E-10 1/a**
Decay 39Ar = **2.940 ± 0.016 E-07 1/h**
Decay 37Ar = **8.230 ± 0.012 E-04 1/h**
Decay 36Cl = **2.257 ± 0.015 E-06 1/a**
Decay 40K(EC,β^+) = **0.580 ± 0.009 E-10 1/a**
Decay 40K(β^-) = **4.950 ± 0.043 E-10 1/a**
Atmospheric 40/36(a) = **295.50**
Atmospheric 38/36(a) = **0.1869**
Production 39/37(ca) = **0.0006756 ± 0.0000089**
Production 38/37(ca) = **0.0000718 ± 0.0000092**
Production 36/37(ca) = **0.0002663 ± 0.0000004**
Production 40/39(k) = **0.003823 ± 0.000102**
Production 38/39(k) = **0.012031 ± 0.000019**
Production 36/38(cl) = **262.80 ± 1.71**
Scaling Ratio K/Ca = **0.430**
Abundance Ratio 40K/K = **1.1700 ± 0.0100 E-04**
Atomic Weight K = **39.0983 ± 0.0001 g**

Results

Results	40(a)/36(a) ± 2σ		40(r)/39(k) ± 2σ		Age ± 2σ (Ma)		MSWD	39Ar(k) (%,n)	K/Ca ± 2σ
**Age Plateau**			16.64046	± 0.24250<br>± 1.46%	**52.86**	**± 0.77<br>± 1.45%**	0.73<br>77%	99.02<br>18	0.0067 ± 0.0012
					Full External Error	± 1.41	1.69	2σ Confidence Limit	
					Analytical Error	± 0.76	1.0000	Error Magnification	
**Total Fusion Age**			16.47173	± 0.34219<br>± 2.08%	**52.33**	**± 1.08<br>± 2.06%**		21	0.0091 ± 0.0003
					Full External Error	± 1.59			
					Analytical Error	± 1.07			
**Normal Isochron**<br>No Convergence	**363.58**	**± 47.20<br>± 12.98%**	15.43950	± 0.65299<br>± 4.23%	**49.10**	**± 2.05<br>± 4.18%**	0.85<br>62%	99.02<br>18	
					Full External Error	± 2.33	1.71	2σ Confidence Limit	
					Analytical Error	± 2.05	1.0000	Error Magnification	
**Inverse Isochron**	**286.24**	**± 32.01<br>± 11.18%**	16.73179	± 0.34249<br>± 2.05%	**53.15**	**± 1.08<br>± 2.03%**	0.72<br>78%	99.02<br>18	
					Full External Error	± 1.61	1.71	2σ Confidence Limit	
					Analytical Error	± 1.07	1.0000	Error Magnification	
							66%	Spreading Factor	

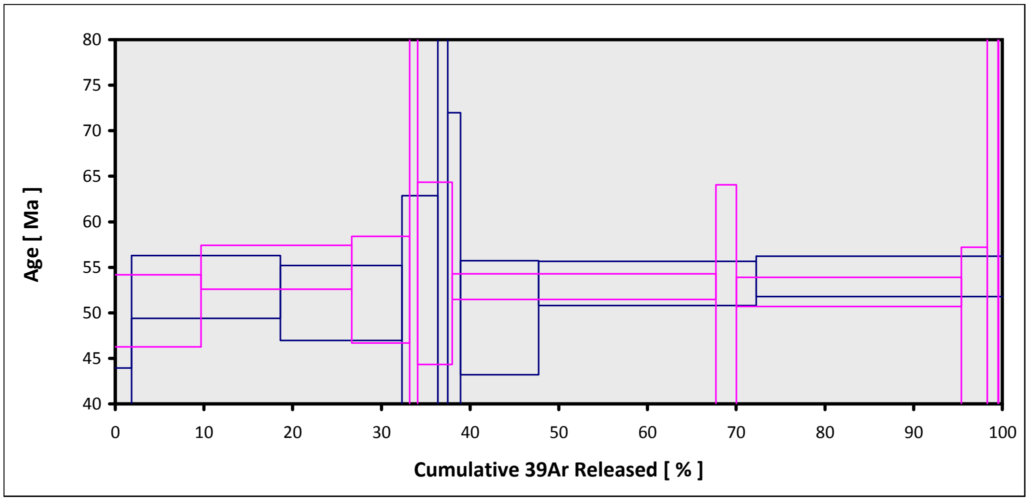

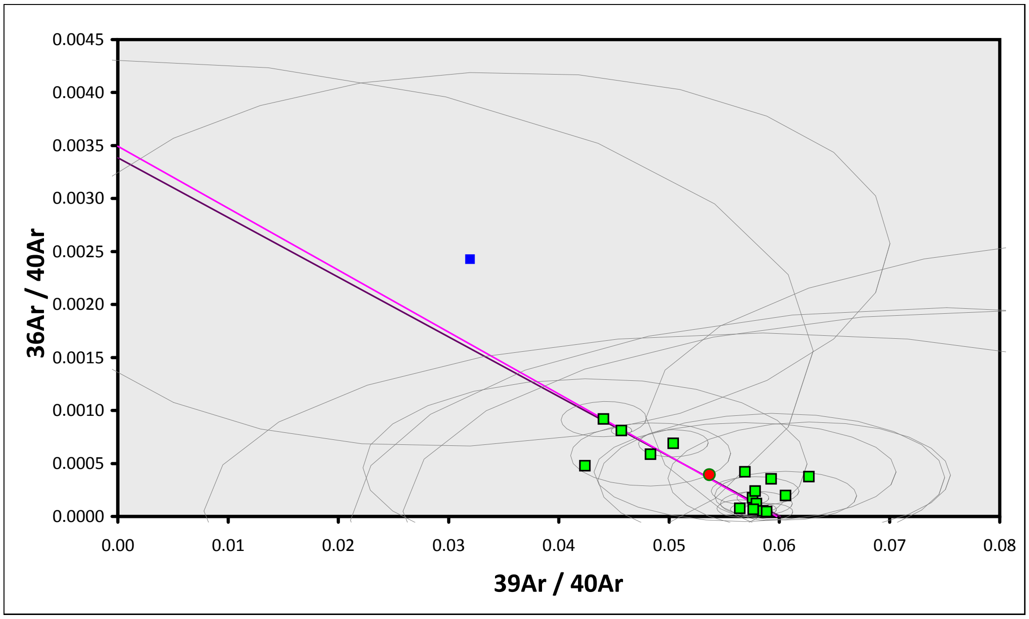

RR1310-D13-01 > Groundmass > RURUTU (13-INT-08)
TUVALU > RURUTU HOTSPOT
14-OSU-02 (2A36-14) > Incremental Heating > Kevin Konrad

Information on Analysis and Constants Used in Calculations

Project = **RURUTU (13-INT-08)**
Sample = **RR1310-D13-01**
Material = **Groundmass**
Location = **Rurutu Hotspot**
Region = **Tuvalu**
Analyst = **Kevin Konrad**
Irradiation = **14-OSU-02 (2A36-14)**
Position = **X: 0 | Y: 0 | Z/H: 42.3 mm**
FCT-NM Age = **28.201 ± 0.023 Ma**
FCT-NM Reference = **Kuiper et al. (2008)**
FCT-NM 40Ar/39Ar Ratio = **8.88521 ± 0.00844**
FCT-NM J-value = **0.00176894 ± 0.00000168**
Air Shot 40Ar/36Ar = **303.8830 ± 0.4315**
Air Shot MDF = **0.99309452 ± 0.00067252 (LIN)**
Experiment Type = **Incremental Heating**
Extraction Method = **Bulk Laser Heating**
Heating = **77 sec**
Isolation = **10.00 min**
Instrument = **ARGUS-VI-D**
Preferred Age = Undefined
Age Classification = Undefined
IGSN = Undefined
Rock Class = Undefined
Lithology = **Basalt**
Lat-Lon = Undefined - Undefined
Age Equations = **Min et al. (2000)**
Negative Intensities = **Allowed**
Collector Calibrations = **40Ar 36Ar**
Decay 40K = **5.530 ± 0.048 E-10 1/a**
Decay 39Ar = **2.940 ± 0.016 E-07 1/h**
Decay 37Ar = **8.230 ± 0.012 E-04 1/h**
Decay 36Cl = **2.257 ± 0.015 E-06 1/a**
Decay 40K(EC,β^+) = **0.580 ± 0.009 E-10 1/a**
Decay 40K(β^-) = **4.950 ± 0.043 E-10 1/a**
Atmospheric 40/36(a) = **295.50**
Atmospheric 38/36(a) = **0.1869**
Production 39/37(ca) = **0.0006756 ± 0.0000089**
Production 38/37(ca) = **0.0000718 ± 0.0000092**
Production 36/37(ca) = **0.0002663 ± 0.0000004**
Production 40/39(k) = **0.003823 ± 0.000102**
Production 38/39(k) = **0.012031 ± 0.000019**
Production 36/38(cl) = **262.80 ± 1.71**
Scaling Ratio K/Ca = **0.430**
Abundance Ratio 40K/K = **1.1700 ± 0.0100 E-04**
Atomic Weight K = **39.0983 ± 0.0001 g**

Results

Results	40(a)/36(a) ± 2σ	40(r)/39(k) ± 2σ	Age ± 2σ (Ma)	MSWD	39Ar(k) (%,n)	K/Ca ± 2σ
**Age Plateau**<br>Cannot Calculate						
**Total Fusion Age**		17.52944 ± 0.08137<br>± 0.46%	**55.22 ± 0.27**<br>**± 0.49%**<br>Full External Error ± 1.27<br>Analytical Error ± 0.25		35	0.0519 ± 0.0012
**Normal Isochron**<br>Cannot Calculate						
**Inverse Isochron**<br>Cannot Calculate						

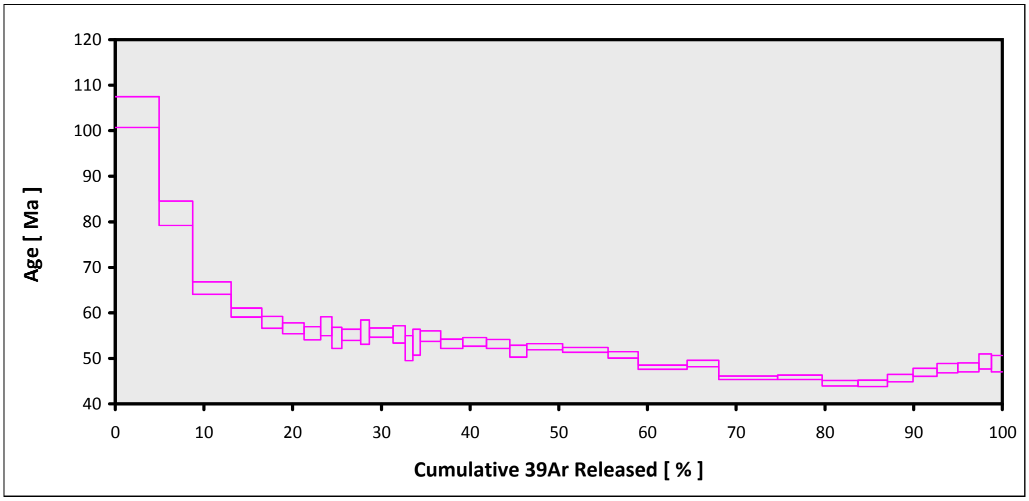

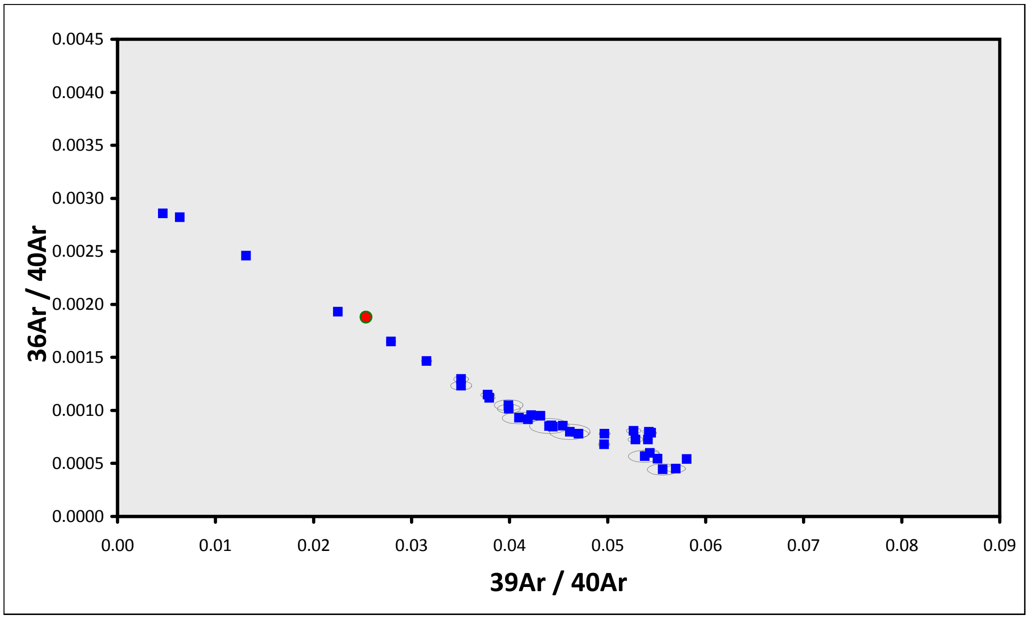

RR1310-D13-01 > Plagioclase > RURUTU (13-INT-08)
TUVALU > RURUTU HOTSPOT
14-OSU-02 (2A6-14) > Incremental Heating > Kevin Konrad

Information on Analysis and Constants Used in Calculations

Project = **RURUTU (13-INT-08)**
Sample = **RR1310-D13-01**
Material = **Plagioclase**
Location = **Rurutu Hotspot**
Region = **Tuvalu**
Analyst = **Kevin Konrad**
Irradiation = **14-OSU-02 (2A6-14)**
Position = **X: 0 | Y: 0 | Z/H: 8.4 mm**
FCT-NM Age = **28.201 ± 0.023 Ma**
FCT-NM Reference = **Kuiper et al. (2008)**
FCT-NM 40Ar/39Ar Ratio = **8.86144 ± 0.00842**
FCT-NM J-value = **0.00177368 ± 0.00000168**
Air Shot 40Ar/36Ar = **304.0570 ± 0.4074**
Air Shot MDF = **0.99295522 ± 0.00066235 (LIN)**
Experiment Type = **Incremental Heating**
Extraction Method = **Bulk Laser Heating**
Heating = **0 sec**
Isolation = **6.00 min**
Instrument = **ARGUS-VI-D**
Preferred Age = **Plateau Age**
Age Classification = **Eruption Age**
IGSN = Undefined
Rock Class = Undefined
Lithology = **Basalt**
Lat-Lon = Undefined - Undefined
Age Equations = **Min et al. (2000)**
Negative Intensities = **Allowed**
Collector Calibrations = **40Ar 36Ar**
Decay 40K = **5.530 ± 0.048 E-10 1/a**
Decay 39Ar = **2.940 ± 0.016 E-07 1/h**
Decay 37Ar = **8.230 ± 0.012 E-04 1/h**
Decay 36Cl = **2.257 ± 0.015 E-06 1/a**
Decay 40K(EC,β^+) = **0.580 ± 0.009 E-10 1/a**
Decay 40K(β^-) = **4.950 ± 0.043 E-10 1/a**
Atmospheric 40/36(a) = **295.50**
Atmospheric 38/36(a) = **0.1869**
Production 39/37(ca) = **0.0006756 ± 0.0000089**
Production 38/37(ca) = **0.0000718 ± 0.0000092**
Production 36/37(ca) = **0.0002663 ± 0.0000004**
Production 40/39(k) = **0.003823 ± 0.000102**
Production 38/39(k) = **0.012031 ± 0.000019**
Production 36/38(cl) = **262.80 ± 1.71**
Scaling Ratio K/Ca = **0.430**
Abundance Ratio 40K/K = **1.1700 ± 0.0100 E-04**
Atomic Weight K = **39.0983 ± 0.0001 g**

Results

Results	40(a)/36(a) ± 2σ	40(r)/39(k) ± 2σ	Age ± 2σ (Ma)	MSWD	39Ar(k) (%,n)	K/Ca ± 2σ
**Age Plateau**		15.97397 ± 0.05532	**50.52 ± 0.20**	2.63	92.01	0.0191 ± 0.0013
Error Mean		± 0.35%	**± 0.39%**	0%	18	
			Full External Error ± 1.15	1.69	2σ Confidence Limit	
			Analytical Error ± 0.17	1.6214	Error Magnification	
**Total Fusion Age**		15.99089 ± 0.03904	**50.58 ± 0.15**		24	0.0195 ± 0.0001
		± 0.24%	**± 0.31%**			
			Full External Error ± 1.14			
			Analytical Error ± 0.12			
**Normal Isochron**	**283.59 ± 44.74**	16.00089 ± 0.11134	**50.61 ± 0.36**	2.85	92.01	
No Convergence	**± 15.77%**	± 0.70%	**± 0.71%**	0%	18	
			Full External Error ± 1.19	1.71	2σ Confidence Limit	
			Analytical Error ± 0.35	1.6882	Error Magnification	
**Inverse Isochron**	**321.42 ± 44.81**	15.91796 ± 0.11405	**50.35 ± 0.37**	2.58	92.01	
Error Chron	**± 13.94%**	± 0.72%	**± 0.73%**	0%	18	
			Full External Error ± 1.19	1.71	2σ Confidence Limit	
			Analytical Error ± 0.36	1.6077	Error Magnification	
				9%	Spreading Factor	

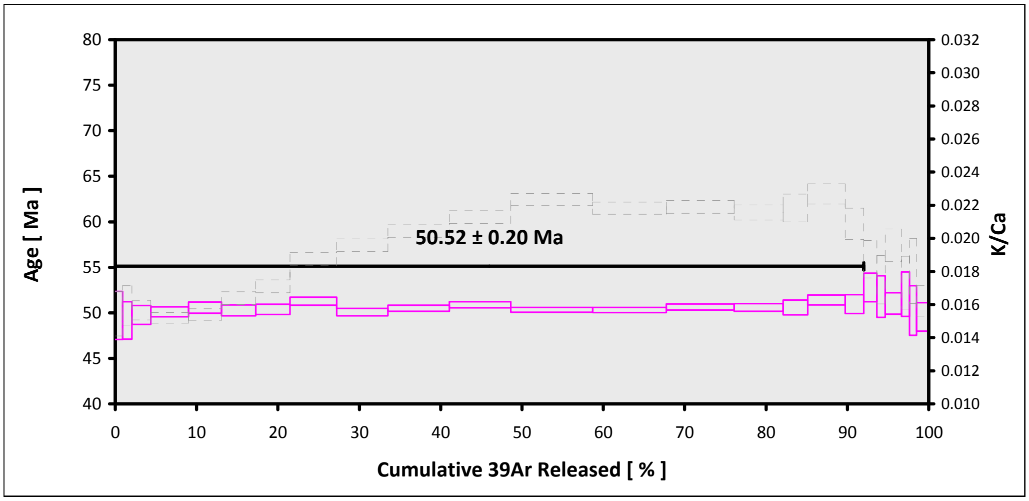

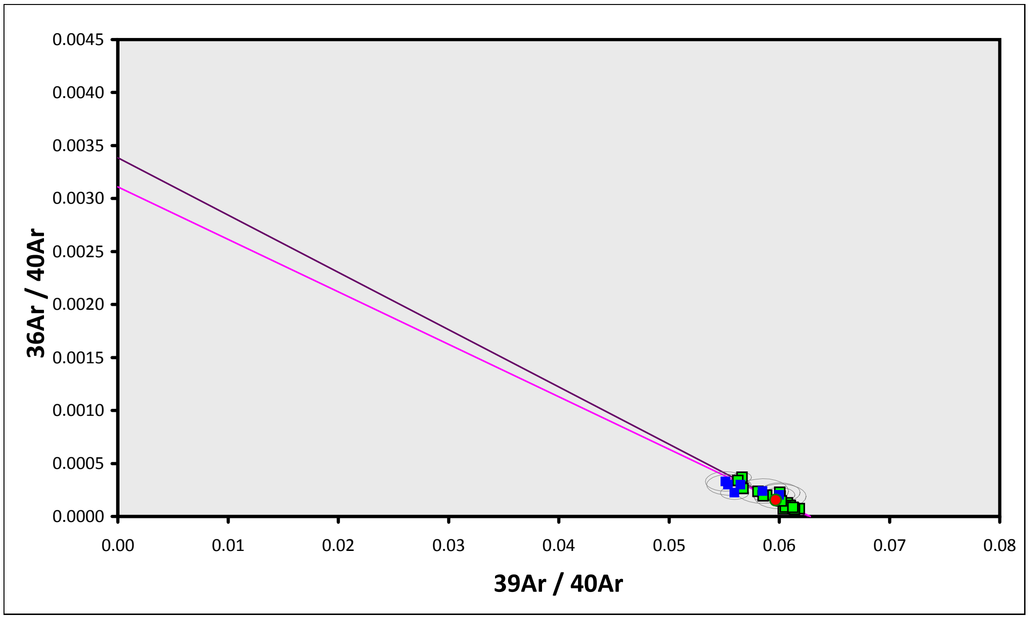

RR1310-D14-01 > Groundmass > RURUTU (13-INT-08)
TUVALU > RURUTU HOTSPOT
14-OSU-02 (2A40-14) > Incremental Heating > Kevin Konrad

Information on Analysis and Constants Used in Calculations

Project = **RURUTU (13-INT-08)**
Sample = **RR1310-D14-01**
Material = **Groundmass**
Location = **Rurutu Hotspot**
Region = **Tuvalu**
Analyst = **Kevin Konrad**
Irradiation = **14-OSU-02 (2A40-14)**
Position = **X: 0 | Y: 0 | Z/H: 47.9 mm**
FCT-NM Age = **28.201 ± 0.023 Ma**
FCT-NM Reference = **Kuiper et al. (2008)**
FCT-NM 40Ar/39Ar Ratio = **8.93178 ± 0.00840**
FCT-NM J-value = **0.00175972 ± 0.00000165**
Air Shot 40Ar/36Ar = **303.8810 ± 0.4133**
Air Shot MDF = **0.99309612 ± 0.00066514 (LIN)**
Experiment Type = **Incremental Heating**
Extraction Method = **Bulk Laser Heating**
Heating = **77 sec**
Isolation = **10.00 min**
Instrument = **ARGUS-VI-D**
Preferred Age = **Plateau Age**
Age Classification = **Eruption Age**
IGSN = Undefined
Rock Class = Undefined
Lithology = **Basalt**
Lat-Lon = Undefined - Undefined
Age Equations = **Min et al. (2000)**
Negative Intensities = **Allowed**
Collector Calibrations = **40Ar 36Ar**
Decay 40K = **5.530 ± 0.048 E-10 1/a**
Decay 39Ar = **2.940 ± 0.016 E-07 1/h**
Decay 37Ar = **8.230 ± 0.012 E-04 1/h**
Decay 36Cl = **2.257 ± 0.015 E-06 1/a**
Decay 40K(EC,β⁺) = **0.580 ± 0.009 E-10 1/a**
Decay 40K(β⁻) = **4.950 ± 0.043 E-10 1/a**
Atmospheric 40/36(a) = **295.50**
Atmospheric 38/36(a) = **0.1869**
Production 39/37(ca) = **0.0006756 ± 0.0000089**
Production 38/37(ca) = **0.0000718 ± 0.0000092**
Production 36/37(ca) = **0.0002663 ± 0.0000004**
Production 40/39(k) = **0.003823 ± 0.000102**
Production 38/39(k) = **0.012031 ± 0.000019**
Production 36/38(cl) = **262.80 ± 1.71**
Scaling Ratio K/Ca = **0.430**
Abundance Ratio 40K/K = **1.1700 ± 0.0100 E-04**
Atomic Weight K = **39.0983 ± 0.0001 g**

Results

Results	40(a)/36(a) ± 2σ	40(r)/39(k) ± 2σ	Age ± 2σ (Ma)	MSWD	39Ar(k) (%,n)	K/Ca ± 2σ
**Age Plateau**		13.88027 ± 0.18006 / ± 1.30%	**43.64 ± 0.57 / ± 1.30%**	0.58	33.68 / 92% 20	0.0504 ± 0.0050
			Full External Error ± 1.13	1.65	2σ Confidence Limit	
			Analytical Error ± 0.56	1.0000	Error Magnification	
**Total Fusion Age**		12.94051 ± 0.09388 / ± 0.73%	**40.72 ± 0.30 / ± 0.74%**		36	0.0287 ± 0.0008
			Full External Error ± 0.96			
			Analytical Error ± 0.29			
**Normal Isochron**	**296.66 ± 7.78 / ± 2.62%**	13.84743 ± 0.30535 / ± 2.21%	**43.54 ± 0.95 / ± 2.19%**	0.61	33.68 / 89% 20	
			Full External Error ± 1.36	1.67	2σ Confidence Limit	
			Analytical Error ± 0.95	1.0000	Error Magnification	
**Inverse Isochron**	**296.88 ± 7.80 / ± 2.63%**	13.84833 ± 0.30658 / ± 2.21%	**43.54 ± 0.96 / ± 2.20%**	0.62	33.68 / 89% 20	
			Full External Error ± 1.37	1.67	2σ Confidence Limit	
			Analytical Error ± 0.95	1.0000	Error Magnification	
				49%	Spreading Factor	

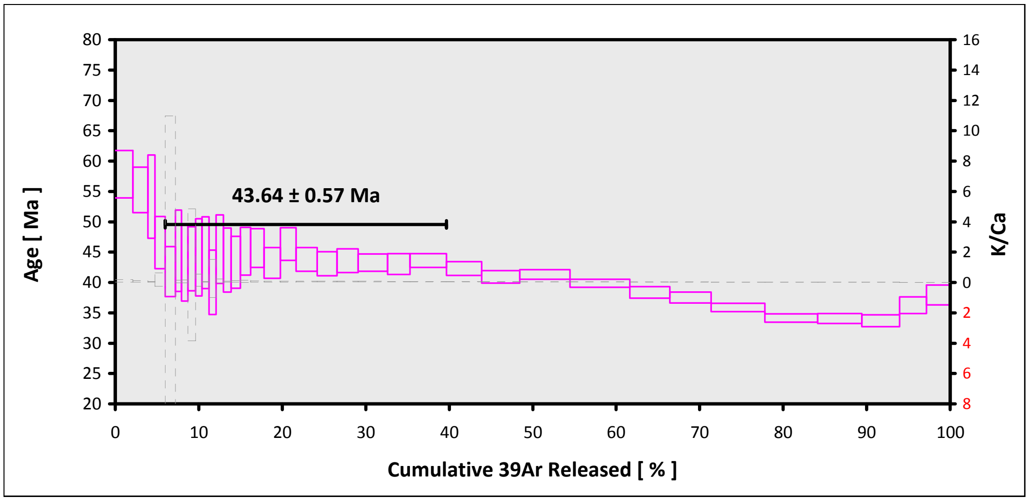

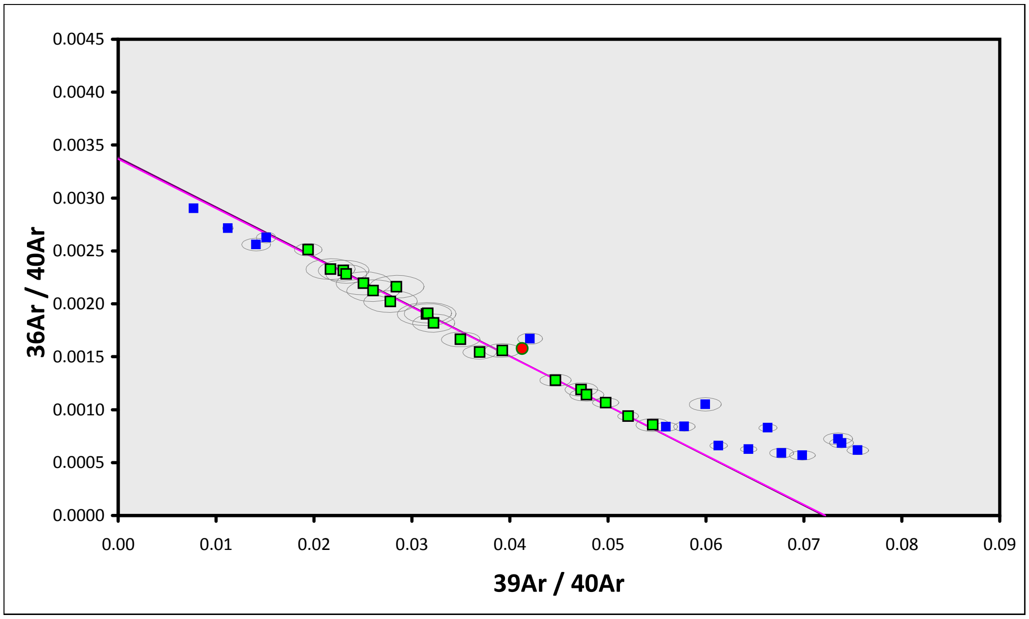

RR1310-D14-08 > Groundmass > RURUTU (13-INT-08)
TUVALU > RURUTU HOTSPOT
14-OSU-02 (2A39-14) > Incremental Heating > Kevin Konrad

Information on Analysis and Constants Used in Calculations

Project = **RURUTU (13-INT-08)**
Sample = **RR1310-D14-08**
Material = **Groundmass**
Location = **Rurutu Hotspot**
Region = **Tuvalu**
Analyst = **Kevin Konrad**
Irradiation = **14-OSU-02 (2A39-14)**
Position = **X: 0 | Y: 0 | Z/H: 46.6 mm**
FCT-NM Age = **28.201 ± 0.023 Ma**
FCT-NM Reference = **Kuiper et al. (2008)**
FCT-NM 40Ar/39Ar Ratio = **8.91989 ± 0.00838**
FCT-NM J-value = **0.00176206 ± 0.00000166**
Air Shot 40Ar/36Ar = **303.9060 ± 0.4164**
Air Shot MDF = **0.99307610 ± 0.00066630 (LIN)**
Experiment Type = **Incremental Heating**
Extraction Method = **Bulk Laser Heating**
Heating = **77 sec**
Isolation = **10.00 min**
Instrument = **ARGUS-VI-D**
Preferred Age = **Plateau Age**
Age Classification = **Eruption Age**
IGSN = Undefined
Rock Class = Undefined
Lithology = **Basalt**
Lat-Lon = Undefined - Undefined
Age Equations = **Min et al. (2000)**
Negative Intensities = **Allowed**
Collector Calibrations = **40Ar 36Ar**
Decay 40K = **5.530 ± 0.048 E-10 1/a**
Decay 39Ar = **2.940 ± 0.016 E-07 1/h**
Decay 37Ar = **8.230 ± 0.012 E-04 1/h**
Decay 36Cl = **2.257 ± 0.015 E-06 1/a**
Decay 40K(EC,β^+) = **0.580 ± 0.009 E-10 1/a**
Decay 40K(β^-) = **4.950 ± 0.043 E-10 1/a**
Atmospheric 40/36(a) = **342.60 ± 2.02**
Atmospheric 38/36(a) = **0.1869**
Production 39/37(ca) = **0.0006756 ± 0.0000089**
Production 38/37(ca) = **0.0000718 ± 0.0000092**
Production 36/37(ca) = **0.0002663 ± 0.0000004**
Production 40/39(k) = **0.003823 ± 0.000102**
Production 38/39(k) = **0.012031 ± 0.000019**
Production 36/38(cl) = **262.80 ± 1.71**
Scaling Ratio K/Ca = **0.430**
Abundance Ratio 40K/K = **1.1700 ± 0.0100 E-04**
Atomic Weight K = **39.0983 ± 0.0001 g**

Results

Results	40(a)/36(a) ± 2σ		40(r)/39(k) ± 2σ		Age ± 2σ (Ma)		MSWD	39Ar(k) (%,n)	K/Ca ± 2σ
**Age Plateau**			15.43775	± 0.04464<br>± 0.29%	**48.53**	**± 0.17**<br>**± 0.34%**	0.95<br>53%	50.58<br>24	0.119 ± 0.011
					Full External Error	± 1.10	1.59	2σ Confidence Limit	
					Analytical Error	± 0.14	1.0000	Error Magnification	
**Total Fusion Age**			14.70810	± 0.03136<br>± 0.21%	**46.27**	**± 0.13**<br>**± 0.28%**		36	0.140 ± 0.004
					Full External Error	± 1.05			
					Analytical Error	± 0.10			
**Normal Isochron**	**342.32**	**± 3.98**<br>**± 1.16%**	15.43730	± 0.08107<br>± 0.53%	**48.53**	**± 0.27**<br>**± 0.55%**	1.12<br>31%	50.58<br>24	
					Full External Error	± 1.12	1.60	2σ Confidence Limit	
					Analytical Error	± 0.25	1.0591	Error Magnification	
**Inverse Isochron**	**342.34**	**± 3.99**<br>**± 1.16%**	15.43977	± 0.08132<br>± 0.53%	**48.54**	**± 0.27**<br>**± 0.55%**	1.13<br>31%	50.58<br>24	
					Full External Error	± 1.12	1.60	2σ Confidence Limit	
					Analytical Error	± 0.25	1.0614	Error Magnification	
							44%	Spreading Factor	

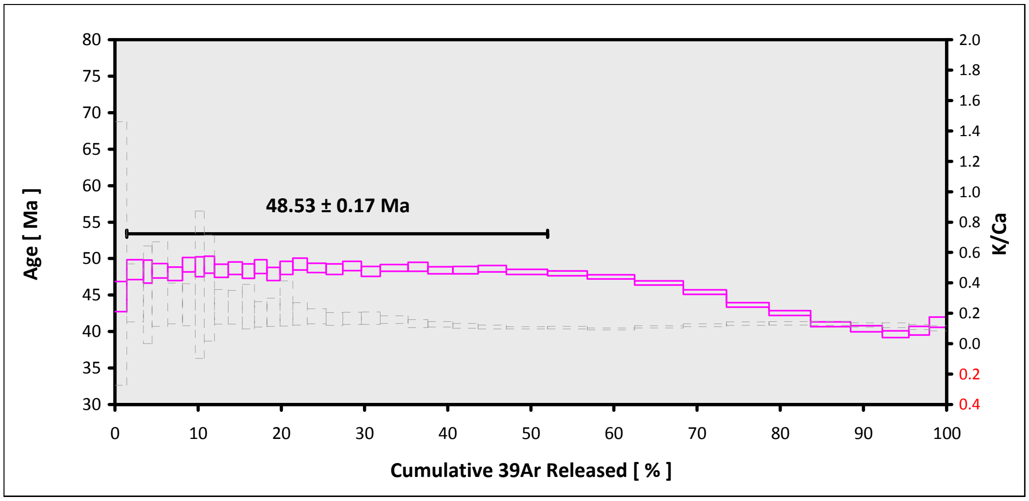

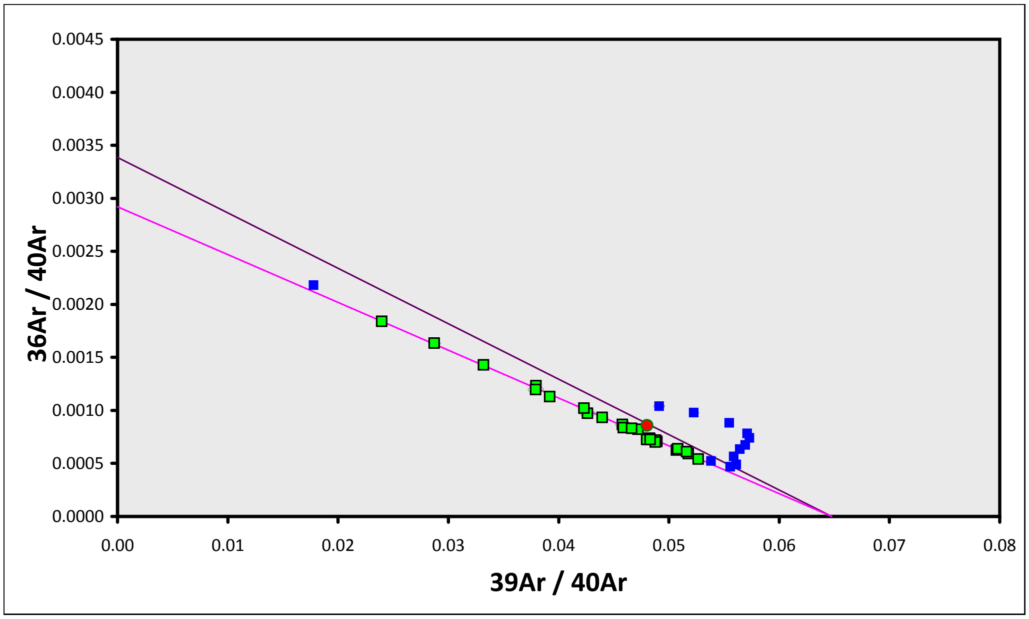

RR1310-D14-08 > Plagioclase > RURUTU (13-INT-08)
TUVALU > RURUTU HOTSPOT
14-OSU-02 (2A12-14) > Incremental Heating > Kevin Konrad

Information on Analysis and Constants Used in Calculations

Project = **RURUTU (13-INT-08)**
Sample = **RR1310-D14-08**
Material = **Plagioclase**
Location = **Rurutu Hotspot**
Region = **Tuvalu**
Analyst = **Kevin Konrad**
Irradiation = **14-OSU-02 (2A12-14)**
Position = **X: 0 | Y: 0 | Z/H: 17 mm**
FCT-NM Age = **28.201 ± 0.023 Ma**
FCT-NM Reference = **Kuiper et al. (2008)**
FCT-NM 40Ar/39Ar Ratio = **8.82552 ± 0.00838**
FCT-NM J-value = **0.00178090 ± 0.00000169**
Air Shot 40Ar/36Ar = **304.0280 ± 0.4074**
Air Shot MDF = **0.99297842 ± 0.00066241 (LIN)**
Experiment Type = **Incremental Heating**
Extraction Method = **Bulk Laser Heating**
Heating = **0 sec**
Isolation = **6.00 min**
Instrument = **ARGUS-VI-D**
Preferred Age = **Plateau Age**
Age Classification = **Eruption Age**
IGSN = Undefined
Rock Class = Undefined
Lithology = **Basalt**
Lat-Lon = Undefined - Undefined
Age Equations = **Min et al. (2000)**
Negative Intensities = **Allowed**
Collector Calibrations = **40Ar 36Ar**
Decay 40K = **5.530 ± 0.048 E-10 1/a**
Decay 39Ar = **2.940 ± 0.016 E-07 1/h**
Decay 37Ar = **8.230 ± 0.012 E-04 1/h**
Decay 36Cl = **2.257 ± 0.015 E-06 1/a**
Decay 40K(EC,β^+) = **0.580 ± 0.009 E-10 1/a**
Decay 40K(β^-) = **4.950 ± 0.043 E-10 1/a**
Atmospheric 40/36(a) = **295.50**
Atmospheric 38/36(a) = **0.1869**
Production 39/37(ca) = **0.0006756 ± 0.0000089**
Production 38/37(ca) = **0.0000718 ± 0.0000092**
Production 36/37(ca) = **0.0002663 ± 0.0000004**
Production 40/39(k) = **0.003823 ± 0.000102**
Production 38/39(k) = **0.012031 ± 0.000019**
Production 36/38(cl) = **262.80 ± 1.71**
Scaling Ratio K/Ca = **0.430**
Abundance Ratio 40K/K = **1.1700 ± 0.0100 E-04**
Atomic Weight K = **39.0983 ± 0.0001 g**

Results	40(a)/36(a) ± 2σ	40(r)/39(k) ± 2σ	Age ± 2σ (Ma)	MSWD	39Ar(k) (%,n)	K/Ca ± 2σ
**Age Plateau** Error Mean		15.68513 ± 0.04841 ± 0.31%	**49.82 ± 0.18 ± 0.36%**	1.85 / 3%	70.92 / 15	0.0180 ± 0.0008
			Full External Error ± 1.13	1.76	2σ Confidence Limit	
			Analytical Error ± 0.15	1.3616	Error Magnification	
**Total Fusion Age**		15.79261 ± 0.03436 ± 0.22%	**50.16 ± 0.14 ± 0.28%**		24	0.0182 ± 0.0001
			Full External Error ± 1.13			
			Analytical Error ± 0.11			
**Normal Isochron** No Convergence	**307.66 ± 12.27 ± 3.99%**	15.58992 ± 0.08575 ± 0.55%	**49.52 ± 0.28 ± 0.57%**	2.27 / 1%	70.92 / 15	
			Full External Error ± 1.15	1.78	2σ Confidence Limit	
			Analytical Error ± 0.27	1.5075	Error Magnification	
**Inverse Isochron** Error Chron	**300.54 ± 11.12 ± 3.70%**	15.65916 ± 0.07703 ± 0.49%	**49.74 ± 0.26 ± 0.52%**	1.87 / 3%	70.92 / 15	
			Full External Error ± 1.14	1.78	2σ Confidence Limit	
			Analytical Error ± 0.24	1.3689	Error Magnification	
				46%	Spreading Factor	

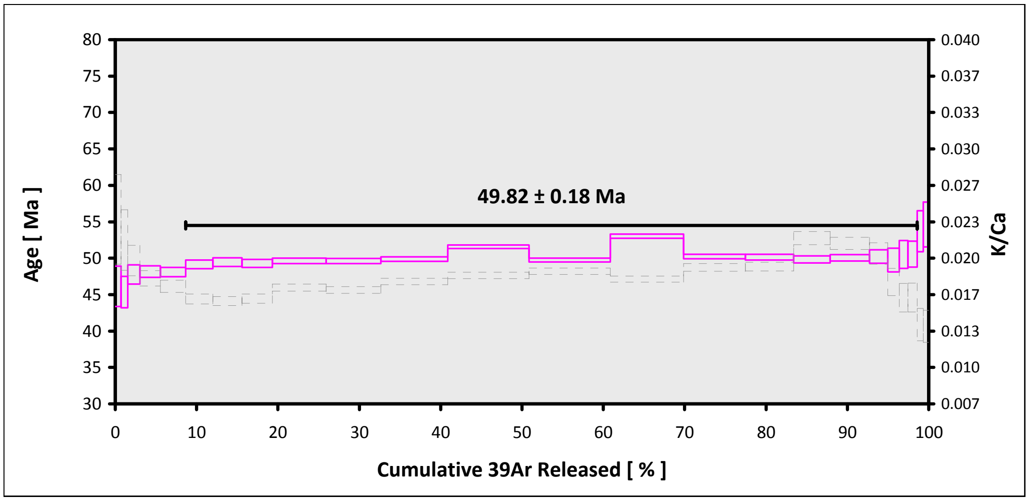

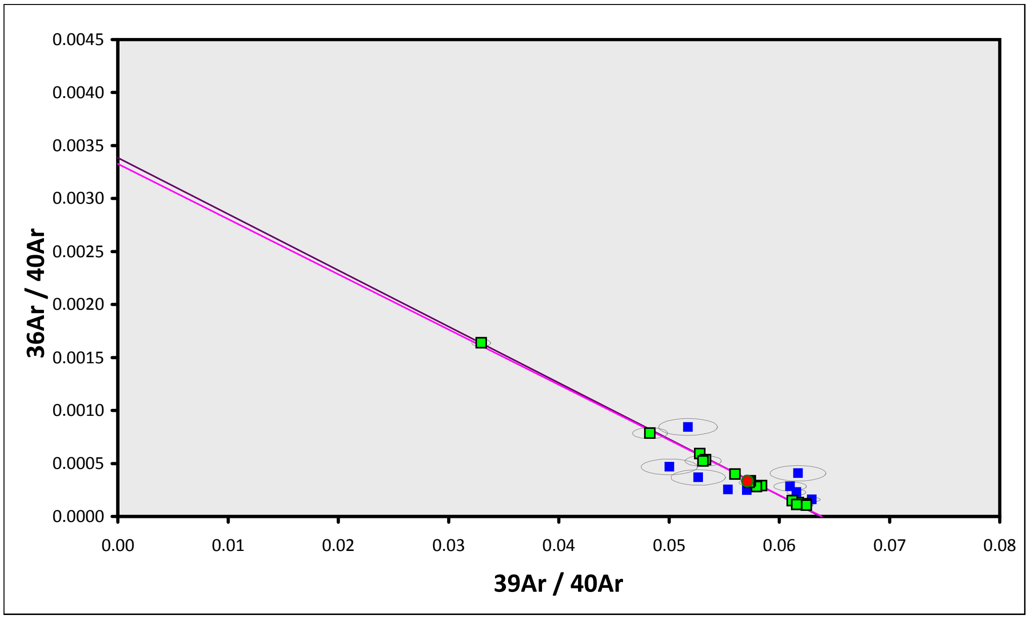

RR1310-D15-02 > Groundmass > RURUTU (13-INT-08)
TUVALU > RURUTU HOTSPOT
14-OSU-02 (2A38-14) > Incremental Heating > Kevin Konrad

Information on Analysis and Constants Used in Calculations

Project = **RURUTU (13-INT-08)**
Sample = **RR1310-D15-02**
Material = **Groundmass**
Location = **Rurutu Hotspot**
Region = **Tuvalu**
Analyst = **Kevin Konrad**
Irradiation = **14-OSU-02 (2A38-14)**
Position = **X: 0 | Y: 0 | Z/H: 45.3 mm**
FCT-NM Age = **28.201 ± 0.023 Ma**
FCT-NM Reference = **Kuiper et al (2008)**
FCT-NM 40Ar/39Ar Ratio = **8.90865 ± 0.00837**
FCT-NM J-value = **0.00176428 ± 0.00000166**
Air Shot 40Ar/36Ar = **303.9430 ± 0.4103**
Air Shot MDF = **0.99304647 ± 0.00066380 (LIN)**
Experiment Type = **Incremental Heating**
Extraction Method = **Bulk Laser Heating**
Heating = **77 sec**
Isolation = **10.00 min**
Instrument = **ARGUS-VI-D**
Preferred Age = **Inverse Isochron**
Age Classification = **Eruption Age**
IGSN = Undefined
Rock Class = Undefined
Lithology = **Basalt**
Lat-Lon = Undefined - Undefined
Age Equations = **Min et al. (2000)**
Negative Intensities = **Allowed**
Collector Calibrations = **40Ar 36Ar**
Decay 40K = **5.530 ± 0.048 E-10 1/a**
Decay 39Ar = **2.940 ± 0.016 E-07 1/h**
Decay 37Ar = **8.230 ± 0.012 E-04 1/h**
Decay 36Cl = **2.257 ± 0.015 E-06 1/a**
Decay 40K(EC,β^+) = **0.580 ± 0.009 E-10 1/a**
Decay 40K(β^-) = **4.950 ± 0.043 E-10 1/a**
Atmospheric 40/36(a) = **295.50**
Atmospheric 38/36(a) = **0.1869**
Production 39/37(ca) = **0.0006756 ± 0.0000089**
Production 38/37(ca) = **0.0000718 ± 0.0000092**
Production 36/37(ca) = **0.0002663 ± 0.0000004**
Production 40/39(k) = **0.003823 ± 0.000102**
Production 38/39(k) = **0.012031 ± 0.000019**
Production 36/38(cl) = **262.80 ± 1.71**
Scaling Ratio K/Ca = **0.430**
Abundance Ratio 40K/K = **1.1700 ± 0.0100 E-04**
Atomic Weight K = **39.0983 ± 0.0001 g**

Results

Results	40(a)/36(a) ± 2σ		40(r)/39(k) ± 2σ		Age ± 2σ (Ma)		MSWD	39Ar(k) (%,n)	K/Ca ± 2σ
**Age Plateau**			15.57976	± 0.05512<br>± 0.35%	**49.03**	**± 0.19<br>± 0.40%**	1.42<br>10%	65.15<br>22	0.070 ± 0.006
				Full External Error	± 1.12		1.62	2σ Confidence Limit	
				Analytical Error	± 0.17		1.1917	Error Magnification	
**Total Fusion Age**			14.95832	± 0.04381<br>± 0.29%	**47.10**	**± 0.16<br>± 0.34%**		36	0.070 ± 0.002
				Full External Error	± 1.07				
				Analytical Error	± 0.14				
**Normal Isochron**	**361.03**	**± 38.66<br>± 10.71%**	15.47936	± 0.06718<br>± 0.43%	**48.72**	**± 0.23<br>± 0.47%**	0.88<br>62%	65.15<br>22	
				Full External Error	± 1.12		1.63	2σ Confidence Limit	
				Analytical Error	± 0.21		1.0000	Error Magnification	
**Inverse Isochron**	**358.85**	**± 38.50<br>± 10.73%**	15.50556	± 0.06735<br>± 0.43%	**48.80**	**± 0.23<br>± 0.47%**	0.94<br>54%	65.15<br>22	
				Full External Error	± 1.12		1.63	2σ Confidence Limit	
				Analytical Error	± 0.21		1.0000	Error Magnification	
							19%	Spreading Factor	

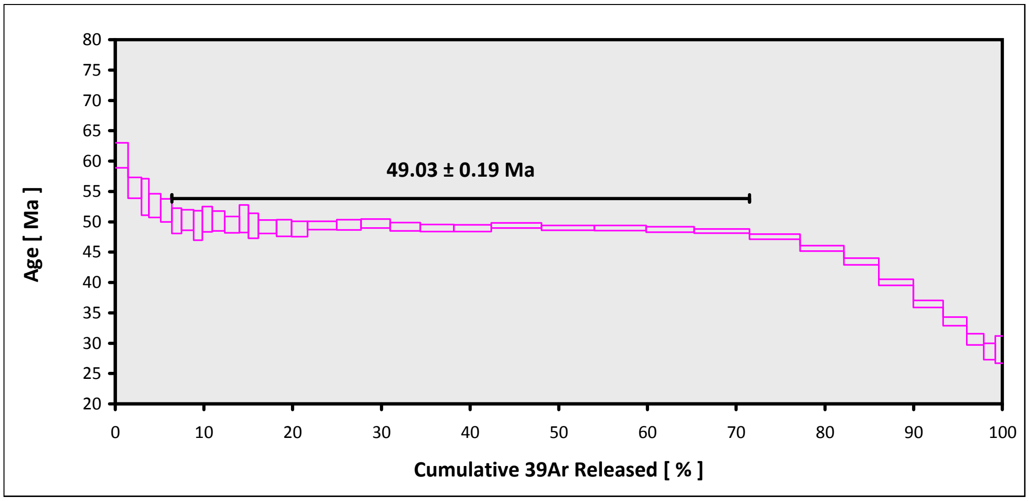

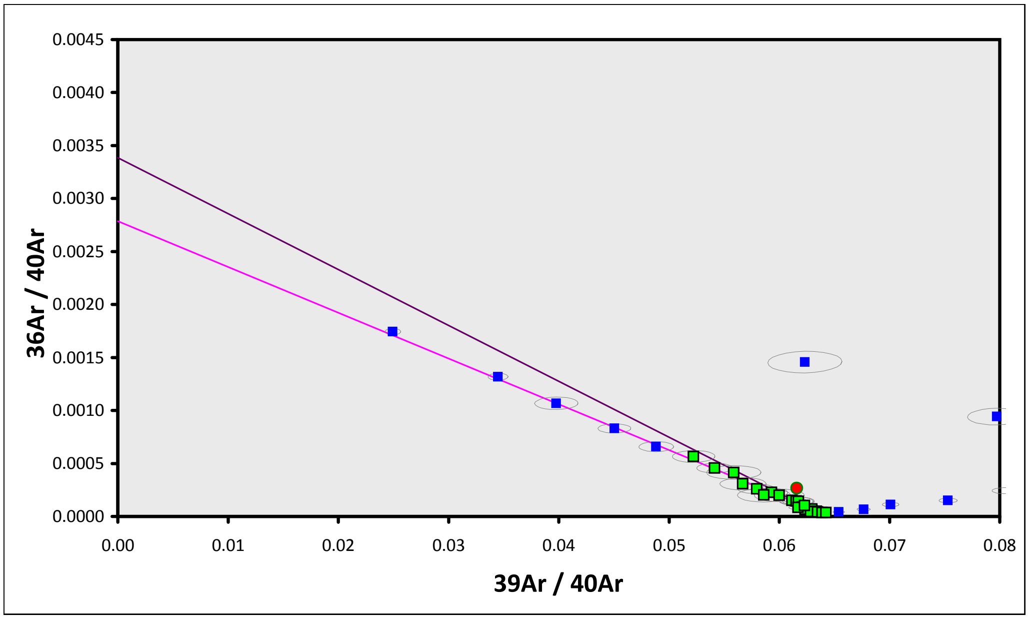

RR1310-D15-12 > Groundmass > RURUTU (13-INT-08)
TUVALU > RURUTU HOTSPOT
14-OSU-02 (2A54-14) > Incremental Heating > Kevin Konrad

Information on Analysis and Constants Used in Calculations

Project = **RURUTU (13-INT-08)**
Sample = **RR1310-D15-12**
Material = **Groundmass**
Location = **Rurutu Hotspot**
Region = **Tuvalu**
Analyst = **Kevin Konrad**
Irradiation = **14-OSU-02 (2A54-14)**
Position = **X: 0 | Y: 0 | Z/H: 63.8 mm**
FCT-NM Age = **28.201 ± 0.023 Ma**
FCT-NM Reference = **Kuiper et al. (2008)**
FCT-NM 40Ar/39Ar Ratio = **9.12991 ± 0.00840**
FCT-NM J-value = **0.00172153 ± 0.00000158**
Air Shot 40Ar/36Ar = **304.0620 ± 0.4044**
Air Shot MDF = **0.99295122 ± 0.00066114 (LIN)**
Experiment Type = **Incremental Heating**
Extraction Method = **Bulk Laser Heating**
Heating = **77 sec**
Isolation = **10.00 min**
Instrument = **ARGUS-VI-D**
Preferred Age = Undefined
Age Classification = Undefined
IGSN = Undefined
Rock Class = Undefined
Lithology = **Basalt**
Lat-Lon = Undefined - Undefined
Age Equations = **Min et al. (2000)**
Negative Intensities = **Allowed**
Collector Calibrations = **40Ar 36Ar**
Decay 40K = **5.530 ± 0.048 E-10 1/a**
Decay 39Ar = **2.940 ± 0.016 E-07 1/h**
Decay 37Ar = **8.230 ± 0.012 E-04 1/h**
Decay 36Cl = **2.257 ± 0.015 E-06 1/a**
Decay 40K(EC,β^+) = **0.580 ± 0.009 E-10 1/a**
Decay 40K(β^-) = **4.950 ± 0.043 E-10 1/a**
Atmospheric 40/36(a) = **295.50**
Atmospheric 38/36(a) = **0.1869**
Production 39/37(ca) = **0.0006756 ± 0.0000089**
Production 38/37(ca) = **0.0000718 ± 0.0000092**
Production 36/37(ca) = **0.0002663 ± 0.0000004**
Production 40/39(k) = **0.003823 ± 0.000102**
Production 38/39(k) = **0.012031 ± 0.000019**
Production 36/38(cl) = **262.80 ± 1.71**
Scaling Ratio K/Ca = **0.430**
Abundance Ratio 40K/K = **1.1700 ± 0.0100 E-04**
Atomic Weight K = **39.0983 ± 0.0001 g**

Results	40(a)/36(a) ± 2σ	40(r)/39(k) ± 2σ	Age ± 2σ (Ma)	MSWD	39Ar(k) (%,n)	K/Ca ± 2σ
**Age Plateau**<br>Cannot Calculate						
**Total Fusion Age**		15.08345 ± 0.01882<br>± 0.12%	**46.36 ± 0.10**<br>**± 0.22%**<br>Full External Error ± 1.04<br>Analytical Error ± 0.06		36	0.084 ± 0.001
**Normal Isochron**<br>Cannot Calculate						
**Inverse Isochron**<br>Cannot Calculate						

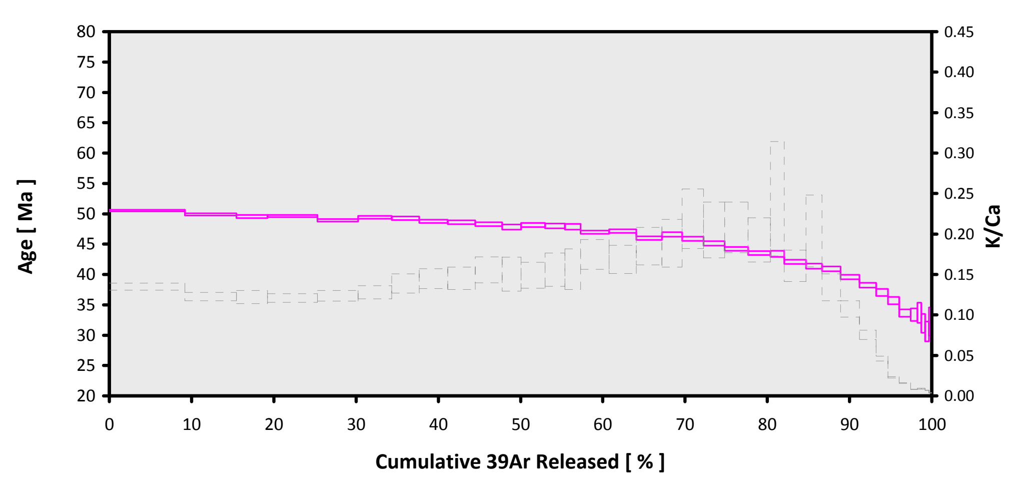

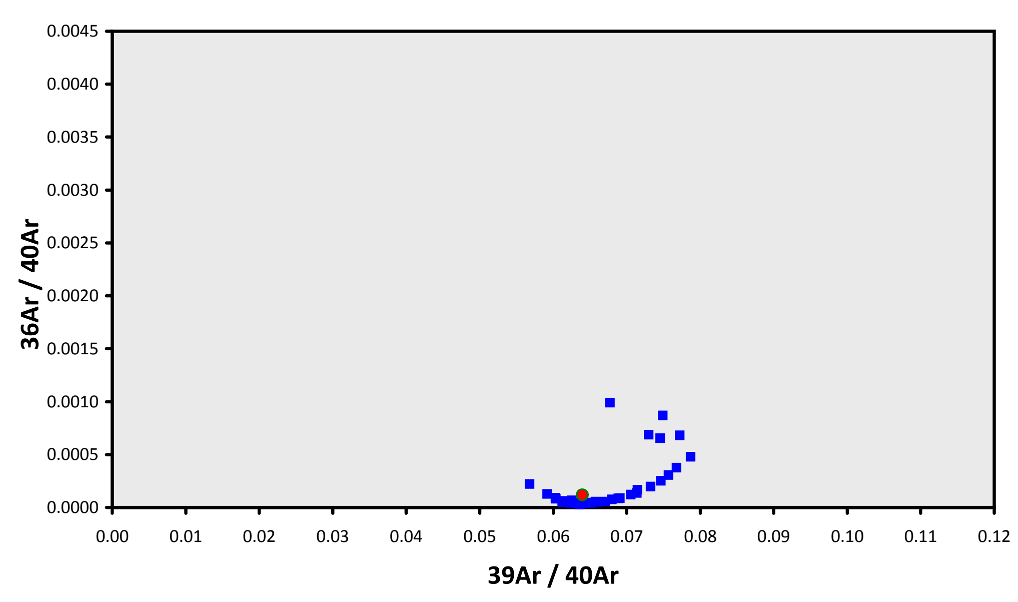

RR1310-D15-12 > Groundmass > RURUTU (13-INT-08)
TUVALU > RURUTU HOTSPOT
14-OSU-02 (2A54-14) > Incremental Heating > Kevin Konrad

Information on Analysis and Constants Used in Calculations

Project = **RURUTU (13-INT-08)**
Sample = **RR1310-D15-12**
Material = **Groundmass**
Location = **Rurutu Hotspot**
Region = **Tuvalu**
Analyst = **Kevin Konrad**
Irradiation = **14-OSU-02 (2A54-14)**
Position = **X: 0 | Y: 0 | Z/H: 63.8 mm**
FCT-NM Age = **28.201 ± 0.023 Ma**
FCT-NM Reference = **Kuiper et al. (2008)**
FCT-NM 40Ar/39Ar Ratio = **9.12991 ± 0.00840**
FCT-NM J-value = **0.00172153 ± 0.00000158**
Air Shot 40Ar/36Ar = **303.7380 ± 0.4647**
Air Shot MDF = **0.99321072 ± 0.00068700 (LIN)**
Experiment Type = **Incremental Heating**
Extraction Method = **Bulk Laser Heating**
Heating = **77 sec**
Isolation = **10.00 min**
Instrument = **ARGUS-VI-D**
Preferred Age = **Plateau Age**
Age Classification = **Eruption Age**
IGSN = Undefined
Rock Class = Undefined
Lithology = **Basalt**
Lat-Lon = Undefined - Undefined
Age Equations = **Min et al. (2000)**
Negative Intensities = **Allowed**
Collector Calibrations = **40Ar 36Ar**
Decay 40K = **5.530 ± 0.048 E-10 1/a**
Decay 39Ar = **2.940 ± 0.016 E-07 1/h**
Decay 37Ar = **8.230 ± 0.012 E-04 1/h**
Decay 36Cl = **2.257 ± 0.015 E-06 1/a**
Decay 40K(EC,β^+) = **0.580 ± 0.009 E-10 1/a**
Decay 40K(β^-) = **4.950 ± 0.043 E-10 1/a**
Atmospheric 40/36(a) = **360.70 ± 5.77**
Atmospheric 38/36(a) = **0.1869**
Production 39/37(ca) = **0.0006756 ± 0.0000089**
Production 38/37(ca) = **0.0000718 ± 0.0000092**
Production 36/37(ca) = **0.0002663 ± 0.0000004**
Production 40/39(k) = **0.003823 ± 0.000102**
Production 38/39(k) = **0.012031 ± 0.000019**
Production 36/38(cl) = **262.80 ± 1.71**
Scaling Ratio K/Ca = **0.430**
Abundance Ratio 40K/K = **1.1700 ± 0.0100 E-04**
Atomic Weight K = **39.0983 ± 0.0001 g**

Results

Results	40(a)/36(a) ± 2σ		40(r)/39(k) ± 2σ		Age ± 2σ (Ma)		MSWD	39Ar(k) (%,n)	K/Ca ± 2σ
**Age Plateau**			16.14811	± 0.05336<br>± 0.33%	**49.58**	**± 0.18**<br>**± 0.37%**	0.70<br>82%	41.06<br>19	0.115 ± 0.007
				Full External Error	± 1.13		1.67	2σ Confidence Limit	
				Analytical Error	± 0.16		1.0000	Error Magnification	
**Total Fusion Age**			15.12595	± 0.03330<br>± 0.22%	**46.49**	**± 0.13**<br>**± 0.28%**		34	0.095 ± 0.003
				Full External Error	± 1.05				
				Analytical Error	± 0.10				
**Normal Isochron**	**363.22**	**± 11.37**<br>**± 3.13%**	16.10678	± 0.06125<br>± 0.38%	**49.46**	**± 0.21**<br>**± 0.42%**	0.79<br>71%	41.06<br>19	
				Full External Error	± 1.13		1.69	2σ Confidence Limit	
				Analytical Error	± 0.19		1.0000	Error Magnification	
**Inverse Isochron**	**360.69**	**± 11.38**<br>**± 3.15%**	16.14833	± 0.06033<br>± 0.37%	**49.58**	**± 0.20**<br>**± 0.41%**	0.76<br>74%	41.06<br>19	
				Full External Error	± 1.13		1.69	2σ Confidence Limit	
				Analytical Error	± 0.18		1.0000	Error Magnification	
							39%	Spreading Factor	

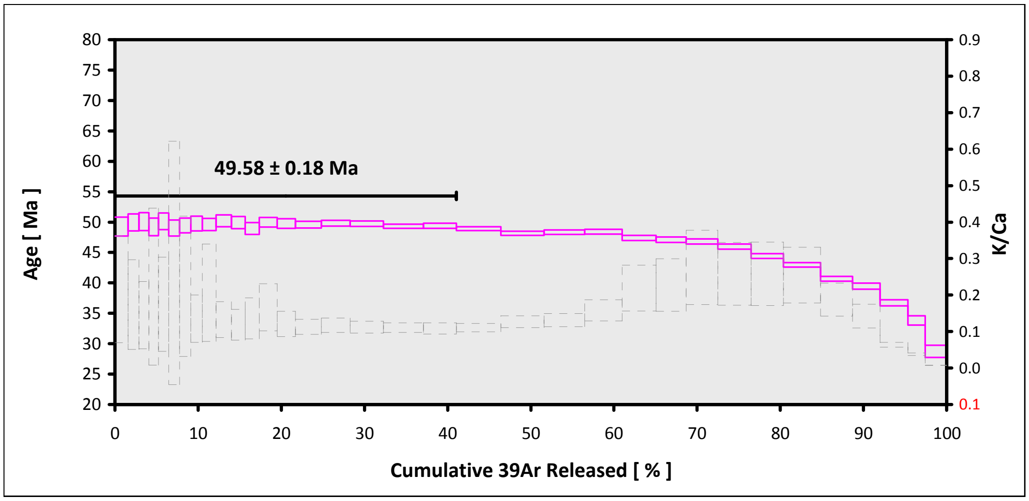

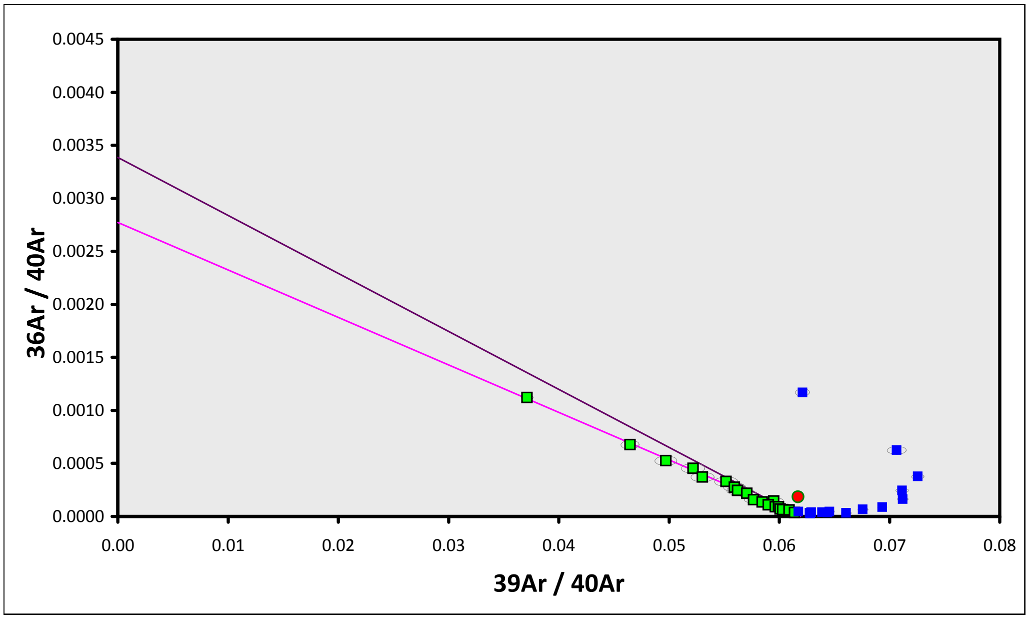

RR1310-D16-35 > Plagioclase > RURUTU (13-INT-08)
TUVALU > RURUTU HOTSPOT
14-OSU-02 (2A15-14) > Incremental Heating > Kevin Konrad

Information on Analysis and Constants Used in Calculations

Project = **RURUTU (13-INT-08)**
Sample = **RR1310-D16-35**
Material = **Plagioclase**
Location = **Rurutu Hotspot**
Region = **Tuvalu**
Analyst = **Kevin Konrad**
Irradiation = **14-OSU-02 (2A15-14)**
Position = **X: 0 | Y: 0 | Z/H: 20.6 mm**
FCT-NM Age = **28.201 ± 0.023 Ma**
FCT-NM Reference = **Kuiper et al. (2008)**
FCT-NM 40Ar/39Ar Ratio = **8.81895 ± 0.00838**
FCT-NM J-value = **0.00178223 ± 0.00000169**
Air Shot 40Ar/36Ar = **303.8810 ± 0.4163**
Air Shot MDF = **0.99309612 ± 0.00066636 (LIN)**
Experiment Type = **Incremental Heating**
Extraction Method = **Bulk Laser Heating**
Heating = **0 sec**
Isolation = **6.00 min**
Instrument = **ARGUS-VI-D**
Preferred Age = **Plateau Age**
Age Classification = **Eruption Age**
IGSN = Undefined
Rock Class = Undefined
Lithology = **Basalt**
Lat-Lon = Undefined - Undefined
Age Equations = **Min et al. (2000)**
Negative Intensities = **Allowed**
Collector Calibrations = **40Ar 36Ar**
Decay 40K = **5.530 ± 0.048 E-10 1/a**
Decay 39Ar = **2.940 ± 0.016 E-07 1/h**
Decay 37Ar = **8.230 ± 0.012 E-04 1/h**
Decay 36Cl = **2.257 ± 0.015 E-06 1/a**
Decay 40K(EC,β^+) = **0.580 ± 0.009 E-10 1/a**
Decay 40K(β^-) = **4.950 ± 0.043 E-10 1/a**
Atmospheric 40/36(a) = **295.50**
Atmospheric 38/36(a) = **0.1869**
Production 39/37(ca) = **0.0006756 ± 0.0000089**
Production 38/37(ca) = **0.0000718 ± 0.0000092**
Production 36/37(ca) = **0.0002663 ± 0.0000004**
Production 40/39(k) = **0.003823 ± 0.000102**
Production 38/39(k) = **0.012031 ± 0.000019**
Production 36/38(cl) = **262.80 ± 1.71**
Scaling Ratio K/Ca = **0.430**
Abundance Ratio 40K/K = **1.1700 ± 0.0100 E-04**
Atomic Weight K = **39.0983 ± 0.0001 g**

Results	40(a)/36(a) ± 2σ		40(r)/39(k) ± 2σ		Age ± 2σ (Ma)		MSWD	39Ar(k) (%,n)	K/Ca ± 2σ
**Age Plateau**			14.65584	± 0.15374	**46.63**	**± 0.49**	0.26	64.59	0.0249 ± 0.0012
Overestimated				± 1.05%		**± 1.05%**	99%	11	
					Full External Error	± 1.16	1.89	2σ Confidence Limit	
					Analytical Error	± 0.48	1.0000	Error Magnification	
**Total Fusion Age**			15.04109	± 0.15875	**47.84**	**± 0.51**		24	0.0245 ± 0.0003
				± 1.06%		**± 1.06%**			
					Full External Error	± 1.19			
					Analytical Error	± 0.50			
**Normal Isochron**	**232.30**	**± 190.27**	14.76616	± 0.36841	**46.97**	**± 1.16**	0.24	64.59	
Overestimated		**± 81.91%**		± 2.49%		**± 2.47%**	99%	11	
					Full External Error	± 1.57	1.94	2σ Confidence Limit	
					Analytical Error	± 1.16	1.0000	Error Magnification	
**Inverse Isochron**	**232.51**	**± 163.86**	14.76932	± 0.36859	**46.98**	**± 1.16**	0.24	64.59	
Overestimated		**± 70.47%**		± 2.50%		**± 2.47%**	99%	11	
					Full External Error	± 1.57	1.94	2σ Confidence Limit	
					Analytical Error	± 1.16	1.0000	Error Magnification	
							6%	Spreading Factor	

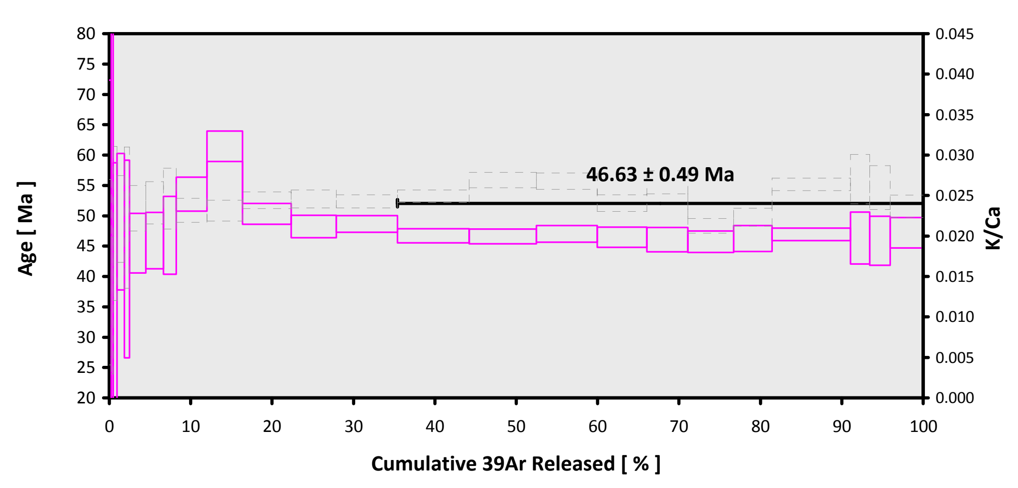

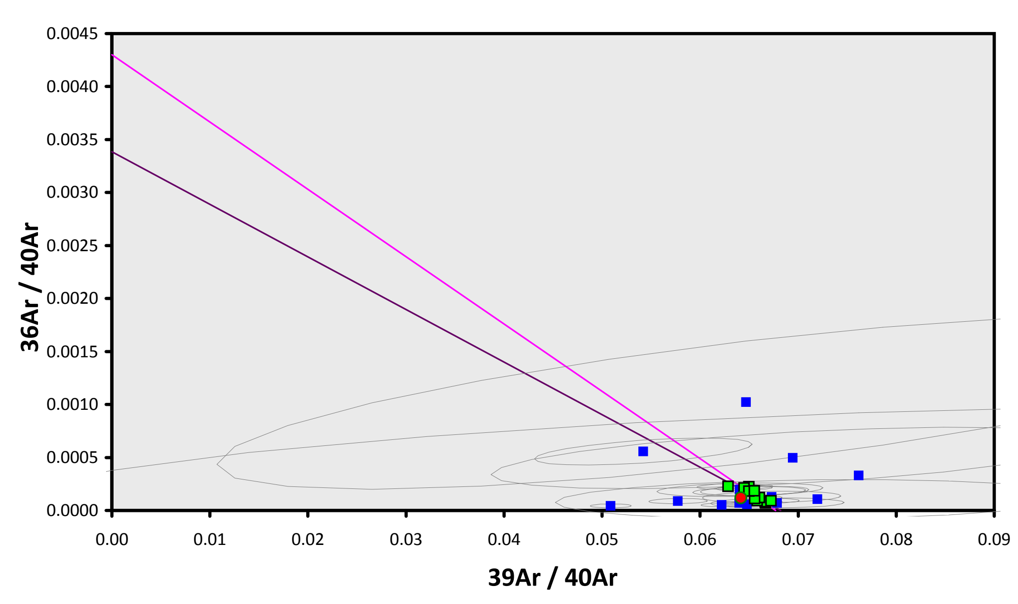

RR1310-D18-07 > Hornblende > RURUTU (13-INT-08)
TUVALU > RURUTU HOTSPOT
14-OSU-02 (2A16-14) > Incremental Heating > Kevin Konrad

Information on Analysis and Constants Used in Calculations

Project = **RURUTU (13-INT-08)**
Sample = **RR1310-D18-07**
Material = **Hornblende**
Location = **Rurutu Hotspot**
Region = **Tuvalu**
Analyst = **Kevin Konrad**
Irradiation = **14-OSU-02 (2A16-14)**
Position = **X: 0 | Y: 0 | Z/H: 21.4 mm**
FCT-NM Age = **28.201 ± 0.023 Ma**
FCT-NM Reference = **Kuiper et al (2008)**
FCT-NM 40Ar/39Ar Ratio = **8.81817 ± 0.00838**
FCT-NM J-value = **0.00178239 ± 0.00000169**
Air Shot 40Ar/36Ar = **303.9880 ± 0.4165**
Air Shot MDF = **0.99301044 ± 0.00066612 (LIN)**
Experiment Type = **Incremental Heating**
Extraction Method = **Bulk Laser Heating**
Heating = **77 sec**
Isolation = **6.00 min**
Instrument = **ARGUS-VI-D**
Preferred Age = **Plateau Age**
Age Classification = **Eruption Age**
IGSN = Undefined
Rock Class = Undefined
Lithology = **Basalt**
Lat-Lon = Undefined - Undefined
Age Equations = **Min et al. (2000)**
Negative Intensities = **Allowed**
Collector Calibrations = **40Ar 36Ar**
Decay 40K = **5.530 ± 0.048 E-10 1/a**
Decay 39Ar = **2.940 ± 0.016 E-07 1/h**
Decay 37Ar = **8.230 ± 0.012 E-04 1/h**
Decay 36Cl = **2.257 ± 0.015 E-06 1/a**
Decay 40K(EC,β^+) = **0.580 ± 0.009 E-10 1/a**
Decay 40K(β^-) = **4.950 ± 0.043 E-10 1/a**
Atmospheric 40/36(a) = **295.50**
Atmospheric 38/36(a) = **0.1869**
Production 39/37(ca) = **0.0006756 ± 0.0000089**
Production 38/37(ca) = **0.0000718 ± 0.0000092**
Production 36/37(ca) = **0.0002663 ± 0.0000004**
Production 40/39(k) = **0.003823 ± 0.000102**
Production 38/39(k) = **0.012031 ± 0.000019**
Production 36/38(cl) = **262.80 ± 1.71**
Scaling Ratio K/Ca = **0.430**
Abundance Ratio 40K/K = **1.1700 ± 0.0100 E-04**
Atomic Weight K = **39.0983 ± 0.0001 g**

Results

Results	40(a)/36(a) ± 2σ	40(r)/39(k) ± 2σ	Age ± 2σ (Ma)	MSWD	39Ar(k) (%,n)	K/Ca ± 2σ
**Age Plateau**		15.37378 ± 0.02451 ± 0.16%	**48.88 ± 0.12 ± 0.24%**	1.08 37%	94.92 14	0.066 ± 0.001
		Full External Error	± 1.10	1.78	2σ Confidence Limit	
		Analytical Error	± 0.08	1.0391	Error Magnification	
**Total Fusion Age**		15.38690 ± 0.02835 ± 0.18%	**48.93 ± 0.13 ± 0.26%**		16	0.066 ± 0.001
		Full External Error	± 1.10			
		Analytical Error	± 0.09			
**Normal Isochron** No Convergence	**248.75 ± 50.22 ± 20.19%**	15.47539 ± 0.10280 ± 0.66%	**49.20 ± 0.34 ± 0.68%**	2.21 1%	94.92 14	
		Full External Error	± 1.15	1.82	2σ Confidence Limit	
		Analytical Error	± 0.32	1.4877	Error Magnification	
**Inverse Isochron**	**308.32 ± 37.18 ± 12.06%**	15.34889 ± 0.07767 ± 0.51%	**48.81 ± 0.26 ± 0.53%**	1.10 35%	94.92 14	
		Full External Error	± 1.12	1.82	2σ Confidence Limit	
		Analytical Error	± 0.24	1.0487	Error Magnification	
				10%	Spreading Factor	

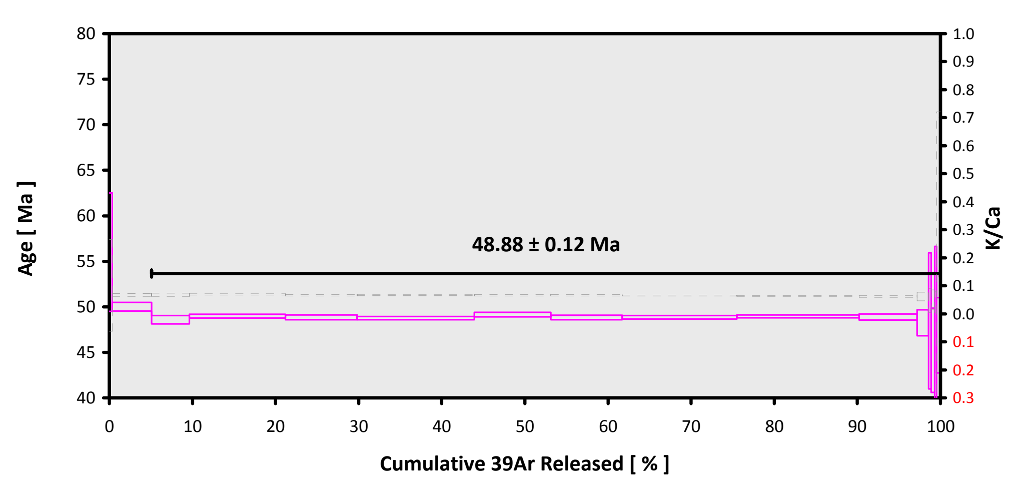

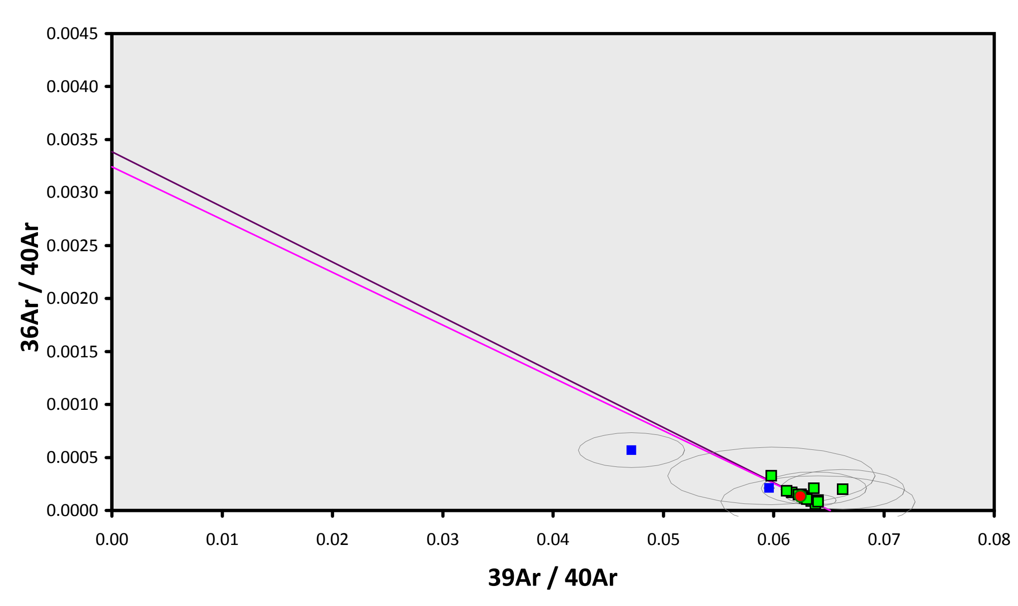

RR1310-D18-23 > Hornblende > RURUTU (13-INT-08)
TUVALU > RURUTU HOTSPOT
14-OSU-02 (2A48-14) > Incremental Heating > Kevin Konrad

Information on Analysis and Constants Used in Calculations

Project = **RURUTU (13-INT-08)**
Sample = **RR1310-D18-23**
Material = **Hornblende**
Location = **Rurutu Hotspot**
Region = **Tuvalu**
Analyst = **Kevin Konrad**
Irradiation = **14-OSU-02 (2A48-14)**
Position = **X: 0 | Y: 0 | Z/H: 57.5 mm**
FCT-NM Age = **28.201 ± 0.023 Ma**
FCT-NM Reference = **Kuiper et al. (2008)**
FCT-NM 40Ar/39Ar Ratio = **9.03974 ± 0.00841**
FCT-NM J-value = **0.00173870 ± 0.00000162**
Air Shot 40Ar/36Ar = **303.9910 ± 0.4165**
Air Shot MDF = **0.99300804 ± 0.00066611 (LIN)**
Experiment Type = **Incremental Heating**
Extraction Method = **Bulk Laser Heating**
Heating = **77 sec**
Isolation = **6.00 min**
Instrument = **ARGUS-VI-D**
Preferred Age = **Plateau Age**
Age Classification = **Eruption Age**
IGSN = Undefined
Rock Class = Undefined
Lithology = **Basalt**
Lat-Lon = Undefined - Undefined
Age Equations = **Min et al. (2000)**
Negative Intensities = **Allowed**
Collector Calibrations = **40Ar 36Ar**
Decay 40K = **5.530 ± 0.048 E-10 1/a**
Decay 39Ar = **2.940 ± 0.016 E-07 1/h**
Decay 37Ar = **8.230 ± 0.012 E-04 1/h**
Decay 36Cl = **2.257 ± 0.015 E-06 1/a**
Decay 40K(EC,β^+) = **0.580 ± 0.009 E-10 1/a**
Decay 40K(β^-) = **4.950 ± 0.043 E-10 1/a**
Atmospheric 40/36(a) = **295.50**
Atmospheric 38/36(a) = **0.1869**
Production 39/37(ca) = **0.0006756 ± 0.0000089**
Production 38/37(ca) = **0.0000718 ± 0.0000092**
Production 36/37(ca) = **0.0002663 ± 0.0000004**
Production 40/39(k) = **0.003823 ± 0.000102**
Production 38/39(k) = **0.012031 ± 0.000019**
Production 36/38(cl) = **262.80 ± 1.71**
Scaling Ratio K/Ca = **0.430**
Abundance Ratio 40K/K = **1.1700 ± 0.0100 E-04**
Atomic Weight K = **39.0983 ± 0.0001 g**

Results

Results	40(a)/36(a) ± 2σ		40(r)/39(k) ± 2σ		Age ± 2σ (Ma)		MSWD	39Ar(k) (%,n)	K/Ca ± 2σ
**Age Plateau**			15.77483	± 0.02753<br>± 0.17%	**48.93**	**± 0.12<br>± 0.25%**	1.44<br>18%	85.51<br>8	0.066 ± 0.001
				Full External Error	± 1.10		2.07	2σ Confidence Limit	
				Analytical Error	± 0.08		1.1997	Error Magnification	
**Total Fusion Age**			15.78027	± 0.02483<br>± 0.16%	**48.95**	**± 0.12<br>± 0.24%**		13	0.067 ± 0.001
				Full External Error	± 1.10				
				Analytical Error	± 0.08				
**Normal Isochron**	**234.97**	**± 101.16<br>± 43.05%**	15.80936	± 0.06609<br>± 0.42%	**49.04**	**± 0.22<br>± 0.45%**	1.41<br>20%	85.51<br>8	
				Full External Error	± 1.12		2.15	2σ Confidence Limit	
				Analytical Error	± 0.20		1.1895	Error Magnification	
**Inverse Isochron**<br>Clustered Points	**248.01**	**± 95.56<br>± 38.53%**	15.80340	± 0.06801<br>± 0.43%	**49.02**	**± 0.23<br>± 0.46%**	1.49<br>18%	85.51<br>8	
				Full External Error	± 1.12		2.15	2σ Confidence Limit	
				Analytical Error	± 0.21		1.2226	Error Magnification	
							1%	Spreading Factor	

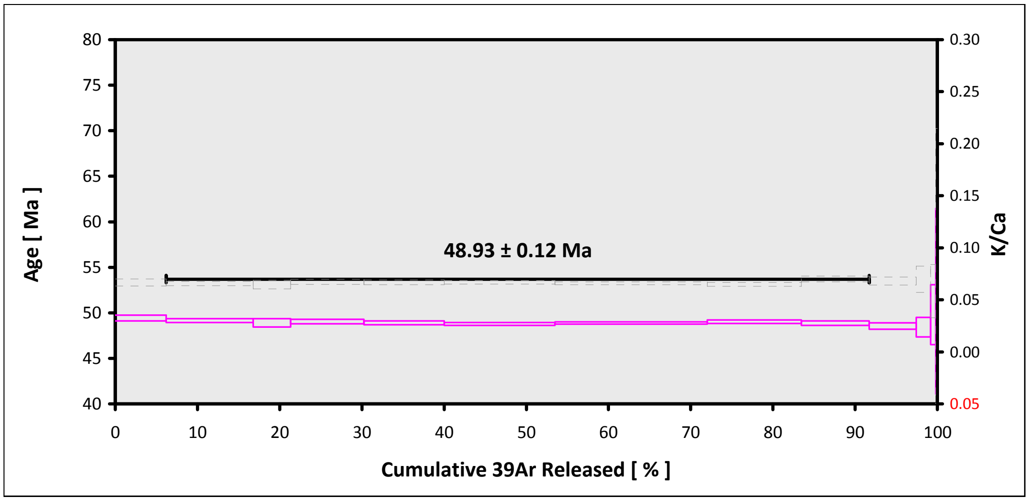

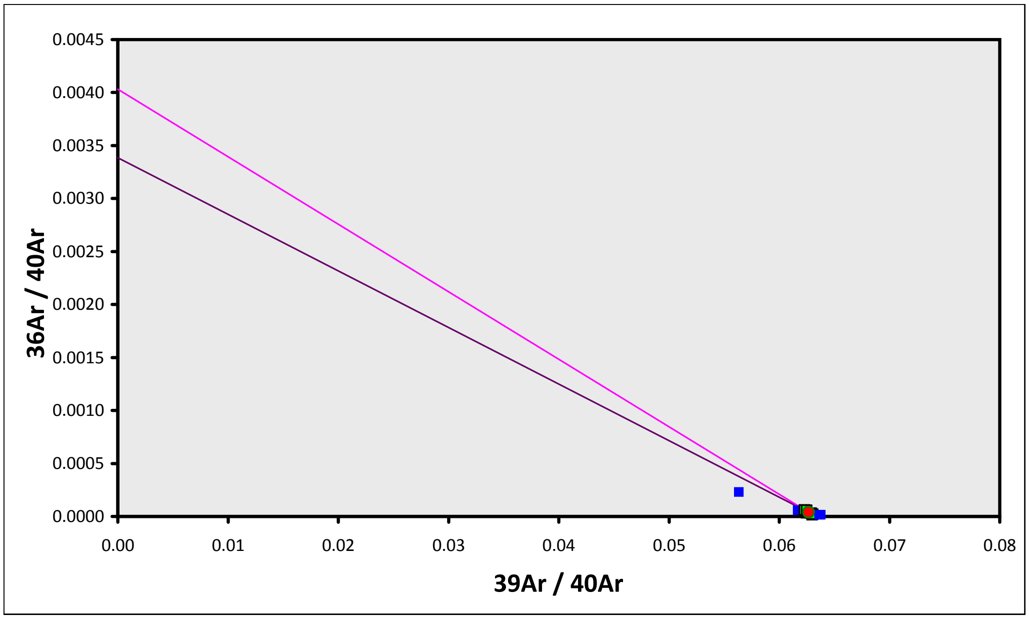

RR1310-D22-29 > Groundmass > KONRAD (13-INT-08)
TUVALU > RURUTU HOTSPOT
15-OSU-04 (4A18-15) > Incremental Heating > Kevin Konrad

Information on Analysis and Constants Used in Calculations

Project = **KONRAD (13-INT-08)**
Sample = **RR1310-D22-29**
Material = **Groundmass**
Location = **Rurutu Hotspot**
Region = **Tuvalu**
Analyst = **Kevin Konrad**
Irradiation = **15-OSU-04 (4A18-15)**
Position = **X: 0 | Y: 0 | Z/H: 25.43 mm**
FCT-NM Age = **28.201 ± 0.023 Ma**
FCT-NM Reference = **Kuiper et al (2008)**
FCT-NM 40Ar/39Ar Ratio = **8.90893 ± 0.01515**
FCT-NM J-value = **0.00176423 ± 0.00000300**
Air Shot 40Ar/36Ar = **304.4790 ± 0.5602**
Air Shot MDF = **0.99261804 ± 0.00072892 (LIN)**
Experiment Type = **Incremental Heating**
Extraction Method = **Bulk Laser Heating**
Heating = **77 sec**
Isolation = **3.00 min**
Instrument = **ARGUS-VI-D**
Preferred Age = **Plateau Age**
Age Classification = **Eruption Age**
IGSN = Undefined
Rock Class = Undefined
Lithology = **Basalt**
Lat-Lon = Undefined - Undefined
Age Equations = **Min et al. (2000)**
Negative Intensities = **Allowed**
Collector Calibrations = **36Ar**
Decay 40K = **5.530 ± 0.048 E-10 1/a**
Decay 39Ar = **2.940 ± 0.016 E-07 1/h**
Decay 37Ar = **8.230 ± 0.012 E-04 1/h**
Decay 36Cl = **2.257 ± 0.015 E-06 1/a**
Decay 40K(EC,β^+) = **0.580 ± 0.009 E-10 1/a**
Decay 40K(β^-) = **4.950 ± 0.043 E-10 1/a**
Atmospheric 40/36(a) = **295.50**
Atmospheric 38/36(a) = **0.1869**
Production 39/37(ca) = **0.0006756 ± 0.0000089**
Production 38/37(ca) = **0.0000718 ± 0.0000092**
Production 36/37(ca) = **0.0002663 ± 0.0000004**
Production 40/39(k) = **0.003823 ± 0.000102**
Production 38/39(k) = **0.012031 ± 0.000019**
Production 36/38(cl) = **262.80 ± 1.71**
Scaling Ratio K/Ca = **0.430**
Abundance Ratio 40K/K = **1.1700 ± 0.0100 E-04**
Atomic Weight K = **39.0983 ± 0.0001 g**

Results

Results	40(a)/36(a) ± 2σ		40(r)/39(k) ± 2σ		Age ± 2σ (Ma)		MSWD	39Ar(k) (%,n)	K/Ca ± 2σ
**Age Plateau**			14.63342	± 0.07680	**46.09**	**± 0.28**	4.27	33.83	0.0825 ± 0.0072
Error Mean				± 0.52%		**± 0.62%**	0%	12	
					Full External Error	± 1.07	1.85	2σ Confidence Limit	
					Analytical Error	± 0.24	2.0657	Error Magnification	
**Total Fusion Age**			13.60193	± 0.02293	**42.88**	**± 0.16**		31	0.0214 ± 0.0001
				± 0.17%		**± 0.38%**			
					Full External Error	± 0.98			
					Analytical Error	± 0.07			
**Normal Isochron**	**323.21**	**± 55.80**	14.55812	± 0.12201	**45.86**	**± 0.41**	4.97	33.83	
No Convergence		**± 17.27%**		± 0.84%		**± 0.89%**	0%	12	
					Full External Error	± 1.11	1.89	2σ Confidence Limit	
					Analytical Error	± 0.38	2.2296	Error Magnification	
**Inverse Isochron**	**304.46**	**± 52.53**	14.62222	± 0.11837	**46.06**	**± 0.40**	4.67	33.83	
Error Chron		**± 17.25%**		± 0.81%		**± 0.87%**	0%	12	
					Full External Error	± 1.11	1.89	2σ Confidence Limit	
					Analytical Error	± 0.37	2.1619	Error Magnification	
							16%	Spreading Factor	

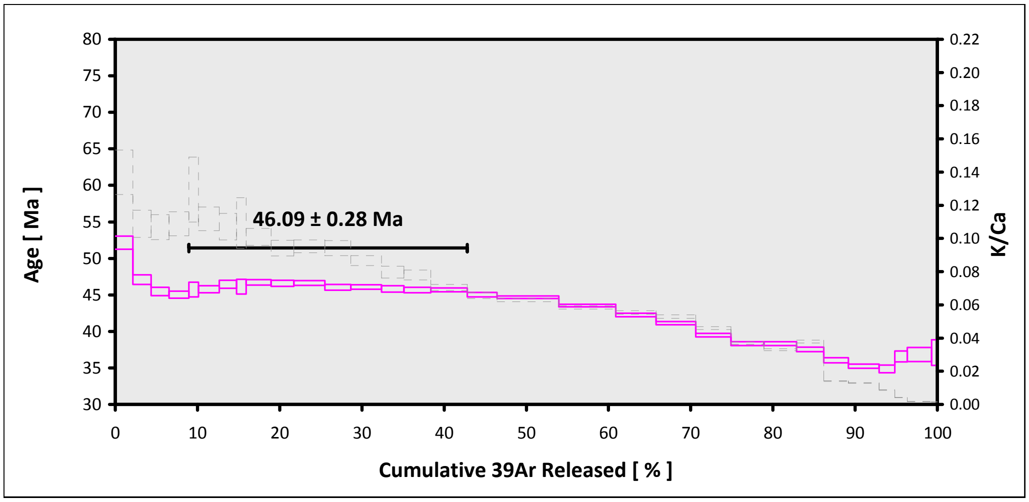

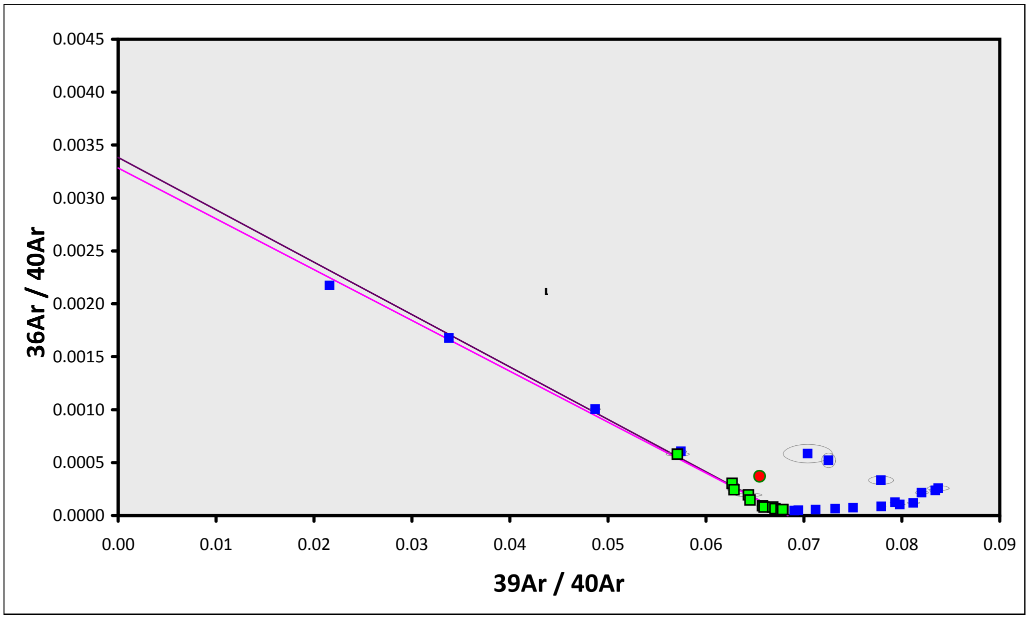

RR1310-D24-04 > Groundmass > RURUTU (13-INT-08)
TUVALU > RURUTU HOTSPOT
14-OSU-02 (2A30-14) > Incremental Heating > Kevin Konrad

Information on Analysis and Constants Used in Calculations

Project = **RURUTU (13-INT-08)**
Sample = **RR1310-D24-04**
Material = **Groundmass**
Location = **Rurutu Hotspot**
Region = **Tuvalu**
Analyst = **Kevin Konrad**
Irradiation = **14-OSU-02 (2A30-14)**
Position = **X: 0 | Y: 0 | Z/H: 35.6 mm**
FCT-NM Age = **28.201 ± 0.023 Ma**
FCT-NM Reference = **Kuiper et al. (2008)**
FCT-NM 40Ar/39Ar Ratio = **8.84537 ± 0.00840**
FCT-NM J-value = **0.00177691 ± 0.00000169**
Air Shot 40Ar/36Ar = **303.8610 ± 0.4467**
Air Shot MDF = **0.99311214 ± 0.00067891 (LIN)**
Experiment Type = **Incremental Heating**
Extraction Method = **Bulk Laser Heating**
Heating = **77 sec**
Isolation = **10.00 min**
Instrument = **ARGUS-VI-D**
Preferred Age = **Plateau Age**
Age Classification = **Eruption Age**
IGSN = Undefined
Rock Class = Undefined
Lithology = **Basalt**
Lat-Lon = Undefined - Undefined
Age Equations = **Min et al. (2000)**
Negative Intensities = **Allowed**
Collector Calibrations = **40Ar 36Ar**
Decay 40K = **5.530 ± 0.048 E-10 1/a**
Decay 39Ar = **2.940 ± 0.016 E-07 1/h**
Decay 37Ar = **8.230 ± 0.012 E-04 1/h**
Decay 36Cl = **2.257 ± 0.015 E-06 1/a**
Decay 40K(EC,β^+) = **0.580 ± 0.009 E-10 1/a**
Decay 40K(β^-) = **4.950 ± 0.043 E-10 1/a**
Atmospheric 40/36(a) = **499.40 ± 6.49**
Atmospheric 38/36(a) = **0.1869**
Production 39/37(ca) = **0.0006756 ± 0.0000089**
Production 38/37(ca) = **0.0000718 ± 0.0000092**
Production 36/37(ca) = **0.0002663 ± 0.0000004**
Production 40/39(k) = **0.003823 ± 0.000102**
Production 38/39(k) = **0.012031 ± 0.000019**
Production 36/38(cl) = **262.80 ± 1.71**
Scaling Ratio K/Ca = **0.430**
Abundance Ratio 40K/K = **1.1700 ± 0.0100 E-04**
Atomic Weight K = **39.0983 ± 0.0001 g**

Results

Results	40(a)/36(a) ± 2σ	40(r)/39(k) ± 2σ	Age ± 2σ (Ma)	MSWD	39Ar(k) (%,n)	K/Ca ± 2σ
**Age Plateau**<br>Error Mean		14.93860 ± 0.01888<br>± 0.13%	**47.37 ± 0.11**<br>**± 0.23%**<br>Full External Error ± 1.07<br>Analytical Error ± 0.06	2.18<br>0%<br>1.59<br>1.4776	62.49<br>24<br>2σ Confidence Limit<br>Error Magnification	0.322 ± 0.009
**Total Fusion Age**		14.41228 ± 0.01121<br>± 0.08%	**45.73 ± 0.09**<br>**± 0.20%**<br>Full External Error ± 1.03<br>Analytical Error ± 0.04		36	0.178 ± 0.002
**Normal Isochron**<br>Error Chron	**502.10 ± 13.48**<br>**± 2.68%**	14.92513 ± 0.02366<br>± 0.16%	**47.33 ± 0.12**<br>**± 0.24%**<br>Full External Error ± 1.07<br>Analytical Error ± 0.07	2.35<br>0%<br>1.60<br>1.5315	62.49<br>24<br>2σ Confidence Limit<br>Error Magnification	
**Inverse Isochron**<br>Error Chron	**499.36 ± 13.45**<br>**± 2.69%**	14.93883 ± 0.02340<br>± 0.16%	**47.38 ± 0.12**<br>**± 0.24%**<br>Full External Error ± 1.07<br>Analytical Error ± 0.07	2.35<br>0%<br>1.60<br>1.5332<br>18%	62.49<br>24<br>2σ Confidence Limit<br>Error Magnification<br>Spreading Factor	

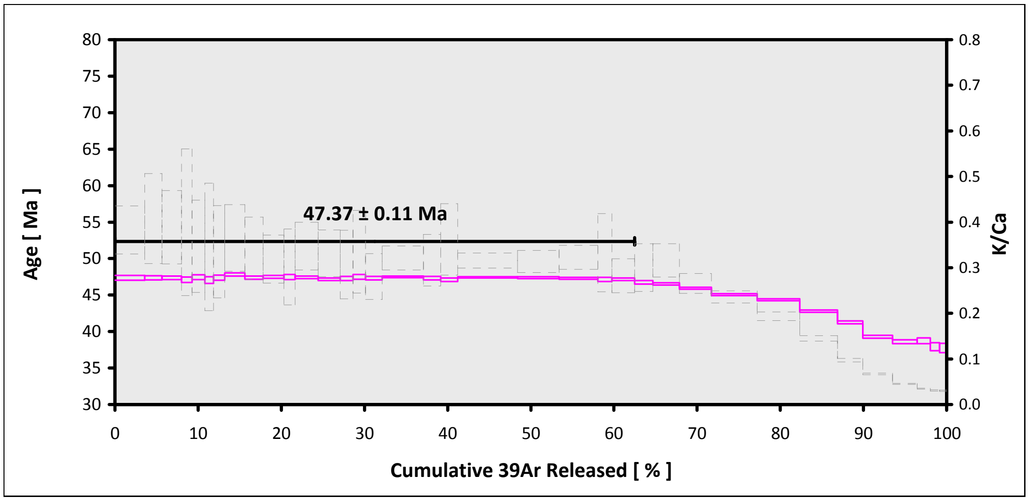

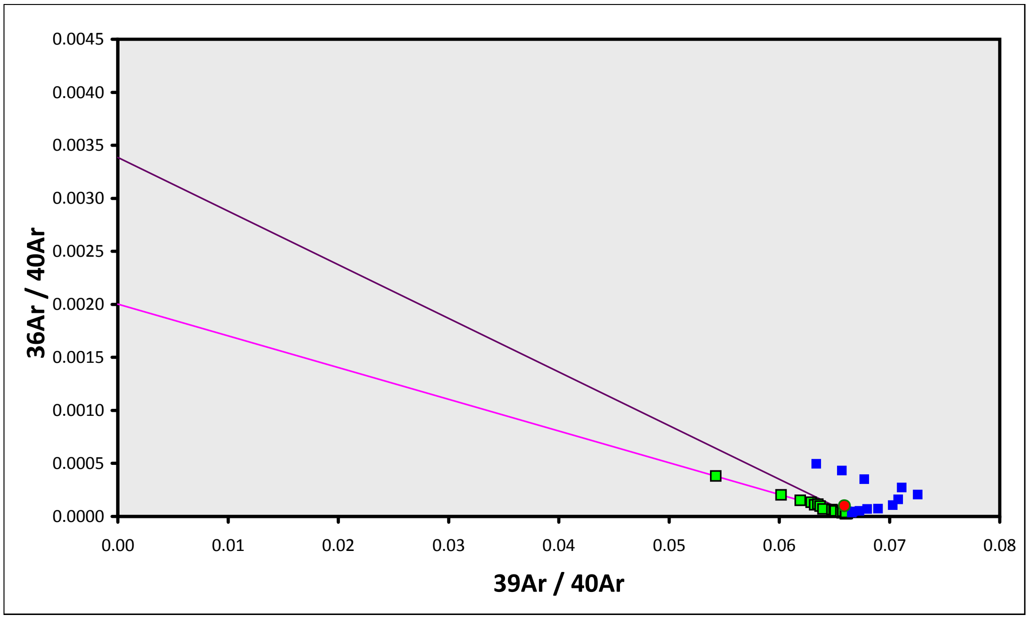

RR1310-D24-11 > Groundmass > RURUTU (13-INT-08)
TUVALU > RURUTU HOTSPOT
14-OSU-02 (2A52-14) > Incremental Heating > Kevin Konrad

Information on Analysis and Constants Used in Calculations

Project = **RURUTU (13-INT-08)**
Sample = **RR1310-D24-11**
Material = **Groundmass**
Location = **Rurutu Hotspot**
Region = **Tuvalu**
Analyst = **Kevin Konrad**
Irradiation = **14-OSU-02 (2A52-14)**
Position = **X: 0 | Y: 0 | Z/H: 61.7 mm**
FCT-NM Age = **28.201 ± 0.023 Ma**
FCT-NM Reference = **Kuiper et al. (2008)**
FCT-NM 40Ar/39Ar Ratio = **9.09815 ± 0.00837**
FCT-NM J-value = **0.00172754 ± 0.00000159**
Air Shot 40Ar/36Ar = **303.7710 ± 0.4557**
Air Shot MDF = **0.99318427 ± 0.00068299 (LIN)**
Experiment Type = **Incremental Heating**
Extraction Method = **Bulk Laser Heating**
Heating = **77 sec**
Isolation = **10.00 min**
Instrument = **ARGUS-VI-D**
Preferred Age = **Plateau Age**
Age Classification = **Eruption Age**
IGSN = Undefined
Rock Class = Undefined
Lithology = **Basalt**
Lat-Lon = Undefined - Undefined
Age Equations = **Min et al. (2000)**
Negative Intensities = **Allowed**
Collector Calibrations = **40Ar 36Ar**
Decay 40K = **5.530 ± 0.048 E-10 1/a**
Decay 39Ar = **2.940 ± 0.016 E-07 1/h**
Decay 37Ar = **8.230 ± 0.012 E-04 1/h**
Decay 36Cl = **2.257 ± 0.015 E-06 1/a**
Decay 40K(EC,β^+) = **0.580 ± 0.009 E-10 1/a**
Decay 40K(β^-) = **4.950 ± 0.043 E-10 1/a**
Atmospheric 40/36(a) = **415.20 ± 51.48**
Atmospheric 38/36(a) = **0.1869**
Production 39/37(ca) = **0.0006756 ± 0.0000089**
Production 38/37(ca) = **0.0000718 ± 0.0000092**
Production 36/37(ca) = **0.0002663 ± 0.0000004**
Production 40/39(k) = **0.003823 ± 0.000102**
Production 38/39(k) = **0.012031 ± 0.000019**
Production 36/38(cl) = **262.80 ± 1.71**
Scaling Ratio K/Ca = **0.430**
Abundance Ratio 40K/K = **1.1700 ± 0.0100 E-04**
Atomic Weight K = **39.0983 ± 0.0001 g**

Results

Results	40(a)/36(a) ± 2σ		40(r)/39(k) ± 2σ		Age ± 2σ (Ma)		MSWD	39Ar(k) (%,n)	K/Ca ± 2σ
**Age Plateau**			15.62452	± 0.05476<br>± 0.35%	**48.16**	**± 0.19**<br>**± 0.39%**	0.71<br>85%	66.10<br>27	0.389 ± 0.042
					Full External Error	± 1.10	1.55	2σ Confidence Limit	
					Analytical Error	± 0.17	1.0000	Error Magnification	
**Total Fusion Age**			15.38644	± 0.11445<br>± 0.74%	**47.44**	**± 0.36**<br>**± 0.76%**		36	0.214 ± 0.005
					Full External Error	± 1.12			
					Analytical Error	± 0.35			
**Normal Isochron**<br>No Convergence	**392.74**	**± 15.09**<br>**± 3.84%**	15.61702	± 0.09847<br>± 0.63%	**48.14**	**± 0.31**<br>**± 0.65%**	16.32<br>0%	66.10<br>27	
					Full External Error	± 1.12	1.57	2σ Confidence Limit	
					Analytical Error	± 0.30	4.0397	Error Magnification	
**Inverse Isochron**<br>Error Chron	**386.16**	**± 14.07**<br>**± 3.64%**	15.72114	± 0.09036<br>± 0.57%	**48.46**	**± 0.29**<br>**± 0.60%**	13.89<br>0%	66.10<br>27	
					Full External Error	± 1.12	1.57	2σ Confidence Limit	
					Analytical Error	± 0.27	3.7270	Error Magnification	
							42%	Spreading Factor	

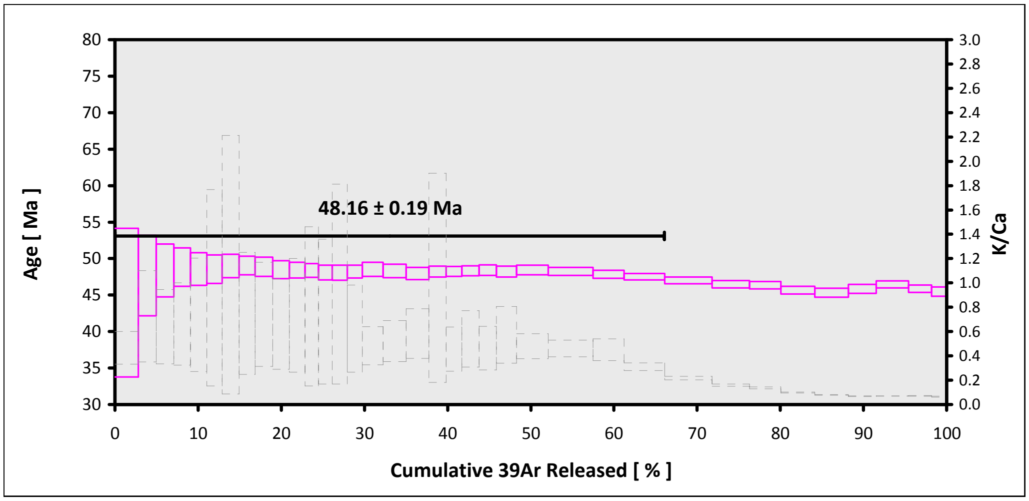

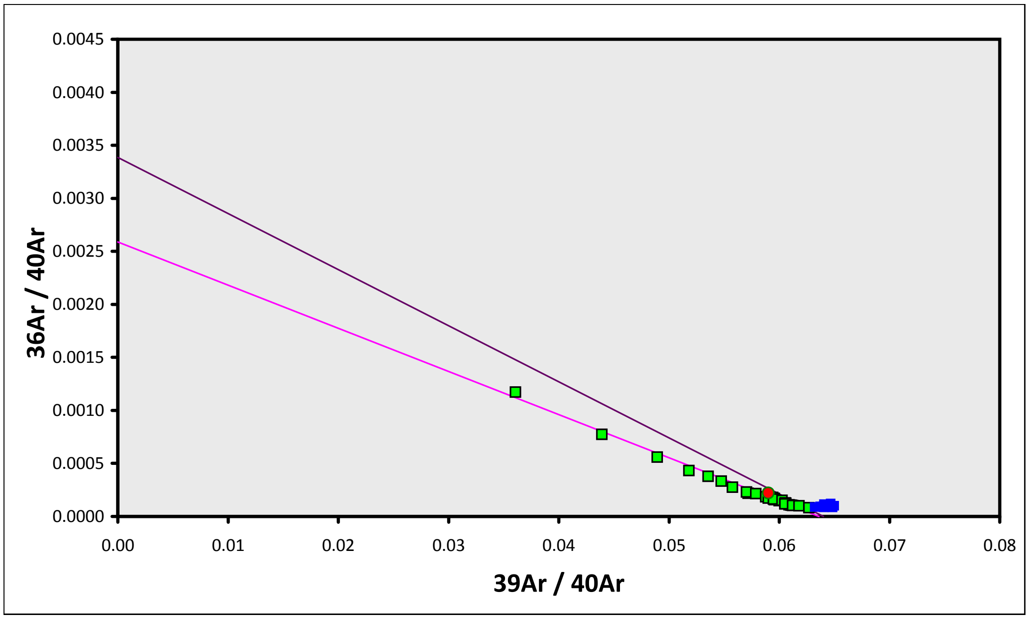

RR1310-D27-35 > Hornblende > RURUTU (13-INT-08)
SAMOA > RURUTU HOTSPOT
14-OSU-02 (2A20-14) > Incremental Heating > Kevin Konrad

Information on Analysis and Constants Used in Calculations

Project = **RURUTU (13-INT-08)**
Sample = **RR1310-D27-35**
Material = **Hornblende**
Location = **Rurutu Hotspot**
Region = **Samoa**
Analyst = **Kevin Konrad**
Irradiation = **14-OSU-02 (2A20-14)**
Position = **X: 0 | Y: 0 | Z/H: 24.3 mm**
FCT-NM Age = **28.201 ± 0.023 Ma**
FCT-NM Reference = **Kuiper et al. (2008)**
FCT-NM 40Ar/39Ar Ratio = **8.81741 ± 0.00838**
FCT-NM J-value = **0.00178254 ± 0.00000169**
Air Shot 40Ar/36Ar = **303.9890 ± 0.4165**
Air Shot MDF = **0.99300964 ± 0.00066612 (LIN)**
Experiment Type = **Incremental Heating**
Extraction Method = **Bulk Laser Heating**
Heating = **77 sec**
Isolation = **6.00 min**
Instrument = **ARGUS-VI-D**
Preferred Age = **Plateau Age**
Age Classification = **Eruption Age**
IGSN = Undefined
Rock Class = Undefined
Lithology = **Basalt**
Lat-Lon = Undefined - Undefined
Age Equations = **Min et al. (2000)**
Negative Intensities = **Allowed**
Collector Calibrations = **40Ar 36Ar**
Decay 40K = **5.530 ± 0.048 E-10 1/a**
Decay 39Ar = **2.940 ± 0.016 E-07 1/h**
Decay 37Ar = **8.230 ± 0.012 E-04 1/h**
Decay 36Cl = **2.257 ± 0.015 E-06 1/a**
Decay 40K(EC,β^+) = **0.580 ± 0.009 E-10 1/a**
Decay 40K(β^-) = **4.950 ± 0.043 E-10 1/a**
Atmospheric 40/36(a) = **295.50**
Atmospheric 38/36(a) = **0.1869**
Production 39/37(ca) = **0.0006756 ± 0.0000089**
Production 38/37(ca) = **0.0000718 ± 0.0000092**
Production 36/37(ca) = **0.0002663 ± 0.0000004**
Production 40/39(k) = **0.003823 ± 0.000102**
Production 38/39(k) = **0.012031 ± 0.000019**
Production 36/38(cl) = **262.80 ± 1.71**
Scaling Ratio K/Ca = **0.430**
Abundance Ratio 40K/K = **1.1700 ± 0.0100 E-04**
Atomic Weight K = **39.0983 ± 0.0001 g**

Results

Results	40(a)/36(a) ± 2σ	40(r)/39(k) ± 2σ	Age ± 2σ (Ma)	MSWD	39Ar(k) (%,n)	K/Ca ± 2σ
**Age Plateau**		13.26000 ± 0.26058 ± 1.97%	**42.24 ± 0.82 ± 1.95%**	0.78 68%	97.05 14	0.0028 ± 0.0014
			Full External Error ± 1.26	1.78	2σ Confidence Limit	
			Analytical Error ± 0.82	1.0000	Error Magnification	
**Total Fusion Age**		12.93077 ± 0.35829 ± 2.77%	**41.21 ± 1.13 ± 2.75%**		17	0.0060 ± 0.0002
			Full External Error ± 1.46			
			Analytical Error ± 1.13			
**Normal Isochron** No Convergence	**207.73 ± 112.10 ± 53.96%**	13.60617 ± 0.43969 ± 3.23%	**43.33 ± 1.39 ± 3.20%**	1.13 33%	97.05 14	
			Full External Error ± 1.69	1.82	2σ Confidence Limit	
			Analytical Error ± 1.38	1.0616	Error Magnification	
**Inverse Isochron**	**319.52 ± 106.01 ± 33.18%**	13.20466 ± 0.44691 ± 3.38%	**42.07 ± 1.41 ± 3.35%**	0.79 66%	97.05 14	
			Full External Error ± 1.70	1.82	2σ Confidence Limit	
			Analytical Error ± 1.41	1.0000	Error Magnification	
				25%	Spreading Factor	

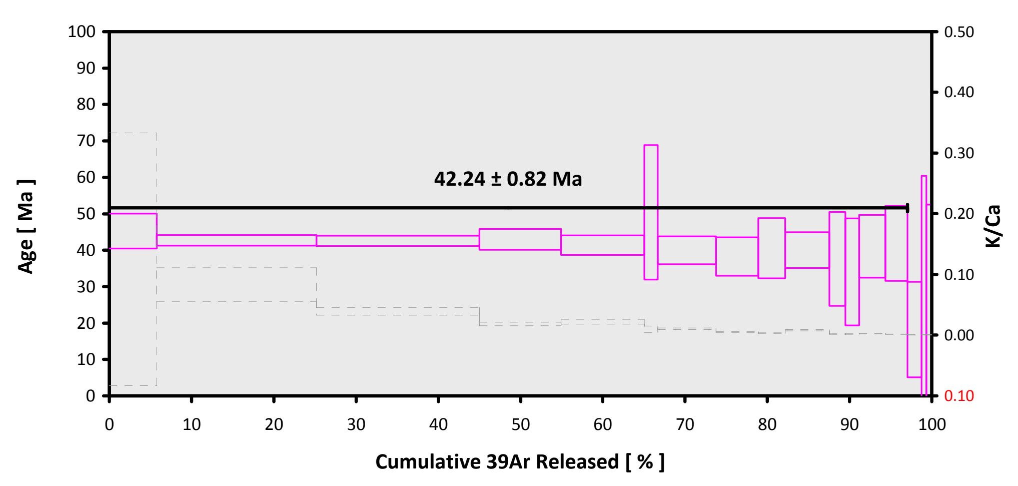

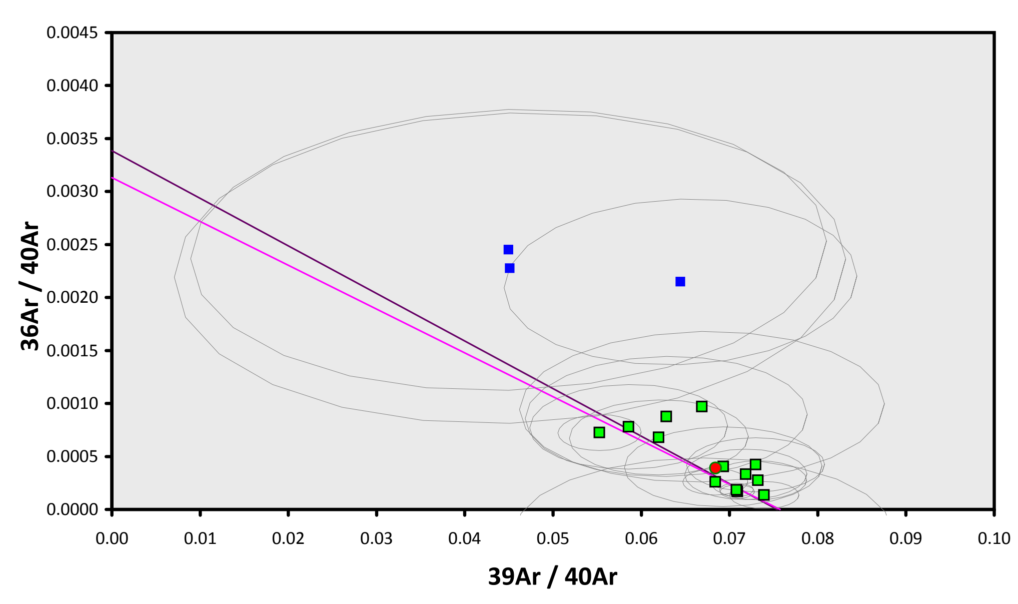

RR1310-D27-64 > Hornblende > KONRAD (13-INT-08)
PACIFIC OCEAN > SAMOA
15-OSU-06 (6A20-15) > Incremental Heating > Kevin Konrad

Information on Analysis and Constants Used in Calculations

Project = **KONRAD (13-INT-08)**
Sample = **RR1310-D27-64**
Material = **Hornblende**
Location = **Samoa**
Region = **Pacific Ocean**
Analyst = **Kevin Konrad**
Irradiation = **15-OSU-06 (6A20-15)**
Position = **X: 0 | Y: 0 | Z/H: 42.13 mm**
FCT-NM Age = **28.201 ± 0.023 Ma**
FCT-NM Reference = **Kuiper et al (2008)**
FCT-NM 40Ar/39Ar Ratio = **9.24573 ± 0.01230**
FCT-NM J-value = **0.00169996 ± 0.00000226**
Air Shot 40Ar/36Ar = **304.9400 ± 0.6068**
Air Shot MDF = **0.99225077 ± 0.00075069 (LIN)**
Experiment Type = **Incremental Heating**
Extraction Method = **Bulk Laser Heating**
Heating = **77 sec**
Isolation = **1.50 min**
Instrument = **ARGUS-VI-D**
Preferred Age = **Plateau Age**
Age Classification = **Eruption Age**
IGSN = Undefined
Rock Class = Undefined
Lithology = **Basalt**
Lat-Lon = Undefined - Undefined
Age Equations = **Min et al. (2000)**
Negative Intensities = **Allowed**
Collector Calibrations = **36Ar**
Decay 40K = **5.530 ± 0.048 E-10 1/a**
Decay 39Ar = **2.940 ± 0.016 E-07 1/h**
Decay 37Ar = **8.230 ± 0.012 E-04 1/h**
Decay 36Cl = **2.257 ± 0.015 E-06 1/a**
Decay 40K(EC,β^+) = **0.580 ± 0.009 E-10 1/a**
Decay 40K(β^-) = **4.950 ± 0.043 E-10 1/a**
Atmospheric 40/36(a) = **295.50**
Atmospheric 38/36(a) = **0.1869**
Production 39/37(ca) = **0.0006756 ± 0.0000089**
Production 38/37(ca) = **0.0000718 ± 0.0000092**
Production 36/37(ca) = **0.0002663 ± 0.0000004**
Production 40/39(k) = **0.003823 ± 0.000102**
Production 38/39(k) = **0.012031 ± 0.000019**
Production 36/38(cl) = **262.80 ± 1.71**
Scaling Ratio K/Ca = **0.430**
Abundance Ratio 40K/K = **1.1700 ± 0.0100 E-04**
Atomic Weight K = **39.0983 ± 0.0001 g**

Results

Results	40(a)/36(a) ± 2σ		40(r)/39(k) ± 2σ		Age ± 2σ (Ma)		MSWD	39Ar(k) (%,n)	K/Ca ± 2σ
**Age Plateau**			14.87308	± 0.00858<br>± 0.06%	**45.15**	**± 0.12<br>± 0.27%**	0.48<br>90%	95.84<br>11	0.099 ± 0.000
					Full External Error	± 1.02	1.89	2σ Confidence Limit	
					Analytical Error	± 0.03	1.0000	Error Magnification	
**Total Fusion Age**			14.86648	± 0.00866<br>± 0.06%	**45.13**	**± 0.12<br>± 0.27%**		19	0.099 ± 0.000
					Full External Error	± 1.02			
					Analytical Error	± 0.03			
**Normal Isochron**<br>Error Chron	**382.21**	**± 66.25<br>± 17.33%**	14.81914	± 0.03932<br>± 0.27%	**44.99**	**± 0.17<br>± 0.37%**	2.02<br>3%	95.84<br>11	
					Full External Error	± 1.02	1.94	2σ Confidence Limit	
					Analytical Error	± 0.12	1.4225	Error Magnification	
**Inverse Isochron**<br>Clustered Points	**301.07**	**± 45.10<br>± 14.98%**	14.86998	± 0.02695<br>± 0.18%	**45.14**	**± 0.14<br>± 0.32%**	0.53<br>86%	95.84<br>11	
					Full External Error	± 1.02	1.94	2σ Confidence Limit	
					Analytical Error	± 0.08	1.0000	Error Magnification	
							2%	Spreading Factor	

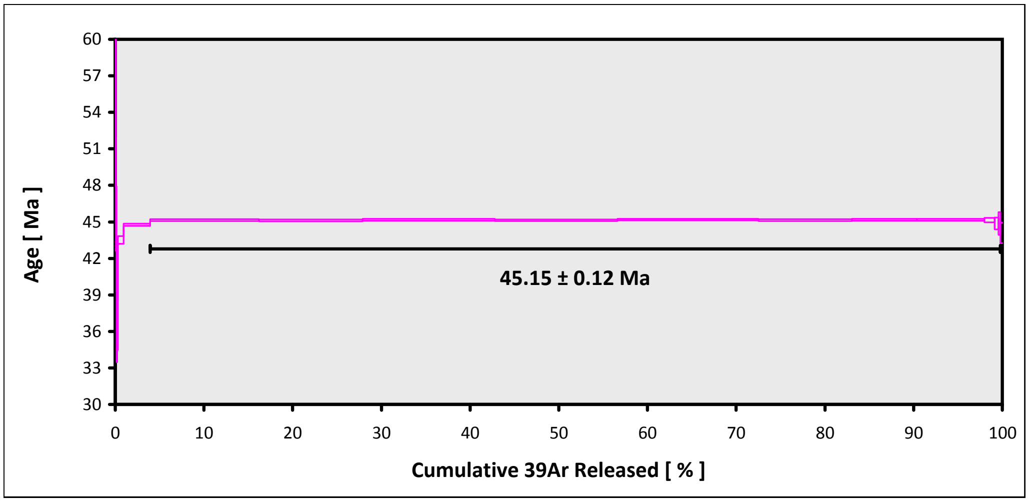

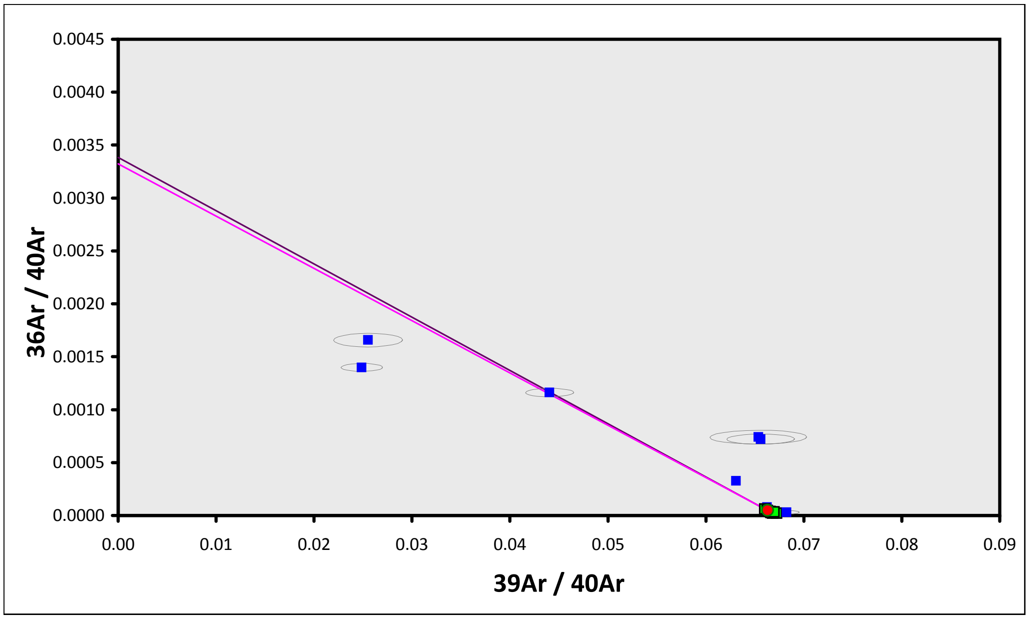

RR1310-D27-64 > Plagioclase > KONRAD (13-INT-08)
PACIFIC OCEAN > SAMOA
15-OSU-06 (6A10-15) > Incremental Heating > Kevin Konrad

Information on Analysis and Constants Used in Calculations

Project = **KONRAD (13-INT-08)**
Sample = **RR1310-D27-64**
Material = **Plagioclase**
Location = **Samoa**
Region = **Pacific Ocean**
Analyst = **Kevin Konrad**
Irradiation = **15-OSU-06 (6A10-15)**
Position = **X: 0 | Y: 0 | Z/H: 22.12 mm**
FCT-NM Age = **28.201 ± 0.023 Ma**
FCT-NM Reference = **Kuiper et al (2008)**
FCT-NM 40Ar/39Ar Ratio = **8.84550 ± 0.01230**
FCT-NM J-value = **0.00177688 ± 0.00000247**
Air Shot 40Ar/36Ar = **304.9370 ± 0.6068**
Air Shot MDF = **0.99225316 ± 0.00075069 (LIN)**
Experiment Type = **Incremental Heating**
Extraction Method = **Bulk Laser Heating**
Heating = **77 sec**
Isolation = **1.50 min**
Instrument = **ARGUS-VI-D**
Preferred Age = **Plateau Age**
Age Classification = **Eruption Age**
IGSN = Undefined
Rock Class = Undefined
Lithology = **Basalt**
Lat-Lon = Undefined - Undefined
Age Equations = **Min et al. (2000)**
Negative Intensities = **Allowed**
Collector Calibrations = **36Ar**
Decay 40K = **5.530 ± 0.048 E-10 1/a**
Decay 39Ar = **2.940 ± 0.016 E-07 1/h**
Decay 37Ar = **8.230 ± 0.012 E-04 1/h**
Decay 36Cl = **2.257 ± 0.015 E-06 1/a**
Decay 40K(EC,β^+) = **0.580 ± 0.009 E-10 1/a**
Decay 40K(β^-) = **4.950 ± 0.043 E-10 1/a**
Atmospheric 40/36(a) = **295.50**
Atmospheric 38/36(a) = **0.1869**
Production 39/37(ca) = **0.0006756 ± 0.0000089**
Production 38/37(ca) = **0.0000718 ± 0.0000092**
Production 36/37(ca) = **0.0002663 ± 0.0000004**
Production 40/39(k) = **0.003823 ± 0.000102**
Production 38/39(k) = **0.012031 ± 0.000019**
Production 36/38(cl) = **262.80 ± 1.71**
Scaling Ratio K/Ca = **0.430**
Abundance Ratio 40K/K = **1.1700 ± 0.0100 E-04**
Atomic Weight K = **39.0983 ± 0.0001 g**

Results

Results	40(a)/36(a) ± 2σ		40(r)/39(k) ± 2σ		Age ± 2σ (Ma)		MSWD	39Ar(k) (%,n)	K/Ca ± 2σ
**Age Plateau**			14.41217	± 0.02153<br>± 0.15%	**45.73**	**± 0.14<br>± 0.31%**	1.33<br>22%	17.48<br>9	0.068 ± 0.001
				Full External Error	± 1.04		2.00	2σ Confidence Limit	
				Analytical Error	± 0.07		1.1539	Error Magnification	
**Total Fusion Age**			14.07568	± 0.00770<br>± 0.05%	**44.67**	**± 0.13<br>± 0.28%**		21	0.068 ± 0.000
				Full External Error	± 1.01				
				Analytical Error	± 0.02				
**Normal Isochron**	**302.57**	**± 93.90<br>± 31.04%**	14.40326	± 0.09906<br>± 0.69%	**45.70**	**± 0.33<br>± 0.73%**	1.60<br>13%	17.48<br>9	
				Full External Error	± 1.08		2.07	2σ Confidence Limit	
				Analytical Error	± 0.31		1.2661	Error Magnification	
**Inverse Isochron**<br>Clustered Points	**293.06**	**± 86.10<br>± 29.38%**	14.41484	± 0.09634<br>± 0.67%	**45.73**	**± 0.33<br>± 0.71%**	1.50<br>16%	17.48<br>9	
				Full External Error	± 1.08		2.07	2σ Confidence Limit	
				Analytical Error	± 0.30		1.2264	Error Magnification	
							3%	Spreading Factor	

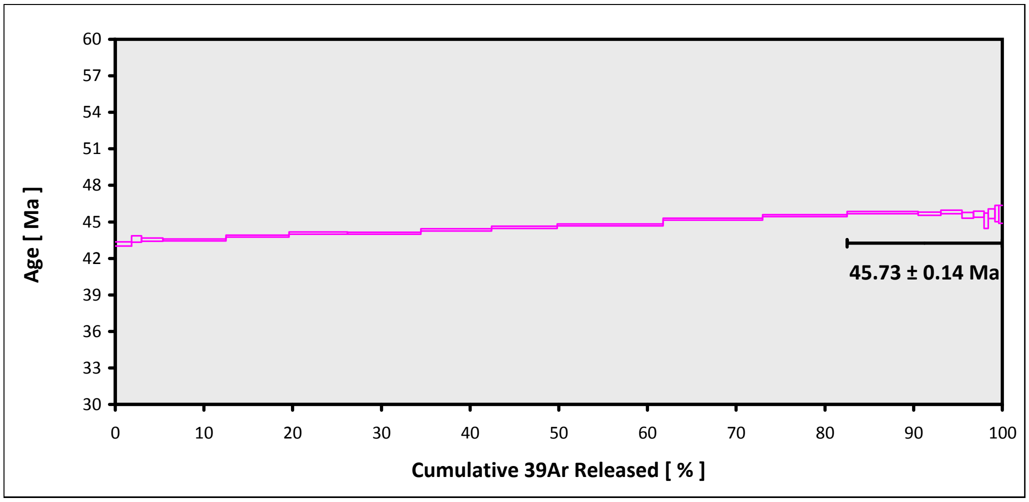

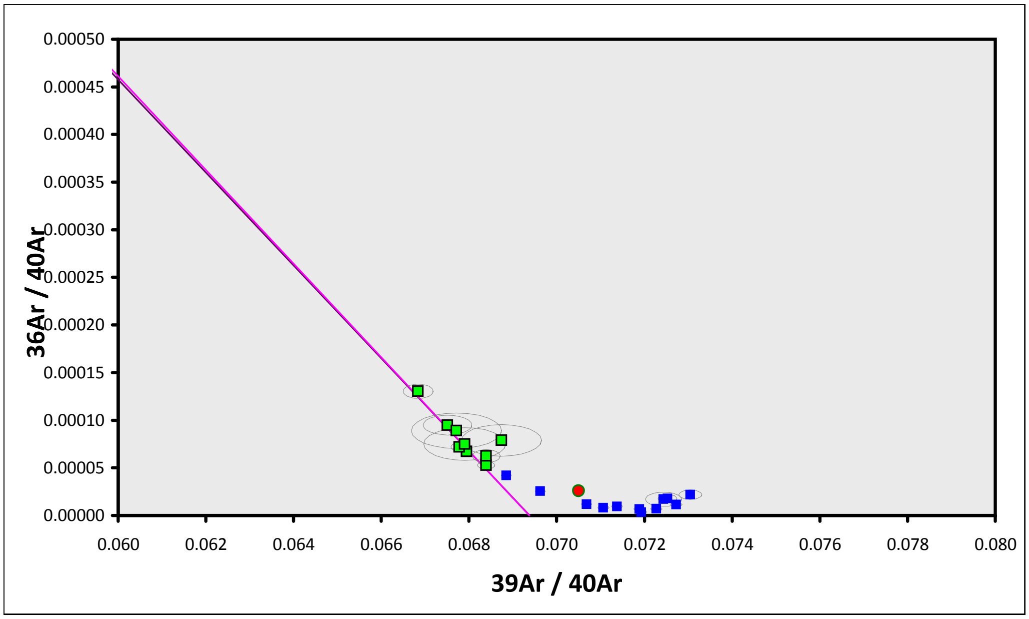

Supplementary References:

1 Konter, J. G. *et al.* One hundred million years of mantle geochemical history suggest the retiring of mantle plumes is premature. *Earth and Planetary Science Letters* **275**, 285-295, doi:10.1016/j.epsl.2008.08.023 (2008).

2 Wessel, P. & Kroenke, L. W. Pacific absolute plate motion since 145 Ma: An assessment of the fixed hot spot hypothesis. *Journal of Geophysical Research: Solid Earth (1978–2012)* **113** (2008).

3 Jackson, M. G. *et al.* Samoan hot spot track on a “hot spot highway”: Implications for mantle plumes and a deep Samoan mantle source. *Geochemistry, Geophysics, Geosystems* **11**, doi:10.1029/2010gc003232 (2010).

4 Koppers, A. A., Staudigel, H., Wijbrans, J. R. & Pringle, M. S. The Magellan seamount trail: implications for Cretaceous hotspot volcanism and absolute Pacific plate motion. *Earth and Planetary Science Letters* **163**, 53-68 (1998).

5 Koppers, A. A. P., Staudigel, H. & Duncan, R. A. High-resolution 40Ar/39Ar dating of the oldest oceanic basement basalts in the western Pacific basin. *Geochemistry, Geophysics, Geosystems* **4** (2003).

6 Koppers, A. A. P., Staudigel, H., Phipps Morgan, J. & Duncan, R. A. Nonlinear 40Ar/39Ar age systematics along the Gilbert Ridge and Tokelau Seamount Trail and the timing of the Hawaii-Emperor Bend. *Geochemistry, Geophysics, Geosystems* **8**, doi:10.1029/2006gc001489 (2007).

7 Wessel, P., Harada, Y. & Kroenke, L. W. Toward a self-consistent, high-resolution absolute plate motion model for the Pacific. *Geochemistry Geophysics Geosystems* **7**, doi:10.1029/2005gc001000 (2006).

8 Finlayson, V., Konter, J., Konrad, K., Koppers, A. & Jackson, M. in *AGU Fall Meeting Abstracts.* 4883.

9 Hart, S., Hauri, E., Oschmann, L. & Whitehead, J. Mantle plumes and entrainment: isotopic evidence. *Science* **256**, 517 (1992).

10 Jackson, M. *et al.* Helium and lead isotopes reveal the geochemical geometry of the Samoan plume. *Nature* **514**, 355-358 (2014).

11 Bonneville, A. *et al.* Arago Seamount: The missing hotspot found in the Austral Islands. *Geology* **30**, 1023-1026 (2002).

12 Dalrymple, G. B., Jarrard, R. & Clague, D. K-Ar ages of some volcanic rocks from the Cook and Austral Islands. *Geological Society of America Bulletin* **86**, 1463-1467 (1975).

13 Diraison, C. *Le volcanisme aérien des archipels polynésiens de la Société, des Marquises et des Australes-Cook. Téphrostratigraphie, datation isotopique et géochimie comparées. Contribution à l'étude des origines du volcanisme intraplaque du Pacifique Central*, (1991).

14 Duncan, R. A. & McDougall, I. Linear volcanism in French polynesia. *Journal of volcanology and geothermal research* **1**, 197-227 (1976).

15 Krummenacher, D. & Noetzlin, J. Ages isotopiques K-Ar de roches prélevées dans les possessions françaises du Pacifique. *Bull. Soc. Geol. Fr* **8**, 173-175 (1966).

16 Matsuda, J.-I., Notsu, K., Okano, J., Yaskawa, K. & Chungue, L. Geochemical implications from Sr isotopes and K-Ar age determinations for the Cook-Austral Islands chain. *Tectonophysics* **104**, 145-154 (1984).

17 Rose, J. J. M. An Evaluation of the Complex Age Progression along the Cook-Austral Islands Using High-resolution $^{40}Ar/^{39}Ar$ Incremental Heating Ages. *Oregon State University Dissertation* (2015).

18 Turner, D. L. & Jarrard, R. D. K-Ar dating of the Cook-Austral island chain: A test of the hot-spot hypothesis. *Journal of Volcanology and Geothermal Research* **12**, 187-220 (1982).

19 Duncan, R. A. & Clague, D. A. in *The ocean basins and margins* 89-121 (Springer, 1985).

20 Koppers, A. A. P., Morgan, J. P., Morgan, J. W. & Staudigel, H. Testing the fixed hotspot hypothesis using 40Ar39Ar age progressions along seamount trails. *Earth and Planetary Science Letters* **185**, 237-252 (2001).

21 Steinberger, B. & Gaina, C. Plate-tectonic reconstructions predict part of the Hawaiian hotspot track to be preserved in the Bering Sea. *Geology* **35**, 407-410 (2007).

22 Doubrovine, P. V., Steinberger, B. & Torsvik, T. H. Absolute plate motions in a reference frame defined by moving hot spots in the Pacific, Atlantic, and Indian oceans. *Journal of Geophysical Research: Solid Earth* **117** (2012).

23 Raymond, C. A., Stock, J. M. & Cande, S. C. *Fast Paleogene motion of the Pacific hotspots from revised global plate circuit constraints*. (Wiley Online Library, 2000).

24 Koppers, A. A. P., Duncan, R. A. & Steinberger, B. Implications of a nonlinear 40Ar/39Ar age progression along the Louisville seamount trail for models of fixed and moving hot spots. *Geochemistry, Geophysics, Geosystems* **5**, doi:10.1029/2003gc000671 (2004).

25 Steinberger, B. Plumes in a convecting mantle: Models and observations for individual hotspots. *Journal of Geophysical Research: Solid Earth* **105**, 11127-11152 (2000).

26 Koppers, A. A., Staudigel, H. & Minnett, R. Seamount Catalog: seamount morphology, maps, and data files. *Oceanography* **23**, 37, doi:10.5670/oceanog.2010.88. (2010).

27 Smith, W. & Sandwell, D. Predicted bathymetry. New global seafloor topography from satellite altimetry. *Eos Trans. AGU* **77**, 315 (1996).

28 Amante, C. & Eakins, B. W. *ETOPO1 1 arc-minute global relief model: procedures, data sources and analysis*. (US Department of Commerce, National Oceanic and Atmospheric Administration, National Environmental Satellite, Data, and Information Service, National Geophysical Data Center, Marine Geology and Geophysics Division Colorado, 2009).

29 Sharp, W. D. & Renne, P. R. The 40Ar/39Ar dating of core recovered by the Hawaii Scientific Drilling Project (phase 2), Hilo, Hawaii. *Geochemistry, Geophysics, Geosystems* **6** (2005).

30 McDougall, I. Potassium-argon ages from lavas of the Hawaiian Islands. *Geological Society of America Bulletin* **75**, 107-128 (1964).

31 Naughton, J. J., MacDonald, G. A. & Greenberg, V. Some additional potassium-argon ages of Hawaiian rocks: the Maui volcanic complex of Molokai, Maui, Lanai and Kahoolawe. *Journal of Volcanology and Geothermal Research* **7**, 339-355 (1980).

32 Dalrymple, G. B., Lanphere, M. A. & Jackson, E. D. Contributions to the petrography and geochronology of volcanic rocks from the Leeward Hawaiian Islands. *Geological Society of America Bulletin* **85**, 727-738 (1974).

33 McDougall, I. Age of shield-building volcanism of Kauai and linear migration of volcanism in the Hawaiian island chain. *Earth and Planetary Science Letters* **46**, 31-42 (1979).

34 Dalrymple, G. B., Clague, D. A., Garcia, M. O. & Bright, S. W. Petrology and K-Ar ages of dredged samples from Laysan Island and Northampton Bank volcanoes, Hawaiian ridge, and evolution of the Hawaiian-Emperor chain. *Geological Society of America Bulletin* **92**, 884-933 (1981).

35 O'Connor, J. M. *et al.* Constraints on past plate and mantle motion from new ages for the Hawaiian-Emperor Seamount Chain. *Geochemistry, Geophysics, Geosystems* **14**, 4564-4584 (2013).

36 Sharp, W. D. & Clague, D. A. 50-Ma initiation of Hawaiian-Emperor bend records major change in Pacific plate motion. *Science* **313**, 1281-1284 (2006).

37 Dalrymple, G. & Garcia, M. Age and chemistry of volcanic rocks dredged from Jingu Seamount, Emperor Seamount chain. *Initial Rep. Deep Sea Drill. Proj* **55**, 685-693 (1980).

38 Duncan, R. A. & Keller, R. A. Radiometric ages for basement rocks from the Emperor Seamounts, ODP Leg 197. *Geochemistry, Geophysics, Geosystems* **5**, n/a-n/a, doi:10.1029/2004gc000704 (2004).

39 Koppers, A. A. P. *et al.* New 40Ar/39Ar age progression for the Louisville hot spot trail and implications for inter-hot spot motion. *Geochemistry, Geophysics, Geosystems* **12**, doi:10.1029/2011gc003804 (2011).

40 Koppers, A. A. P. *et al.* Limited latitudinal mantle plume motion for the Louisville hotspot. *Nature Geoscience* **5**, 911-917 (2012).

41 Kuiper, K. *et al.* Synchronizing rock clocks of Earth history. *Science* **320**, 500-504 (2008).

42 Konter, J. G. & Storm, L. P. High precision 87 Sr/86 Sr measurements by MC-ICP-MS, simultaneously solving for Kr interferences and mass-based fractionation. *Chemical Geology* **385**, 26-34 (2014).

43 Bonhommet, N., Beeson, M. H. & Dalrymple, G. B. A contribution to the geochronology and petrology of the island of Lanai, Hawaii. *Geological Society of America Bulletin* **88**, 1282-1286 (1977).

44 Min, K., Mundil, R., Renne, P. R. & Ludwig, K. R. A test for systematic errors in 40Ar/39Ar geochronology through comparison with U/Pb analysis of a 1.1-Ga rhyolite. *Geochimica et Cosmochimica Acta* **64**, 73-98 (2000).
